# Atomic Doping of Sr–Co–Ni on Tungsten Carbide Electrocatalyst for Synergistically Enhanced Water Splitting Performance

**DOI:** 10.1021/acsami.5c26103

**Published:** 2026-01-31

**Authors:** Naveen Karuppusamy, Shaktivel Manavalan, Shen-Ming Chen, Bih-Show Lou, Durairaj Mahendiran, Palanichamy Murugan, Michelle J. S. Spencer, Ta Thi Thuy Nga, Pandian Mannu, Chi-Liang Chen, Jyh-Wei Lee, Chung-Li Dong, Tse-Wei Chen

**Affiliations:** † Department of Materials Engineering, 56082 Ming Chi University of Technology, New Taipei 243, Taiwan; ‡ Department of Chemical Engineering and Biotechnology, 34877 National Taipei University of Technology, Taipei City 10608, Taiwan; § Chemistry Division, Center for General Education, 56081 Chang Gung University, Taoyuan 333, Taiwan; ∥ Department of Orthopaedic Surgery, New Taipei Municipal TuCheng Hospital, Chang Gung Memorial Hospital, Taoyuan 333, Taiwan; ⊥ Electrochemical Power Sources Division, 62396 CSIR-Central Electrochemical Research Institute, Karaikudi, Tamil Nadu 630003, India; # Academy of Scientific and Innovative Research (AcSIR), Ghaziabad 201002, India; ∇ School of Science, RMIT University, Melbourne, Victoria 3001, Australia; ○ ARC Centre of Excellence in Future Low-Energy Electronics Technologies (FLEET), RMIT University, Melbourne, Victoria 3001, Australia; ◆ Department of Physics, 34886 Tamkang University, Tamsui City 251, Taiwan; ¶ 57815 National Synchrotron Radiation Research Center, Hsinchu 30076, Taiwan; ☆ Center for Plasma and Thin Film Technologies, Ming Chi University of Technology, New Taipei 243, Taiwan; ⟁ Department of Marine Environmental Engineering, 4615 National Kaohsiung University of Science and Technology, Kaohsiung City 81157, Taiwan

**Keywords:** electrocatalysts, water electrolyzer, tungsten carbide, atomic doping, nonprecious transition metal

## Abstract

Electrocatalysts play a pivotal role in the quest for renewable hydrogen energy via water electrolysis. This study presents a novel approach to synthesizing atomically doped Sr, Co, and Ni atoms onto tungsten carbide (W_*x*_C) via van der Waals interactions. Through systematic first-principles calculations, we investigate the intricate interplay of s–d orbital coupling among Sr, Co, and Ni atoms, revealing their profound impact on the electronic structure of W_*x*_C. This reveals that the addition of Sr to Co and Ni atoms decreases the absorption energy of the intermediate, which enhances catalytic activity at the Co and Ni sites. It demonstrates the strategy of ultralow loading of Sr atoms onto nonprecious transition metals, reducing the energy barrier for the OER (overpotential of 300 mV) and enhancing HER performance (125 mV at 10 mA cm^–2^), with stability maintained for 60 h. These findings underscore the potential of atomically engineered electrocatalysts in advancing sustainable hydrogen production.

## Graphical abstract

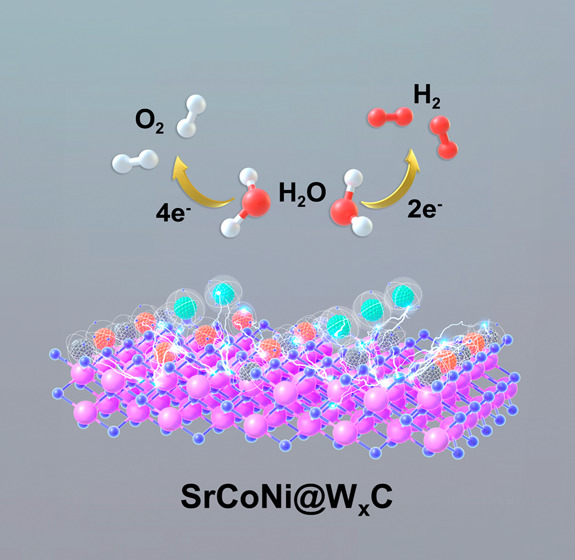

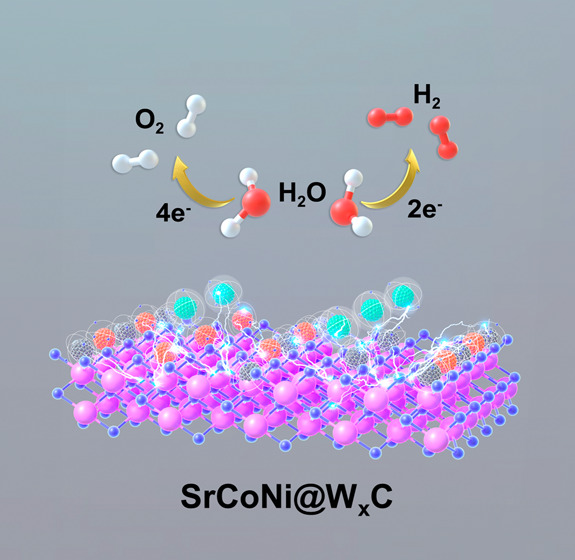

## 1 Introduction

Developing technologies are addressing the increasing energy demands amid the scarcity of fossil fuels, also the burning of fossil fuels leads to the emission of greenhouse gases.^1^ This has prompted the exploration of renewable energy sources as alternatives to mitigate environmental deterioration. Overall water splitting (OWS) comprising the oxygen evolution reaction (OER, anodic) and hydrogen evolution reaction (HER, cathodic) can separate the oxygen and hydrogen (thermodynamic value, 1.23 V) to the industrial-level production of hydrogen gas.^2^ It requires the electrocatalyst to have moderate binding energies with the oxygen intermediates (*OH, *OOH, *O), which accelerates the rate of reaction to produce high current density.^3^ Although noble metals such as Ru, Pt, and Ir have excellent inherent catalytic activity in water splitting,^4^ their high cost, low abundance, and long-term stability issues due to metal dissolution hinder their widespread usage for the OWS.^5^

Transition metals exhibit excellent electrocatalytic activity, undergo self-reconstruction during electrocatalysis, and are both abundant and cost-effective for commercialization.^6^ Consequently, a wide range of transition metal-based catalysts have been investigated for the OER and HER, including oxides,^7^ hydroxides,^8^ layered double hydroxides,^9^ sulfides,^10^ phosphides,^11^ nitrides,^12^ selenides,^13^ and carbides.^14^ Despite their promise, many of these materials exhibit sluggish kinetics, low intrinsic activity, high corrosion susceptibility, and poor durability, often rendering them inferior to noble metals. Notably, transition metal carbides stand out due to their exceptional corrosion stability in both low and high pH of electrolytes.^15^ Maintaining morphology under extreme operational conditions is crucial for sustaining electrocatalytic performance. Microstructures with high interfacial surface area can enhance the adsorption and desorption of intermediates, facilitating the rapid release of gas bubbles.^16^ Furthermore, developing atomically dispersed electrocatalysts can effectively increase the coordination of metal centers, maximizing atomic utilization during catalysis.^17^ In alkaline conditions, Ni nanoparticles transform into Ni­(OH)_2_, which serves as the active phase for the OER process and possesses moderate binding energies for the HER intermediate.^18^ To further support the HER activity, Co nanoparticles are a promising candidate, widely recognized for their lower energy barriers in the HER process.^19^ While various methods have been employed to modulate the electronic structure of these catalysts, alkali metal doping remains a particularly effective strategy. Therefore, the primary objective of this study is to introduce an alkali metal into the Ni–Co lattice to synergistically modulate the electronic structure and optimize the adsorption of intermediates, thereby establishing a robust, bifunctional electrocatalyst for efficient OER and HER in an alkaline water electrolyzer.

In the present work, we prepared the SrCoNi@W_*x*_C catalyst by carbonizing a polymerized dopamine complex under a nitrogen atmosphere. Incorporating an alkali metal into the transition metal effectively modulates the electronic structure of the transition metal and achieves the atomic-level dispersion of Sr, Co, and Ni onto the WC electrocatalyst, as confirmed by X-ray absorption spectroscopy (XAS) and density functional theory (DFT) calculations. The heterostructure of SrCoNi@W_*x*_C with the large surface area benefiting from high mass transfer and the cooperative effect of Sr, Co, and Ni boosted the catalytic activity, leading to the superior OER and HER performance.

## 2 Experimental Section

### 2.1 Materials and Reagents

Strontium­(II) chloride hexahydrate (SrCl_2_·6H_2_O, ACS reagent, 99%), cobalt­(II) chloride hexahydrate (CoCl_2_·6H_2_O ACS reagent, 98%), nickel­(II) chloride hexahydrate (NiCl_2_·6H_2_O, 99.9% trace metals basis), dopamine hydrochloride ((HO)_2_C_6_H_3_CH_2_CH_2_NH_2_·HCl), sodium tungstate dihydrate (Na_2_WO_4_·2H_2_O, ACS reagent, ≥99%), and hydrochloric acid (ACS reagent, 37%).

### 2.2 Preparation of SrCoNi@W_*x*_C

In beaker A, 15 mmol of (HO)_2_C_6_H_3_CH_2_CH_2_NH_2_·HCl is dissolved in 100 mL of DI water, and the pH of the solution is adjusted to 2 with 1 M HCl. Subsequently, 0.75 mmol of NiCl_2_·6H_2_O, CoCl_2_·6H_2_O and 0.50 mmol of SrCl_2_. 6H_2_O was added to the solution, which was stirred to achieve homogeneity. In beaker B, 15 mmol of Na_2_WO_4_. 2H_2_O was dissolved in 100 mL of DI water and stirred to get a homogeneous solution. The solution in beaker B is added drop by drop (for uniform mixing) to the homogenized solution in beaker A. The solution in beaker A yielded the yellowish-orange precipitate and was stirred for 1 h. Then, the precipitate was washed with DI water and ethanol three times and collected by centrifugation. The collected precipitate was air-dried overnight at 65 °C and then carbonized in a tube furnace in a N_2_ atmosphere at 900 °C, yielding SrCoNi@W_*x*_C-900. For comparison, the sample was carbonized at 800 and 700 °C and the control samples SrCo@W_x_C-900, SrNi@W_x_C-900, CoNi@W_x_C-900, Sr@W_x_C-900, Co@W_x_C-900, Ni@W_x_C-900, and W_x_C-900 are also prepared by the same method with their respective metal precursors.

## 3 Results and Discussion

### 3.1 Physicochemical Properties of SrCoNi@W_*x*_C

The preparation of SrCoNi@W_*x*_C is schematically illustrated in Figure 1a. Polymerization of dopamine is initiated by adjusting the pH. The reaction between WO^2–^ and metal ions results in the formation of a yellowish-orange precipitate, likely consisting of SrCoNi–tungstate complexes. The dried SrCoNi–tungstate complexes undergo carbonization at 900 °C in an N_2_ atmosphere, leading to the reduction of tungstate species to tungsten carbide (W_*x*_C). From the XRD spectra, the phases of WC, and W_2_C (hereafter both WC and W_2_C denoted as W_*x*_C or mentioned elsewhere) can be seen in Figure 1b. The atomic doping of Sr, Co, and Ni metal atoms is not observed from the XRD pattern, and the XRD patterns of the prepared control samples are provided in Figure S1, Supporting Information. FE-SEM and HR-TEM images of the dopamine complex before and after the carbonization reveal a microflower morphology (Figure 1c–f), indicating the strong potential for immobilization of the WC matrix on the carbon network. In addition, the high-magnification HR-TEM image (Figure 1g) shows the WC clusters, lattice fringes with 0.262 nm observed for the W_2_C, and the bright spot in the SAED pattern of SrCoNi@W_*x*_C (Figure 1h) having the hexagonal system of W_2_C with the space group of *P*3̅*m*1 manifested with the XRD data. The absence of WC lattice fringes may be attributed to the preferential exposure of W_2_C in the analyzed regions or differences in the structural stability under electron beam irradiation. The elemental mapping for SrCoNi@W_*x*_C-900 with the corresponding Sr, Co, Ni, W, and C elements (Figure 1i–m), respectively. When changing the doping condition with respect to the temperature, the microflower morphology of SrCoNi@W_*x*_C is retained. Besides, the BJH adsorption–desorption curve shows noncarbidic carbon over the SrCoNi@WC-700 surface reflects with the specific surface area than SrCoNi@W_*x*_C-900 and its mesoporous nature accelerates the mass transport properties in an alkaline electrolyte (Figure S2, Supporting Information). The FE-SEM images and the BJH adsorption–desorption curve of control samples are given in Figures S3–S11, Supporting Information. The specific surface area, pore volume, and pore diameter for all the samples are summarized in Table S1, Supporting Information.

**Figure 1:**
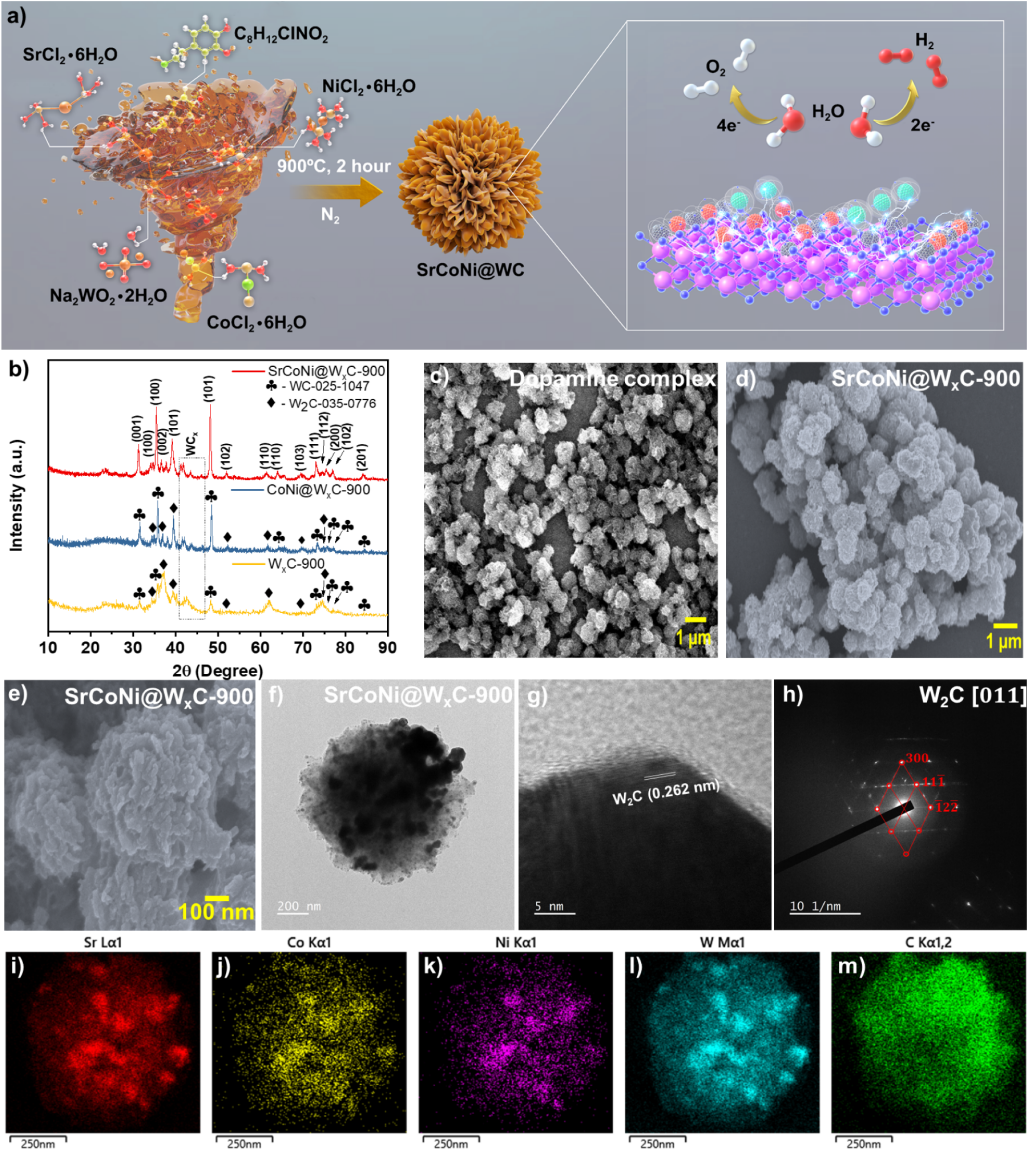
a) Graphical illustration for the preparation of SrCoNi@W*_x_* C. b) XRD pattern of SrCoNi@W*_x_* C-900, CoNi@W*_x_* C-900, and W*_x_* C-900. c–e) FE-SEM images of the dopamine complex after carbonization at 900 °C with different magnifications, respectively. f, g) HR-TEM, h) SAED pattern image of SrCoNi@W*_x_* C-900, and the corresponding individual elemental mapping images of i) Sr, j) Co, k) Ni, l) W, and m) C elements, respectively.

The graphitic nature of the carbonized samples was analyzed using the typical D- and G-band of Raman spectra shown in Figure 2a and Figure S12a, Supporting Information. The *I*_D_/*I*_G_ ratio of the carbonized samples ranges from 1.01 to 1.03, and it shows the existence of graphitic carbon other than carbidic carbon. As depicted in Figure S12b and Supporting Information, the X-ray photoelectron spectroscopy (XPS) analysis of the carbonized sample suggests that the Sr, Co, and Ni elements are not observed in the survey spectrum. However, the atomic weight (%) composition analysis provides evidence of the presence of constituent elements, as listed in Table S2 and Supporting Information. The results of ICP-MS analysis indicate that elemental percentages are not precisely observed with less than 1 ppm. Besides, the weight of the W atom is precisely identified and listed in Table S3, Supporting Information. The high-resolution XPS spectra of W 4f and C 1s (Figure 2b,c) show distinct peaks for the W–C bond and W–O bond, where W–O is observed for the surface oxidation to WO_3_. The atomic doping of Sr, Co, and Ni atoms onto WC results in a pronounced negative shift in the binding energy of W 4f compared to the pristine sample. The sharp and symmetrical peak of C 1s around the binding energy of 284.7 eV indicates the existence of amorphous carbon with the dominant graphitic character of SrCoNi@W_*x*_C in Figure 2c.

**Figure 2:**
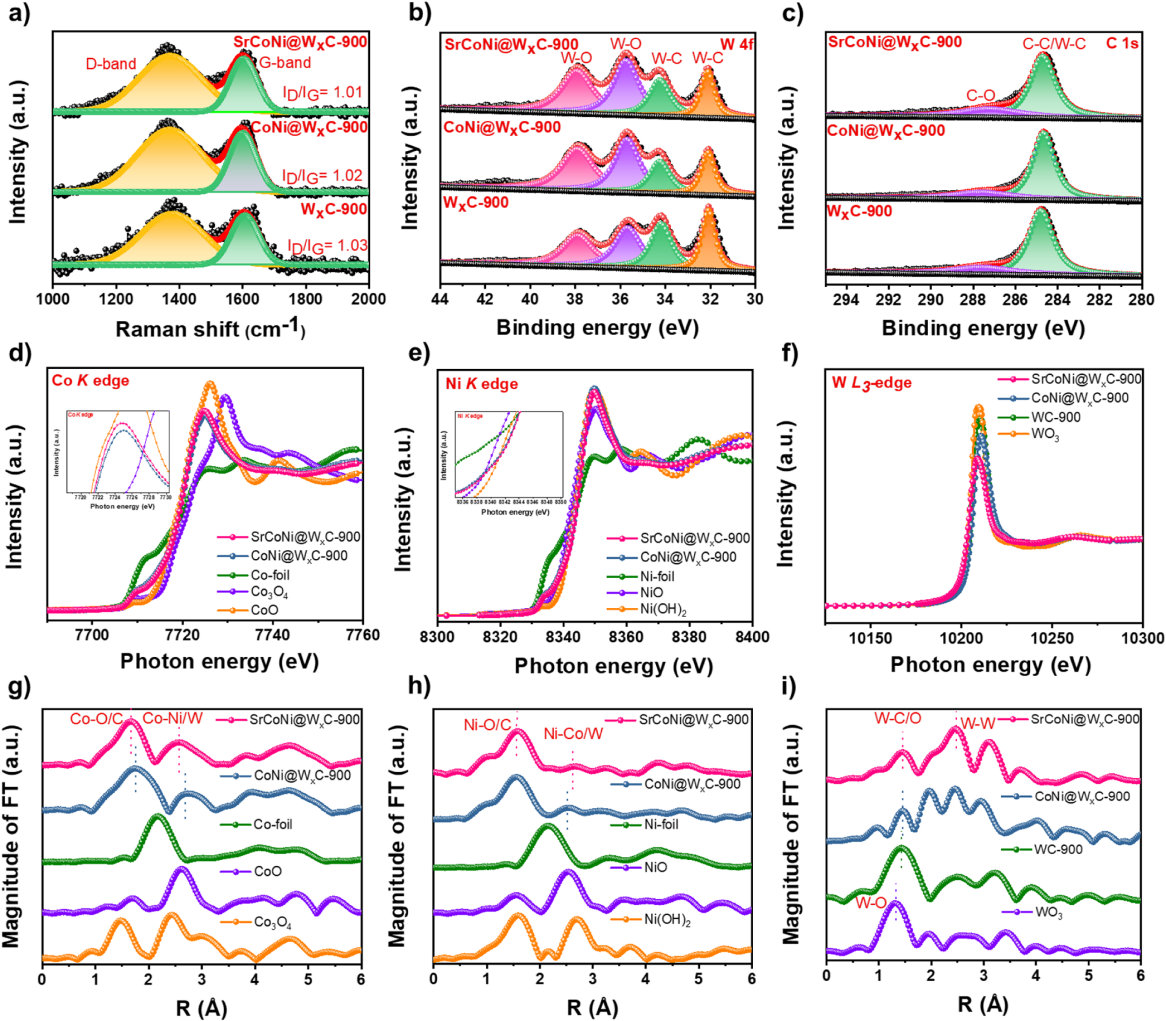
a) Raman spectra and b, c) high-resolution XPS spectra of W 4f and C 1s of SrCoNi@W*_x_* C-900, CoNi@W*_x_* C-900, and W*_x_* C-900. The local coordination analysis of SrCoNi@W*_x_* C-900, CoNi@W*_x_* C-900, and W*_x_* C-900 is scrutinized using d) Co K-edge, e) Ni K-edge, and f) W K-edge XANES spectra along with their corresponding g–i) R-space images, respectively.

The electrocatalytic activity is normally anticipated to be strongly interrelated with the local and electronic structures of Ni, Co, and W in the prepared catalysts. Therefore, the X-ray absorption near-edge spectroscopy (XANES) and X-ray absorption fine structure spectroscopy (EXAFS) are performed to understand the detailed local coordination among Sr, Co, Ni, W, and C atoms and their respective electrocatalytic activity.^20–22^ First, both the energy position and pre-edge peak intensity at the XANES Co K-edge are associated with the local symmetry and valence state of Co.^23–26^ For both SrCoNi@W_*x*_C and CoNi@W_*x*_C samples, the absorption edge position and the main peak positions are nearly close to that of the standard CoO (Co^2+^ oxidation state), as shown in Figure 2d. However, the higher intensity at the main absorption peak of SrCoNi@W_*x*_C compared to CoNi@W_*x*_C indicates the existence of higher charge states (Figure 2d, inset).

Additionally, in the case of tetrahedral symmetry, a strong pre-edge peak feature and a smooth white line are distinctive of a noncentrosymmetric structure. In contrast, low pre-edge peak intensity and a more distinct white line are indications of an octahedral symmetry.^23–26^ Meanwhile, Figure 2e displays the Ni K-edge XANES spectra of SrCoNi@W_*x*_C and CoNi@W_*x*_C along with the standard reference Ni foil, Ni­(OH)_2_, and NiO, respectively. Both SrCoNi@W_*x*_C and CoNi@W_*x*_C samples exhibit similar features related to the NiO XANES spectra, revealing an average positive valence of the Ni^2+^ state. In particular, the absorption edge position of SrCoNi@W_*x*_C was close to NiO and slightly shifted to higher energy as compared to those of CoNi@W_*x*_C, suggesting the presence of a higher oxidation state after integrating foreign Sr atoms (shown in Figure 2e, inset). All the above XANES results suggest that the introduction of Sr atoms to the CoNi@W_*x*_C heterostructure can successfully regulate the local electronic environment of Co, Ni, and W sites, in agreement with the present theoretical investigations (discussed below). Further, the normalized W L-edge of XANES and EXAFS spectra of the prepared catalysts (SrCoNi@W_*x*_C and CoNi@W_*x*_C) were recorded, and the results are presented in Figure 2f. Here, the strong absorption peak at the top of the edge is considered the “main absorption peak”, and therefore, the electronic density of empty d states can be observed by evaluating the intensity of the main absorption peak of W L-edge.^27^ It is noticeable that the white line intensity of the main absorption peak of the W L-edge of SrCoNi@W_*x*_C was lower compared to W_*x*_C and CoNi@W_*x*_C, as displayed in Figure 2f, which indicates a confinement of electron density at the tungsten atoms of surface-confined SrCoNi@W_*x*_C. In addition to that the SrCoNi@W_*x*_C sample shows a weaker white line lower-intensity absorption peak suggesting a higher electron density in the W 5d band than CoNi@W_*x*_C and pristine W_*x*_C.^28,29^

Additionally, the EXAFS spectra were evaluated to explore the atom coordination environments. The FT-EXAFS curves of the Co K-edge for CoNi@W_*x*_C exhibited two primary peaks located at ∼1.75 and 2.75 Å corresponding to the Co–O/C and Co/W, respectively (Figure 2g). Interestingly, the contraction in the bond length of Co–O/C and Co/W (∼1.66 Å and 2.57 Å) for SrCoNi@W_*x*_C heterostructure may be attributed to the Sr doping in CoNi@W_*x*_C that facilitated the locally distorted structure due to the formation of rich cation defects and anion vacancies.^30,31^ In the FT-EXAFS curve of Ni K-edge (Figure 2h), the peaks at ∼1.55 and 2.54 Å correspond to the Ni–O/C and Ni/W, respectively. Notably, there was a decrease in the peak intensity at the first M–O shell for SrCoNi@W_*x*_C compared with that of CoNi@W_*x*_C, again suggesting the existence of more cation defects and anion vacancies. Additionally, a peak at ∼3.25 Å was assigned to the Ni–W bond for SrCoNi@W_*x*_C and was reduced to ∼3.12 Å for the CoNi@W_*x*_C sample. Moreover, the bond distance of Ni–W (3.25 Å) was found to be much longer than that of the W–W bond (2.49 Å).

Furthermore, the EXAFS of the W L-edge of WC shows the appeared peaks at 1.43 and 2.49 Å that are assigned to W–C and W–W bonds, respectively (Figure 2i). For both SrCoNi@W_*x*_C and CoNi@W_*x*_C samples, the bond length of W–C (1.46 Å) was found to be slightly increased (elongation) compared to that of the pristine WC sample, suggesting that after depositing CoNi monolayer on the WC monolayer (to form the CoNi–W_*x*_C heterostructure) results in a weak interaction between the layers, which is consistent with the present theoretical study. A similar conclusion was also evidenced by Ni, Co K-edge, and W L-edge k3 χ data of EXAFS oscillations (Figures S13–S15, Supporting Information) that show different EXAFS oscillations (also low intensity EXAFS oscillations) for SrCoNi@W_*x*_C compared to other samples, indicating the different coordination geometry around Sr, Ni, Co, W, and C sites. Moreover, this obvious difference demonstrates the introduction of Sr significantly influenced the local coordination environment of the Ni/Co/W/C atom and produced more disorder in the local electronic structure. The fitted EXAFS graph and parmeters are given in Figure S16, Supporting Information and Table S4.

### 3.2 Investigation of Catalytic Efficiency for Oxygen Evolution Reaction

The study of atomic doping of Sr, Co, and Ni atoms onto the WC is tested with the alkaline OER performance using the standard three-electrode setup at room temperature with 1 M KOH. The linear sweep voltammetry (LSV) polarization curve for SrCoNi@W_*x*_C-900 was obtained without IR correction using a scan speed of 5 mV/s, as displayed in Figure 3a. The superior OER performance of SrCoNi@W_*x*_C-900 surpasses the benchmark RuO_2_ and achieves the current density of 10 mA cm^–2^ with a smaller overpotential of 300 mV than the control samples such as SrCoNi@W_*x*_C-800 (337 mV), SrCoNi@W_*x*_C-700 (329 mV), SrCo@W_*x*_C-900 (341 mV), SrNi@W_*x*_C-900 (359 mV), and CoNi@W_*x*_C-900 (339 mV). The lower Tafel slope values of SrCoNi@W_*x*_C-900 (132 mV dec^–1^) than SrCoNi@W_*x*_C-800 (138 mV dec^–1^), SrCoNi@W_*x*_C-700 (142 mV dec^–1^), SrCo@W_*x*_C-900 (134 mV dec^–1^), SrNi@W_*x*_C-900 (150 mV dec^–1^), and CoNi@W_*x*_C-900 (128 mV dec^–1^) unraveling the effect of introduction of Sr atom to the CoNi@W_*x*_C-900 boosting the catalytic activity in the OER performance Figure 3b. The polarization curves for the other control samples and its Tafel plot are portrayed in Figure S17, and Supporting Information. Further, the OER performance is investigated by varying the Sr composition in SrCoNi@W_*x*_C-900 (Figure S18, Supporting Information), and it reveals that the appropriate amount of Sr is beneficial for the modulation of the electronic structure of CoNi@W_*x*_C-900, which improves the electrocatalytic by reducing the energy barrier activity of the catalyst. Moreover, SrCoNi@W_*x*_C-900 demonstrates a superior turnover frequency per real active (TOFreal) of 0.045 s^–1^ than the pristine WC matrix (Figure S19, Supporting Information). In Figure 3c, the bar graph depicts a comparison of the Tafel slope and overpotential for the OER process. The overall comparison of overpotential and Tafel slope values of all the control electrodes for OER performance are listed in Table S5, Supporting Information. To realize the electrochemical active surface area (ECSA) of the electrode, the CV was performed under the nonfaradic region to calculate the double-layer capacitance (*C*_dl_) reflecting the exposed sites in electrochemical performance (Figure S20, Supporting Information). As seen in Figure 3d, SrCoNi@W_*x*_C-900 (4.6 mF cm^–2^) has relatively more *C*_dl_ than SrCoNi@W_*x*_C-800 (2.1 mF cm^–2^), SrCoNi@W_*x*_C-700 (2.0 mF cm^–2^), SrCo@W_*x*_C (2.2 mF cm^–2^), SrNi@W_*x*_C (2.1 mF cm^–2^), and CoNi@W_*x*_C (2.7 mF cm^–2^) suggest that the SrCoNi@W_*x*_C-900 accelerates the OER performance with more number of active sites. The calculated ECSA of all the electrodes is listed in Table S6 and Supporting Information. The electrochemical impedance spectroscopy (EIS) was performed to study the kinetics of electron transfer at the interface of the electrode surface, the Nyquist plots are obtained with the help of a Randles equivalent circuit. As seen in Figure 3e, lower charge transfer resistance (*R*_ct_) was exhibited by the SrCoNi@W_*x*_C-900 (0.42 Ω) than control electrodes, ascribed to the superior kinetics in the OER performance. The other parameters such as solution resistance (*R*_s_) and double-layer capacitance (*C*_dl_) are listed in Table S7, Supporting Information, for all of the electrodes. The overpotential and Tafel slope activity trend of SrCoNi@W_*x*_C-900 is compared against the amount of Sr used for the synthesis displayed in Figure 3f. It shows decrease in overpotential when increase the Sr (33 to 66 mg) and again it starts to increase upon increasing Sr concentration. The long-term stability analysis for SrCoNi@W_*x*_C-900 analyzed with the chronopotentiometry (CP) for 60 h of OER performance under current densities of 10, 30, and 50 mA cm^–2^ switched for every 20 h (Figure 3g) shows the negligible changes in the overpotential of the aforementioned current densities. The LSV polarization curves of SrCoNi@W_*x*_C-900 after the stability performances (Figure S21, Supporting Information) with a smaller amount of loss in overpotential, besides no changes in the morphology observed with the TEM and FE-SEM images (Figures S22, S23, Supporting Information), suggest the robust stability of SrCoNi@W_*x*_C-900. Additionally, SrCoNi@W_*x*_C-900 is tested with a higher current density (@200 mA cm^–2^) for 60 h, which shows that the overpotential has increased over a period of time (Figure S24, Supporting Information). The comparison of the OER performance with the reported literature is listed in Table S8, Supporting Information.

**Figure 3:**
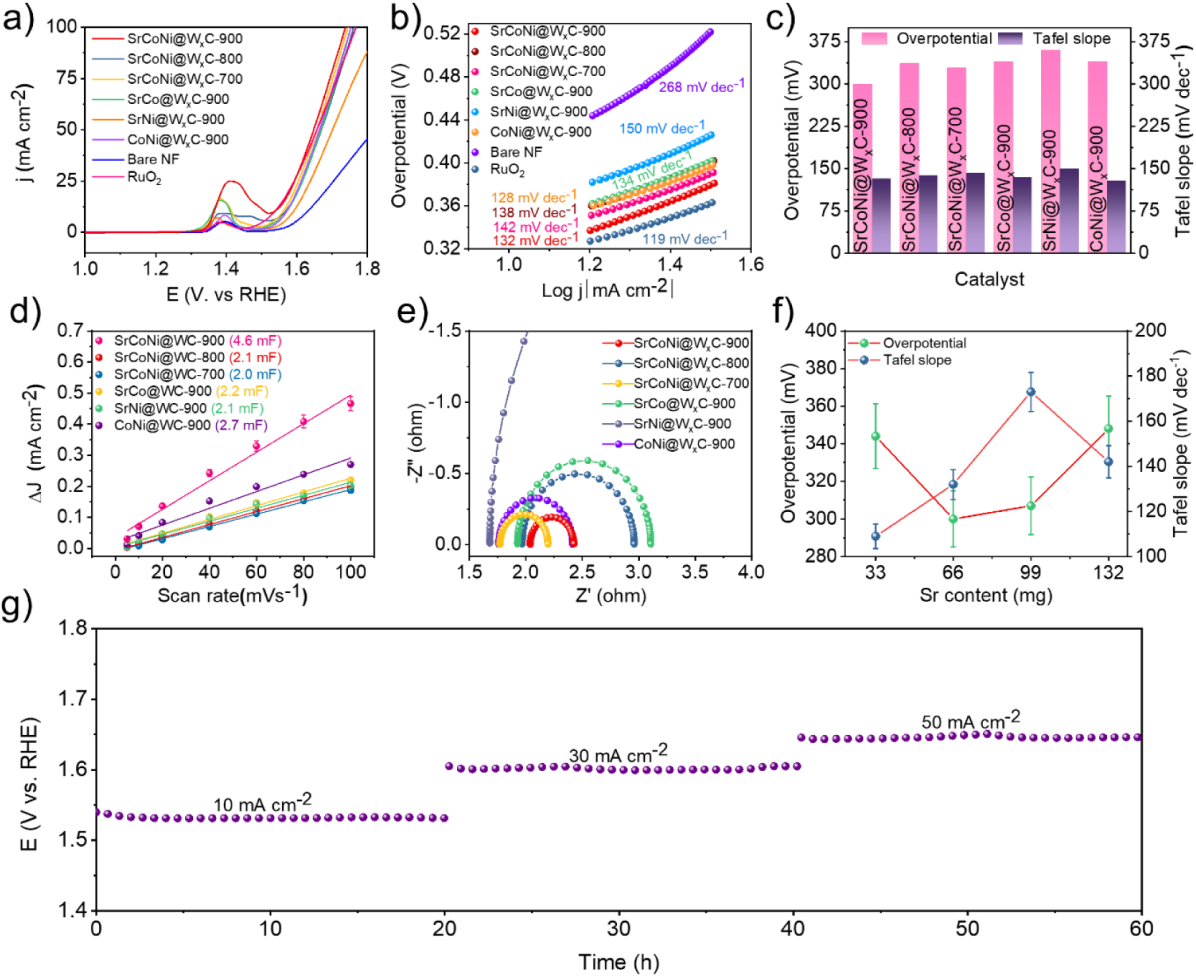
a) LSV polarization and b) Tafel slope for the OER performance of SrCoNi@W*_x_* C-900, SrCoNi@W*_x_* C-800, SrCoNi@W*_x_* C-700, SrCo@W*_x_* C-900, SrNi@W*_x_* C-900, CoNi@W*_x_* C-900, bare NF, and RuO_2_ samples are analyzed using 1 M KOH with a scan rate of 5 mV s^–1^ is recorded without the *iR* correction. c) OER work function comparison with its overpotential at 10 mA cm^–2^ and Tafel slope. d) Linear plot of scan rate versus capacitive current and e) the EIS spectra of SrCoNi@W*_x_* C-900, SrCoNi@W*_x_* C-800, SrCoNi@W*_x_* C-700, SrCo@W*_x_* C-900, SrNi@W*_x_* C-900, and CoNi@W*_x_* C-900. f) Comparison of the overpotential and Tafel slope with respect to the weight of Sr in SrCoNi@W*_x_* C-900 preparation. g) Chrono-potentiometric curve of SrCoNi@W*_x_* C-900 for 60 h with current densities of 10, 30, and 50 mA cm^–2^ of each 20 h, respectively.

### 3.3 Investigation of Catalytic Efficiency for the Hydrogen Evolution Reaction

The HER investigation of SrCoNi@W_*x*_C was assessed with a 1 M KOH solution using a standard three-electrode setup. The state-of-the-art Pt/C and control samples are also investigated to compare the HER performance with SrCoNi@W_*x*_C. The HER polarization curves obtained with a scan rate of 5 mV s^–1^ are displayed (without IR correction) in Figure 4a, and the SrCoNi@W_*x*_C-900 catalyst requires a significantly low overpotential (125 mV) to reach 10 mA cm^–2^ than the control sample as follows: SrCoNi@W_*x*_C-800 (174 mV), SrCoNi@W_*x*_C-700 (175 mV), SrCo@W_*x*_C (238 mV), SrNi@W_*x*_C (199 mV), and CoNi@W_*x*_C (176 mV). The improved overpotential is observed for SrCoNi@W_*x*_C-900 compared to the W_*x*_C matrix (189 mV) and is solely attributed to the atomic dispersion of SrCoNi atoms. As seen in Figure 4b, the Tafel slope of SrCoNi@W_*x*_C-900 with 38 mV dec^–1^, remarkably lower than those of SrCoNi@W_*x*_C-800 (103 mV dec^–1^), SrCoNi@W_*x*_C-700 (80 mV dec^–1^), SrCo@W_*x*_C (102 mV dec^–1^), SrNi@W_*x*_C (100 mV dec^–1^), and CoNi@W_*x*_C (73 mV dec^–1^), reveals that SrCoNi@W_*x*_C-900 follows the Volmer–Heyrovsky mechanism for the HER process. In Figure 4c, the bar graph portrays a comparison of the Tafel slope and overpotential for the HER process. The HER polarization curve and Tafel slope for the control electrode are displayed in Figure S25 and Supporting Information, and the comparison of HER performance with the control samples is given in Table S9 and Supporting Information. From the CV curve (Figure S26, Supporting Information), the *C*_dl_ calculated on the nonfaradaic region suggests that SrCoNi@W_*x*_C-900 (112 mF cm^–2^) has more active sites than SrCoNi@W_*x*_C-800 (99 mF cm^–2^), SrCoNi@W_*x*_C-700 (5 mF cm^–2^), SrCo@W_*x*_C (72 mF cm^–2^), SrNi@W_*x*_C (76 mF cm^–2^), and CoNi@W_*x*_C (93 mF cm^–2^), as displayed in Figure 4d. From the *C*_dl_, the calculated ECSA is listed in Table S10 and Supporting Information. In Figure 4e, SrCoNi@W_*x*_C-900 (0.40 Ω) shows a lower charge transfer resistance (*R*_ct_) than other control electrodes (Table S11, Supporting Information) owing to its superior charge transfer activity. Further, the effect of Sr composition in CoNi@W_*x*_C is summarized in Figure 4f (observed in Figure S27, Supporting Information). SrCoNi@W_*x*_C-900 has a TOFreal of 0.005 s^–1^ at an overpotential of 300 mV (Figure S28, Supporting Information). As seen in Figure 4g, CP was performed with the stepping current density of 10, 30, and 50 mA cm^–2^ for every 20 h, showing the robust durability of SrCoNi@W_*x*_C, reducing the overpotential as observed in polarization curves after the long-term performance to the overpotential of mV. This demonstrates that SrCoNi@W_*x*_C-900 enables the number of active sites during long-term stability performances. In Figure S29 and Supporting Information, the LSV performance of SrCoNi@W_*x*_C-900 after the long-term stability test manifests with the reduction of overpotential in HER performance. The post-characterization of SrCoNi@W_*x*_C-900 after the stability performance observed with the TEM and FE-SEM images displayed in Figures S30, S31, Supporting Information shows the robustness of SrCoNi@W_*x*_C-900 while retaining the micro-flower morphology. Additionally, SrCoNi@W_*x*_C-900 is tested with a higher current density (@200 mA cm^–2^) for 60 h, showing excellent stability with overpotential decreased over period of time (Figure S32, Supporting Information). The overpotential increased by approximately 200 mV during the 60 h stability test at 200 mA cm^–2^. The harsh anodic potential required to sustain 200 mA cm^–2^ induces partial surface oxidation of the carbide, and this interfacial oxidation contributes to an increase in the ohmic resistance over time. The HER performance of SrCoNi@W_*x*_C-900 is compared with the reported literature in Table S12, Supporting Information.

**Figure 4:**
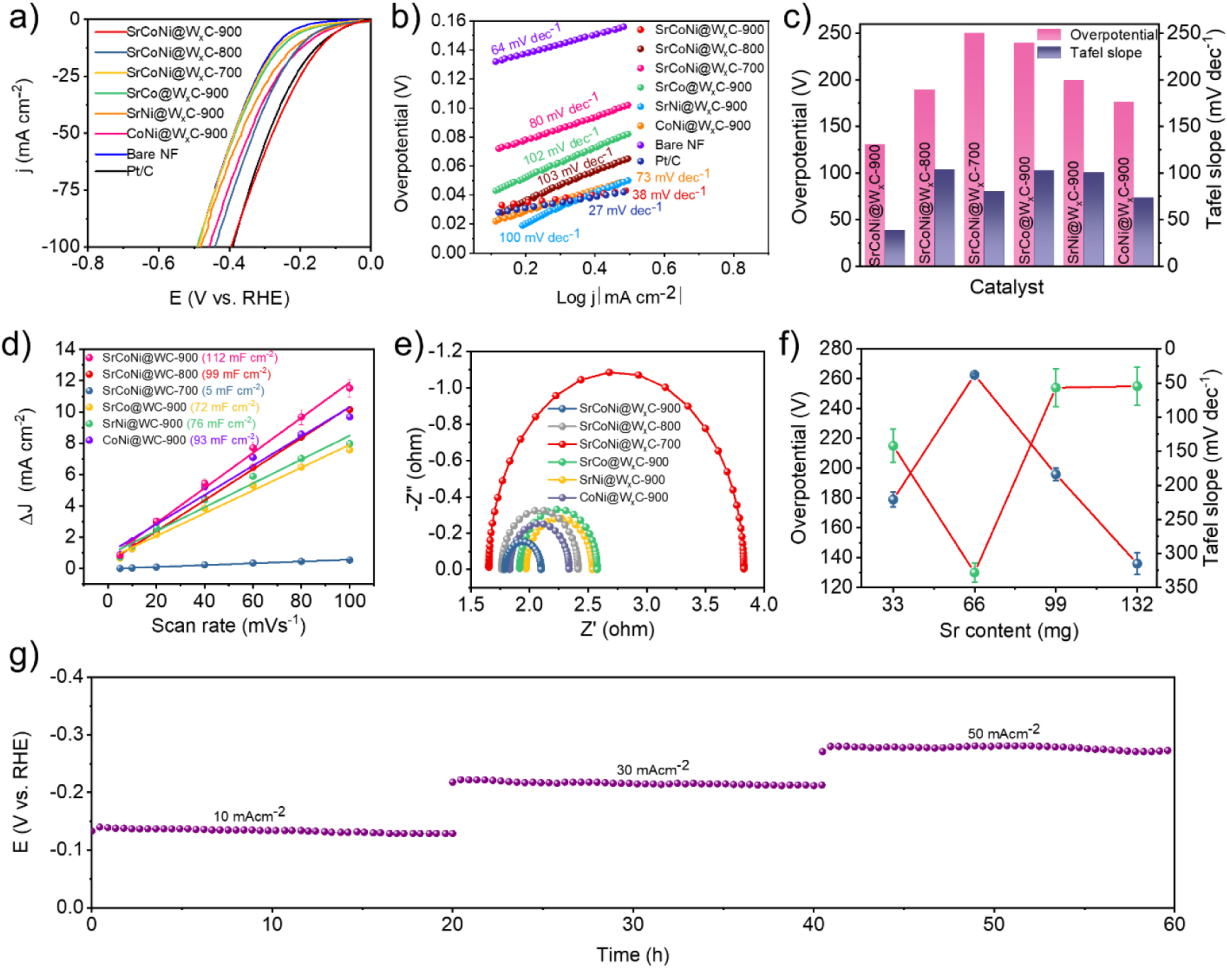
a) LSV polarization and b) Tafel slope for the HER performance of SrCoNi@W*_x_* C-900, SrCoNi@W*_x_* C-800, SrCoNi@W*_x_* C-700, SrCo@W*_x_* C-900, SrNi@W*_x_* C-900, CoNi@W*_x_* C-900, bare NF, and Pt/C samples are analyzed using 1 M KOH with a scan rate of 5 mVs^–1^ recorded without the *iR* correction. c) HER work function comparison with its overpotential at 10 mA cm^–2^ and Tafel slope, d) linear plot of scan rate versus capacitive current, and e) EIS spectra of SrCoNi@W*_x_* C-900, SrCoNi@W*_x_* C-800, SrCoNi@W*_x_* C-700, SrCo@W*_x_* C-900, SrNi@W*_x_* C-900, and CoNi@W*_x_* C-900. f) Comparison of overpotential and Tafel slope with respect to the weight of Sr in SrCoNi@W*_x_* C-900 preparation. g) Chronopotentiometric curve of SrCoNi@W*_x_* C-900 for 60 h with the current densities of 10, 30, and 50 mA cm^–2^ of each 20 h, respectively.

The alkaline water splitting electrolyzer performance with the bifunctional SrCoNi@W_*x*_C-900 electrocatalyst assembled as both the anodic and cathodic electrode materials was displayed by the two electrode polarization curves plotted in H-type Teflon cell with 1 M KOH, as shown in Figure S31, Supporting Information. It is compared with the state-of-the-art RuO_2_ and Pt/C as the cathodic electrode. The distinct bifunctional performance exhibited by SrCoNi@W_*x*_C-900 requires 1.73 V to reach 10 mA cm^–2^ observed in the polarization curve of SrCoNi@W_*x*_C-900∥SrCoNi@W_*x*_C-900. The combinations of electrodes were changed with RuO_2_ and Pt/C by replacing a cathode with the same anode (SrCoNi@W_*x*_C-900), whereas SrCoNi@W_*x*_C-900∥+RuO_2_ (1.61 V) shows a superior overall water splitting performance than SrCoNi@W_*x*_C-900∥+Pt/C (1.76 V). The comparison of overall water-splitting performance was compared with the reported literature in Table S13 and Supporting Information.

### 3.4 A Theoretical Perspective on Electrocatalytic Enhancement Mechanisms

Our experiments have revealed that introducing Sr dopants into the CoNi layer of the CoNi@W_*x*_C heterostructure significantly enhances its catalytic performance for the oxygen evolution reaction (OER) compared to the undoped heterostructure and individual layers. To validate these experimental findings, we performed first-principles-based density functional theory (DFT) calculations. In our study, a 4 × 4 × 1 supercell was used for both the pristine W_*x*_C (001) layer and the CoNi (001) layer (with a balanced Ni:Co ratio of 1:1), as illustrated in Figure S33, Supporting Information. The lattice parameters of the W_*x*_C and CoNi monolayers were calculated to be *a* = *b* = 12.16 and 11.38 Å, respectively. The lengths for the W–C (upper layer) and Ni–Co bonds were 2.03 Å and 2.85 Å, respectively. These values are consistent with the values reported in previous studies.^32,33^ To form the CoNi–W_*x*_C heterostructure, a CoNi monolayer was deposited on the W_*x*_C monolayer. There was a slight elongation of the W–C bond length (2.21 Å) after the formation of the HS due to the weak interactions between the layers, which was confirmed by the small binding energy of 0.23 eV/atom. To model the doped HS, 2Co and 2Ni atoms were replaced with 4Sr atoms to form the SrCoNi@W_*x*_C heterostructure. Several initial configurations of the Sr dopants on the heterostructure were considered to identify the most stable structure, as shown in Figure S34, Supporting Information and Table S14, Supporting Information. The predicted lowest-energy structure of the SrCoNi@W_*x*_C heterostructure is presented in Figure 5a, depicting both top and side views with the corresponding bond lengths, magnetic moment, and binding energy of the system reported in Table S15, Supporting Information. The construction of the heterostructure interface induces a redistribution of charge for the SrCoNi@W_*x*_C, and the results show that there is an accumulation of charge at the W_*x*_C layer, indicating electrons have been transferred from the SrCoNi layer to the W_*x*_C surface (refer to Figure 5b). This reconfiguration of charge facilitates the adsorption of oxygen species at active sites, which becomes dominant in the catalytic process. The electronic properties of the undoped and Sr-doped heterostructure were analyzed using partial density of states (PDOS), as illustrated in Figure 5c. For the undoped heterostructure, the occupied Ni 3d spin-up states are found to be predominantly around the Fermi level region. However, with the introduction of Sr to the heterostructure, there is an excess transfer of charge from Sr to the system, resulting in the Co atoms in the CoNi layer gaining charge. This charge transfer has a significant effect on the electronic structure of the system. In particular, the Co spin-up states become dominant around the Fermi level region. Moreover, the Co atoms in the CoNi layer act as active sites, signifying their enhanced reactivity and potential for catalytic applications.

**Figure 5:**
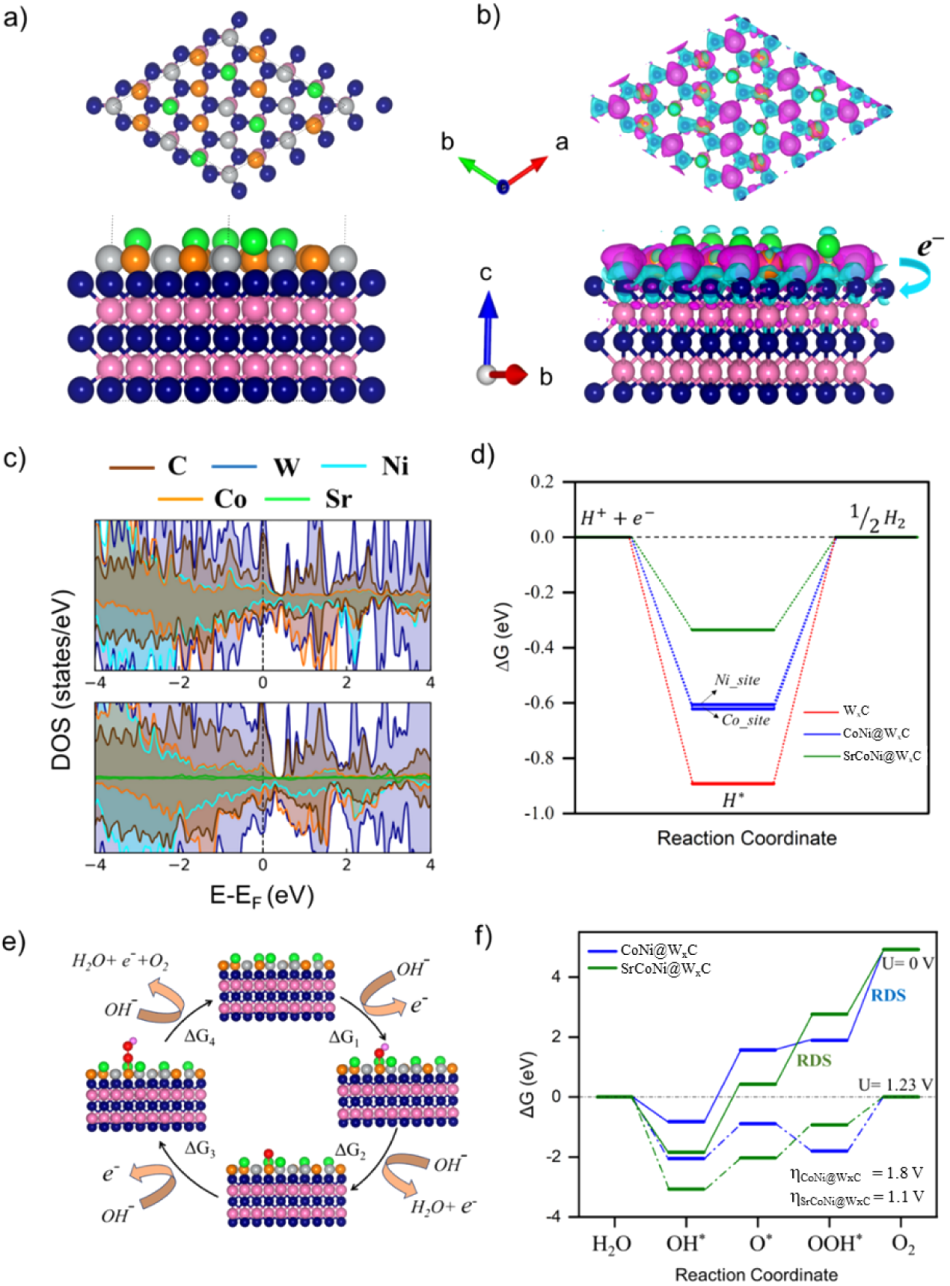
a) Top and lateral views for the SrCoNi@W*_x_* C heterostructure. The balls in blue, pink, gray, orange, and green denote W, C, Ni, Co, and Sr atoms, respectively. b) Corresponding electron density difference plot (CDD) of the heterostructure. The excess (depletion) of charge is represented by cyan (pink) isosurface (iso-surface value is 0.005 e^–^/Å^3^). c) Partial density of states of the doped and undoped heterostructure and d) the calculated free-energy diagram for the hydrogen evolution reaction (HER). e) Schematic diagram representing the oxygen evolution reaction (OER) mechanism on SrCoNi@W*_x_* C. f) Gibb’s free energy diagram for OER on CoNi@W*_x_* C (blue line) and SrCoNi@W*_x_* C (green line). The rate-determining step (RDS) is highlighted, and η represents the theoretical overpotential.

To investigate the hydrogen evolution activity of the HS, the change in free energy for adsorption of H (ΔGH*) on the isolated layers, as well as on the undoped and Sr-doped heterostructure, was performed. The adsorption free energy of H (ΔGH*) serves as a reliable indicator for hydrogen evolution activity. Here, an optimum ΔGH* value close to zero will suggest high HER activity. Figure 5d illustrates the calculated free energy diagram for HER on the isolated WC, with and without Sr doping. For the isolated W_*x*_C, ΔGH* is highly negative (−0.89 eV), indicating a strong interaction between adsorbed hydrogen (H*) and W_*x*_C, resulting in poor HER reaction kinetics. On the CoNi–W_*x*_C heterostructure, there are two active sites, namely, nickel and cobalt. The corresponding ΔGH* values for these sites are −0.62 and −0.61 eV, respectively, suggesting that the Ni and Co sites exhibit almost the same catalytic behavior. Furthermore, after Sr doping of the heterostructure, there is an increase in charge around the active sites due to excess charge on Sr that is transferred to the system. As a result, the ΔGH* values decrease to −0.33 eV, indicating that the Sr-doped heterostructure enhances the HER activity compared to both the individual layers and the undoped heterostructure. This suggests that introducing Sr dopants improves the catalytic properties, making the Sr-doped heterostructure a promising candidate for efficient HER applications.

The OER process was studied using a 4e^–^ mechanism proposed by Nørskov et al.^34^ For the Sr-doped and undoped heterostructure models, the Co atoms were selected as the active sites. The intermediates in the OER are listed in Figure 5e. The calculated free energy profiles for different external potentials are shown in Figure 5f. For the undoped heterostructure (blue line), at an external potential of 0 V, Δ*G*_1_ of the first step is negative, indicating that the formation of OH* from adsorbed H_2_O at the Co-active site can occur spontaneously. The rate-determining step (RDS) was found to be the formation of OOH* → O_2_ (step IV), with an energy barrier of 3.02 eV, resulting in a high theoretical overpotential of 1.8 eV. However, for the Sr-doped heterostructure, the energy barrier of the last step was significantly reduced, indicating that *OOH is adsorbed onto the catalytically active surface, and it is more feasible to convert into triplet state O_2_, as shown in Figure 5f (green line). Figure 5f shows that the higher barrier for the O to OOH step in SrCoNi directly translates to slower intrinsic electron transfer kinetics, which manifests experimentally as a larger Tafel slope for SrCoNi@W_*x*_C. Instantly, the largest Δ*G* value is the third step and is identified as the RDS due to the relatively weak OOH* adsorption on the Co site, with an energy barrier of 2.32 eV at *U* = 0 V. This observation remains consistent even at the equilibrium potential (*U* = 1.23 V). The resultant theoretical overpotential for SrCoNi@W_*x*_C is 1.1 V, which is smaller than that for CoNi@W_*x*_C. The theoretical overpotential value is slightly higher than the experimental value due to the simplified model used in this calculation. The DFT calculations support our EXAFS findings, affirming the beneficial role Sr plays in reducing the reaction barrier, as evidenced by the decrease in overpotential. The electrochemical results demonstrate the superiority of the SrCoNi@W_*x*_C catalyst toward the OER in terms of the electrochemical parameters, indicating its enhanced catalytic performance for the OER compared to the undoped heterostructure.

## 4 Conclusions

In conclusion, our study demonstrates the successful atomic doping of Sr, Co, and Ni onto W_*x*_C via the carbonization of the dopamine complex. Analysis using XAS and computational studies uncovers the electronic structure of W_*x*_C by facilitating s–d orbital coupling of Sr, Co, and Ni atoms, which effectively decreases the energy barrier while increasing the OER and HER performance. Notably, overpotentials of 300 and 130 mV for the OER and HER performance in 1 M KOH electrolyte are required to reach 10 mA cm^–2^, with Tafel slopes of 132 and 38 mV dec^–1^, respectively. This study opens up the strategy of s–d orbital coupling to effectively boost the bifunctional property of the catalyst, and this work lays a strong foundation for advancing atomically engineered electrocatalysts, with promising implications for sustainable hydrogen production and the development of efficient energy systems. Additionally, scaling the catalyst for industrial applications and integrating it into existing water electrolysis systems are key steps for practical implementation.

## Supplementary Material



## Notes

XRD, FE-SEM, XPS, and BET data of SrCoNi@W_*x*_C-900, SrCoNi@W_*x*_C-800, SrCoNi@W_*x*_C-700, SrCo@W_*x*_C-900, SrNi@W_*x*_C-900, CoNi@W_*x*_C-900, Sr@W_*x*_C-900, Co@W_*x*_C-900, Ni@W_*x*_C-900, and W_*x*_C-900; OER and HER results of Sr@W_*x*_C-900, Co@W_*x*_C-900, and Ni@W_*x*_C-900; crystal structures of SrCoNi@W_*x*_C-900; and supporting tables (PDF)
