# Supplementary material for: Atomic Doping of Sr–Co–Ni on Tungsten Carbide Electrocatalyst for Synergistically Enhanced Water Splitting Performance
Source: ACS Appl Mater Interfaces. 2026 Jan 31;18(5):8358–68. doi: 10.1021/acsami.5c26103 (PMC12903100; doi:10.1021/acsami.5c26103)
Supplement: Supplementary file 1 [file am5c26103_si_001.pdf]

Supporting Information

Atomic Doping of Sr-Co-Ni on Tungsten Carbide Electrocatalyst for Synergistically Enhanced Water Splitting Performance

Naveen Karuppusamy[a,b], Shaktivel Manavalan[b], Shen-Ming Chen[b,], Bih-Show Lou[c,d*], Durairaj Mahendiran[e,f,g], Palanichamy Murugan[e,f], Michelle J.S. Spencer[g,h], Ta Thi Thuy Nga[i], Pandian Mannu[i,j], Chi-Liang Chen[j], Jyh-Wei Lee[a,k], Chung-Li Dong[i,*], Tse-Wei Chen[l,*].*

[a] Department of Materials Engineering, Ming Chi University of Technology, New Taipei, 243, Taiwan.

[b] Department of Chemical Engineering and Biotechnology, National Taipei University of Technology, Taipei City, 10608, Taiwan.
*Email: smchen1957@gmail.com

[c] Chemistry Division, Center for General Education, Chang Gung University, Taoyuan, 333, Taiwan.

[d] Department of Orthopaedic Surgery, New Taipei Municipal TuCheng Hospital, Chang Gung Memorial Hospital, Taoyuan, 333, Taiwan.
*Email: blou@mail.cgu.edu.tw

[e] Electrochemical Power Sources Division, CSIR-Central Electrochemical Research Institute, Karaikudi, 630003, Tamil Nadu, India.

[f] Academy of Scientific and Innovative Research (AcSIR), Ghaziabad, 201002, India.

[g] School of Science, RMIT University, Melbourne, Victoria 3001, Australia.

[h] ARC Centre of Excellence in Future Low-Energy Electronics Technologies (FLEET), RMIT University, Melbourne, Victoria 3001, Australia.

[i] Department of Physics, Tamkang University, Tamsui City, 251, Taiwan.
*Email: cldong@mail.tku.edu.tw
[j] National Synchrotron Radiation Research Center, Hsinchu, 30076 Taiwan.

[k] Center for Plasma and Thin Film Technologies, Ming Chi University of Technology, New Taipei, 243, Taiwan.

[l] Department of Marine Environmental Engineering, National Kaohsiung Univeristy of Science and Technology, Kaohsiung City 81157, Taiwan
*Email: twchenchem@gmail.com

Entry	Table of Contents	Page No.
**S1**	Experimental Section	7
**S2**	Calculation	8
**Figure S1.**	(a) Effect of temperature in the preparation of SrCoNi@W_xC is analyzed with XRD pattern of SrCoNi@W_xC -900, SrCoNi@W_xC -800, and SrCoNi@W_xC -700. (b) In order to evaluate the effect of bimetallic atoms on W_xC, the XRD patterns of SrCo@W_xC -900, SrNi@W_xC -900, and CoNi@W_xC -900 are compared. (c) Effect of single metal atom on W_xC, the XRD patterns of Sr@W_xC -900, Co@W_xC -900, and Ni@W_xC -900.	13
**Figure S2.**	(a,b) BET adsorption-desorption hysteresis loop of SrCoNi@W_xC -900 and the calculated pore -diameter distribution curve respectively.	14
**Figure S3**	(a-c) FE-SEM images of SrCoNi@W_xC-800 with various magnifications. (d) Overall elemental mapping images of SrCoNi@W_xC-800 along with the individual elemental mapping of (e) Sr, (f) Co, (g) Ni, (h) W & (i) C-elements. (j,k) BET adsorption-desorption hysteresis loop of SrCoNi@W_xC-800 and the calculated pore diameter distribution curve respectively.	15
**Figure S4**	(a-c) FE-SEM images of SrCoNi@W_xC-700 with various magnifications. (d) Overall elemental mapping images of SrCoNi@W_xC-800 along with the individual elemental mapping of (e) Sr, (f) Co, (g) Ni, (h) W & (i) C-elements. (j,k) BET adsorption-desorption hysteresis loop of SrCoNi@W_xC-700 and the calculated pore diameter distribution curve respectively.	16
**Figure S5**	(a-c) FE-SEM images of CoNi@W_xC-900 with various magnifications. (d) Overall elemental mapping images of CoNi@W_xC-900 along with the individual elemental mapping of (e) Ni, (f) Co, (g) W & (h) C-elements. (i,j) BET adsorption-desorption hysteresis loop of CoNi@W_xC-900 and the calculated pore diameter distribution curve respectively.	17
**Figure S6**	(a-c) FE-SEM images of SrCo@W_xC-900 with various magnifications. (d) Overall elemental mapping images of SrCo@W_xC-900 along with the individual elemental mapping of (e) Sr, (f) Co, (g) W, & (h) C-elements. (I,j) BET adsorption-desorption hysteresis loop of SrCo@W_xC-900 and the calculated pore diameter distribution curve respectively.	18

**Figure S7**	(a-c) FE-SEM images of SrNi@W_xC-900 with various magnifications. (d) Overall elemental mapping images of SrNi@W_xC-900 along with the individual elemental mapping of (e) Sr, (f) Ni, (g) W & (h) C-elements. (I,j) BET adsorption-desorption hysteresis loop of SrNi@W_xC-900 and the calculated pore diameter distribution curve respectively.	19
**Figure S8**	(a,b) FE-SEM images of Sr@W_xC-900 with different magnifications. (c) Overall elemental mapping images of Sr@W_xC-900 along with the individual elemental mapping of (d) Sr, (e) W & (f) C-elements. (g,h) BET adsorption-desorption hysteresis loop of Sr@W_xC-900 and the calculated pore diameter distribution curve respectively.	20
**Figure S9**	(a,b) FE-SEM images of Co@W_xC-900 with different magnifications. (c) Overall elemental mapping images of Co@W_xC-900 along with the individual elemental mapping of (d) Sr, (e) W & (f) C-elements. (g,h) BET adsorption-desorption hysteresis loop of Co@W_xC-900 and the calculated pore diameter distribution curve respectively.	21
**Figure S10**	(a,b) FE-SEM images of Ni@W_xC-900 with different magnifications. (c) Overall elemental mapping images of Ni@W_xC-900 along with the individual elemental mapping of (d) Ni, (e) W & (f) C-elements. (g,h) BET adsorption-desorption hysteresis loop of Ni@W_xC-900 and the calculated pore diameter distribution curve respectively.	22
**Figure S11**	(a-c) FE-SEM images of W_xC-900 with different magnifications. (d) Overall elemental mapping images of W_xC-900 along with the individual elemental mapping of (e) W & (f) C-elements. (g,h) BET adsorption-desorption hysteresis loop of W_xC-900 and the calculated pore diameter distribution curve respectively.	23
**Figure S12**	(a) D- & G- bands of raman spectra, (b) XPS survey spectrum for the (i) SrCoNi@W_xC-900, (ii) SrCoNi@W_xC-800, (iii) SrCoNi@W_xC-700, (iv) SrCo@W_xC-900, (v) SrNi@W_xC-900, (vi) CoNi@W_xC-900, (vii) Sr@W_xC-900, (viii) Co@W_xC-900, (ix) Ni@W_xC-900, and (x) W_xC-900.	24
**Figure S13**	Wavelet Transform images of (a) Co-foil, (b) CoO, (c) CoNi@W_xC-900, & (d) SrCoNi@W_xC-900 and (e) Fourier transformed $k^3\chi$ data of the EXAFS oscillations of Co k-space.	25
**Figure S14**	Wavelet Transform images of (a) Ni-foil, (b) NiO, (c) $Ni(OH)_2$, (d) CoNi@W_xC-900, & (e) SrCoNi@W_xC-900 and (f) Fourier transformed $k^3\chi$ data of the EXAFS oscillations of Ni k-space.	26

**Figure S15**	Wavelet Transform images of (a) WO_3, (b) W_xC-900, (c) CoNi@W_xC-900, & (d) SrCoNi@W_xC-900 and (e) Fourier transformed $k^3\chi$ data of the EXAFS oscillations of W k-space.	27
**Figure S16**	EXAFS of Ni and Co R-space of SrCoNi@WxC.	28
**Figure S17**	(a) OER polarization curve, and (b) tafel slope for Sr@W_xC-900, Co@W_xC-900, Ni@W_xC-900, and W_xC-900 is performed without iR correction using 1 M KOH with the scan rate of 5 mV.	29
**Figure S18**	(a) OER polarization curve, and (b) tafel slope for Sr_{33}CoNi@W_xC-900, Sr_{66}CoNi@W_xC-900, Sr_{99}CoNi@W_xC-900, and Sr_{132}CoNi@W_xC-900 is performed without the iR-correction using 1 M KOH with the scan rate of 5 mV.	30
**Figure S19**	Comparison of TOF for OER performance of SrCoNi@W_xC-900, CoNi@W_xC-900, and W_xC-900.	31
**Figure S20**	CV response of (a) SrCoNi@W_xC-900, (b) SrCoNi@W_xC-800, (c) SrCoNi@W_xC-700, (d) SrCo@W_xC-900, (e) SrNi@W_xC-900, and (f) CoNi@W_xC-900 is observed after the OER performance in the non-faradiac region from 0.824 to 1.024 V vs. RHE in 1 M KOH with the scan rate from 5 to 100 mVs^{-1}.	32
**Figure S21**	OER polarization curve of SrCoNi@W_xC-900 is performed before and after the chronopotentiometry test for 60 hours without the iR-correction using 1 M KOH with the scan rate of 5 mV.	33
**Figure S22**	(a,b) TEM and (c,d) FE-SEM images of SrCoNi@W_xC-900 after the OER stability performance.	34
**Figure S23**	(a) HAADF-STEM image of SrCoNi@W_xC-900 after the OER stability performance and (b-f) elemental mapping acquired over SrCoNi@W_xC-900 for Sr, Co, Ni, W, and C - elements respectively.	35
**Figure S24**	(a) OER stability performance of SrCoNi@WxC-900 at 200 mA cm-2 for 60 hour. (b) EIS spectra of SrCoNi@WxC-900 at catalytic turn over region before and after the stability test.	35
**Figure S25**	(a) HER polarization curve, (b) tafel slope for Sr@W_xC-900, Co@W_xC-900, Ni@W_xC-900, and W_xC-900 is performed without iR correction using 1 M KOH with the scan rate of 5 mV.	36
**Figure S26**	CV response of (a) SrCoNi@W_xC-900, (b) SrCoNi@W_xC-800, (c) SrCoNi@W_xC-700, (d) SrCo@W_xC-900, (e) SrNi@W_xC-900, and (f) CoNi@W_xC -900 is observed after the HER performance in the non-faradiac region from 0.824 to 1.024 V vs. RHE in 1 M KOH with the scan rate from 5 to 100 mVs^{-1}.	37
**Figure S27**	HER polarization curve of Sr_{33}CoNi@W_xC-900, Sr_{66}CoNi@W_xC-900, Sr_{99}CoNi@W_xC-900, and Sr_{132}CoNi@W_xC-900 is	38

	performed without the iR-correction using 1 M KOH with the scan rate of 5 mV.	
**Figure S28**	Comparison of TOF for HER performance of SrCoNi@WxC-900, CoNi@WxC-900, and WxC-900.	39
**Figure S29**	The HER polarization curve of SrCoNi@WxC-900 is performed before and after the chronopotentiometry test for 60 hours without the iR-correction using 1 M KOH with the scan rate of 5 mV.	40
**Figure S30**	(a,b) TEM and (c,d) FE-SEM images of SrCoNi@WxC-900 after the HER stability performance.	41
**Figure S31**	(a) HAADF-STEM image of SrCoNi@WxC-900 after the HER stability performance and (b-f) elemental mapping acquired over SrCoNi@WxC-900 for Sr, Co, Ni, W, and C - elements respectively.	42
**Figure S32**	(a) HER stability performance of SrCoNi@WxC-900 at 200 mA cm-2 for 60 hour. (b) EIS spectra of SrCoNi@WxC-900 at catalytic turn over region before and after the stability test.	42
**Figure S33**	(a) Overall water splitting performance of SrCoNi@WxC-900 in H-cell with 1 M KOH electrolyte separated with the Nafion-117 membrane. (b) Digital photographic image of two electrode performance with 1.5 V battery. (c) Stability performance of SrCoNi@WxC-900 with various current density up to 60 hours.	43
**Figure S34**	The optimized structures of the 4x4 supercells, showing top and side views of two compounds: (a) WxC and (b) CoNi monolayer. Color code- W: pink, C: blue, Cu: orange, and Ni: silver.	44
**Figure S35**	Different configurations of the Sr-doped heterostructure. (a) Sr-cluster (b) Sr-pair and (c) Sr-discrete. Color code - Sr: green.	45
**Table S1**	List of comparison of BJH surface area, pore volume and average pore diameter of the prepared samples.	46
**Table S2**	Atomic weight percentage of each elements calculated from the XPS data.	47
**Table S3**	Atomic weight percentage of each elements calculated from the ICP-MS data.	48
**Table S4**	EXAFS parameters from the fitting.	49
**Table S5**	Comparison of OER activity of SrCoNi@WxC-900 with control samples.	50
**Table S6**	Electrochemical active surface area (ECSA) and double layer capacitance analysis of electrocatalyst performed after the OER performance at the non-faradiac region.	51

**Table S6**	Electrochemical impedance Spectroscopy analysis of electrocatalyst performed after the OER performance with the η@10 mA cm^{-2}.	52
**Table S7**	Comparison of OER activity of SrCoNi@W_xC-900 with the reported literature.	53
**Table S8**	Comparison of HER activity of SrCoNi@W_xC-900 with control catalyst.	54
**Table S9**	Electrochemical active surface area (ECSA) analysis of electrocatalyst performed after the HER performance at the non-faradiac region.	55
**Table S10**	Electrochemical impedance Spectroscopy analysis of electrocatalyst performed after the HER performance with the η@10 mA cm^{-2}.	56
**Table S11**	Comparison of HER activity of SrCoNi@W_xC-900 with the reported literature.	57
**Table S12**	Comparison of overall water splitting activity of SrCoNi@W_xC-900 with the reported literature.	58
**Table S13**	The calculated formation energy of different Sr-doped heterostructures.	59
**Table S14**	Calculated properties of the Sr-doped and undoped heterostructure, including the total magnetic moment (M), bond lengths, interlayer distance (d) and binding energy (Eb).	59

S1. Experimental Section

S1.1 Physicochemical characterization

The phase analysis of the synthesized samples was conducted using the PANalytical X'Pert Pro with Cu Kα radiation (λ= 1.54Å), the obtained XRD patterns were then compared with the JCPDS number using X'pert HighScore Plus software. The active vibrational modes of the prepared samples were analyzed with the Micro-Raman Spectrometer (Raman Dong Woo 500 I, Korea). X-ray photoelectron spectroscopy was performed on a Thermo ESCLAB with the Al kα radiation to analyze the valance state of the metal atoms. The elemental weight (%) of constitutional elements in the synthesized samples was analyzed with the NexION ICP-MS instrument under the standard range of 0.1 ppm to 10 ppm. Surface area measurement and Barrett–Joyner–Halenda (BJH) pore volume distribution analysis were carried out using the Tristar II plus instrument. Thermal stability of the synthesized samples was conducted using NETZSCH TG-209 instrument under N_2 atmosphere. For morphological analysis and elemental distribution, the JEOL JSM-7610F was utilized to capture field emission scanning electron microscopic images with an accelerating voltage of 20 kV, coupled with the energy dispersive spectroscopy. High-resolution transmission electron microscope images and elemental mappings were acquired using the Shimadzu JEM-1200 EX, coupled with energy dispersive x-ray spectroscopy. X-ray absorption spectroscopy (XAS) was performed at the Taiwan Light Source (TLS) beamline BL17C, equipped with a Si (111) double crystal monochromator, and the LS storage ring operated with 1.5 GeV with a current of 365 mA at the National Synchrotron Radiation Research Center (NSRRC), Taiwan. XAS spectra were recorded in the transmission mode, followed by data were interpretation using standard protocol for pre-and post-edge subtractions, edge jump, and Fourier transformation.

S1.2 Electrochemical performance

The electrochemical performance for the OER and HER was evaluated using a three-electrode set up in a 100 mL beaker and the OWS was performed using an H-cell with nafion membrane containing 1 M KOH as electrolyte at room temperature. Nickel foam (NF) was employed as conducting substrate of working electrode. To prepare working electrode, a piece of NF (1×1 cm^{-2}) was cleaned by ultrasonication using 1M HCl, water, ethanol for 5 minutes respectively, and allowed to dry in air oven at 65 °C. The cleaned NF is loaded with 500 µL of catalyst ink (5 mg catalyst + 475 µL isopropyl alcohol

+ 25 µL Nafion) by drop casting method, allowed to dry in air oven at 65 °C for overnight and the dried electrode was used as working electrode. The Hg/HgO electrode was used as the reference electrode, its potential was converted to the reversible hydrogen electrode (RHE) potential (E = E_o + 0.098 + 0.059 × pH) and the platinum wire was used as the counter electrode. The electrochemical polarization curves (CV & LSV), stability performances (chronopotentiometry) are obtained from CHI627A electrochemical analyzer (CHI instrument, USA). The electrochemical impedance spectroscopy was performed using Admiral Squidstat Plus from the frequency range of 1 KHz to 0.1 Hz with 5 mV amplitude.

S2. Calculation

S2.1 Electrochemical active surface area (ECSA)

The electrochemical active surface area of electrode is calculated from the CV response obtained from the non-faradic region (0.832 – 1.016 V vs. RHE) performed after the OER and HER performances.

$$ECSA = R_f\ S$$

$$R_f = \frac{C_{dl}}{C_s} \quad (1)$$

Where C_s is the specific capacitance (for NF: 0.040 mF cm^{-2}) and S is the area of the working electrode.

S2.2 Turnover frequency (TOF)

The turnover frequency per active site for the electrocatalysis performance is calculated with following equation,

$$TOF = \frac{number\ of\ oxygen\ or\ hydrogen\ turnovers\ per\ cm^2\ of\ geometric\ area}{number\ of\ active\ site\ per\ cm^2\ of\ geometric\ area} \quad (2)$$

The number of oxygen turnovers is calculated using current density with the equation ,

$$no. of\ O_2$$

$$= \left(j\ \frac{mA}{cm^2}\right)\left(\frac{1\ C\ s^{-1}}{1000\ mA}\right)\left(\frac{1\ mol\ of\ e^-}{96485.3\ C}\right)\left(\frac{1\ mol\ of\ H_2}{4\ mol\ of\ e^-}\right)\left(\frac{6.022\times 10^{22}\ O_2\ molecules}{1\ mol\ of\ H_2}\right)$$

$$= 1.56\ \times\ 10^{15}\ \frac{O_2/s}{cm^2}\ mA^{-1}\ cm^2 \qquad (3)$$

The number of hydrogen turnovers is calculated using current density with the equation ,

$$no.of\ H_2 = \left(j\ \frac{mA}{cm^2}\right)\left(\frac{1\ C\ s^{-1}}{1000\ mA}\right)\left(\frac{1\ mol\ of\ e^-}{96485.3\ C}\right)\left(\frac{1\ mol\ of\ H_2}{2\ mol\ of\ e^-}\right)\left(\frac{6.022\times10^{22}\ H_2\ molecules}{1\ mol\ of\ H_2}\right)$$

$$= 3.12\ \times\ 10^{15}\ \frac{H_2/s}{cm^2}\ mA^{-1}\ cm^2 \qquad (4)$$

the number of atoms present per unit cell of hexagonal system, having the lattice constant of 11.888 Å with the total number of atom is 16 for both the SrCoNi@WxC and CoNi@ WxC used to calculate the active sites per real surface area with the below equation,

$$Active\ sites_{SrCoNi@WC} = \left(\frac{16\ atom\ per\ unit\ cell}{11.888\ Å^3\ per\ unit\ cell}\right)^{2/3}$$

$$= 12.19\times10^{15}\times atom\ cm_{real}^{-2}$$

$$Active\ sites_{CoNi@WC} = \left(\frac{16\ atom\ per\ unit\ cell}{11.888\ Å^3\ per\ unit\ cell}\right)^{2/3}$$

$$= 12.19\times10^{15}\times atom\ cm_{real}^{-2}$$

$$Active\ sites_{WC} = \left(\frac{16\ atom\ per\ unit\ cell}{11.888\ Å^3\ per\ unit\ cell}\right)^{2/3}$$

$$= 12.19\times10^{15}\times atom\ cm_{real}^{-2}$$

$$TOF_{real} = \frac{3.12\ \times\ 10^{15}\ \frac{H_2/s}{cm^2}\ mA^{-1}\ cm^2}{Active\ sites_{real}\times ECSA}\times\left|\,j\,\right| \qquad (5)$$

S2.3 Computational Methodology

To analyze the active site, the Gibbs free energy, and the catalytic mechanism of the SrCoNi@WxC heterostructure were calculated using density functional theory (DFT) calculations. These calculations were conducted using the projected augmented wave (PAW) method as implemented in the Vienna *Ab initio* Simulation package (VASP).(1-3) The Perdew–Burke–Ernzerhof (PBE) functional for the exchange–correlation with a

kinetic energy cutoff of 450 eV was used for all computations in the spin–polarized DFT method.(4) To generate highly accurate results, we include the use of a Hubbard U term. The U-values, which are applied to the d-orbitals of Ni, and Co were 6.20 and 3.32 eV, respectively.(5) We employed Grimme's DFT-D3 correction approach to accurately depict the long-range van der Waals interactions.(6-7) An electronic convergence of 10^{-6} eV was used. The ionic relaxations were carried out without consideration of symmetry. All ions were relaxed until the absolute atomic forces were lower than 0.01eV/Å. K-Points sampling of 3x3x1 was used for all structures. The Bader charge analysis was performed using the method of Henkelmen *et al.*(8)

The four electron transfer steps that involve the overall OER process are represented as (5-8):

$$M^* + OH^- \rightarrow M - OH^* + e^- \quad (5)$$

$$M^* - OH^* + OH^- \rightarrow M - O^* + H_2O + e^- \quad (6)$$

$$M - O^* + OH^- \rightarrow M - OOH^* + e^- \quad (7)$$

$$M - OOH^* + OH^- \rightarrow M^* + O_2 + H_2O + e^- \quad (8)$$

where, * denotes the active site on the catalysts surface, and OH*, O* and OOH* represent the different intermediates species in each step.

The adsorption energy (E_{ad}), of each intermediate were evaluated using the following equations (9-11):

$$\Delta E_{OH^*} = E_{OH^*} - E^* - E_{H_2O} + \frac{1}{2} E_{H_2} \quad (9)$$

$$\Delta E_{O^*} = E_{O^*} - E^* - E_{H_2O} + E_{H_2} \quad (10)$$

$$\Delta E_{OOH^*} = E_{OOH^*} - E^* - 2E_{H_2O} + \frac{3}{2} E_{H_2} \quad (11)$$

Where $E_{OH^*/O^*/OOH^*}$ and E^* are the total energy of the adsorbed surface with three intermediates and the total energy of the pristine heterostructure, respectively. E (H_2O). and E(H_2) are the calculated ground state energies for the H_2O, and H_2 molecules, respectively. With regards to the definition of E_{ad}, negative values indicate the favorable

adsorption site, whereas positive results denote that energy is required to adsorb molecule on the surface.

In this connection, the Gibbs free energy ΔG_n (n=1, 2, 3, 4) values were calculated using the following formula (9):

$$\Delta G_n = \Delta E + \Delta E_{ZPE} - T\Delta S - eU \qquad (12)$$

Here, ΔE stands for adsorption energy of the intermediate. ΔE_{ZPE} and ΔS represents the zero-point energy and entropy contribution, respectively. T is the temperature (298.15 K), and U is the electrode potential.

The theoretical overpotential η^{OER} was determined by (10):

$$G^{OER} = \text{Max}\ \{\Delta G_1, \Delta G_2, \Delta G3, \Delta G_4\} \qquad (13)$$

$$\eta^{OER} = G^{OER}/e - 1.23V \qquad (14)$$

The two-electron process that involves the overall HER is represented as:

$$M + H_2O \rightarrow M - H^* + OH^- \qquad (15)$$

$$M - H^* + H_2O \rightarrow M^* + H_2 + OH^- \qquad (16)$$

The hydrogen adsorption energy (ΔE_{H^*})was calculated using the equation

$$\Delta E_{H^*} = E_{H^*} - E^* - \frac{1}{2} E_{H_2} \qquad (17)$$

Here, E^* *and* E_{H^*} represent the total energies of system before and after H-adsorption. E_{H_2} is the energy of the H_2 molecule in the gas phase.

The Gibbs free energy of the HER is given as,

$$\Delta G_{H^*} = \Delta\ E_{H^*} + \Delta ZPE - T\Delta S \qquad (18)$$

The binding energy (E_b), was calculated as:

$$E_b = - \frac{E_{Total} - \sum(E_{Individual})}{n} \qquad (19)$$

where E_{total} and $E_{individual}$ are the total energies of the entire system (HS) and the individual layers, respectively. n is the number of unit cell.

The Formation energy (E_f) for different configurations of the Sr-doped heterostructure were calculated and evaluate their structural stability, according to the formula $E_f = E_{\mathrm{Sr@HS}} - E_{\mathrm{HS}} - E_{\mathrm{Sr}}$, where $E_{\mathrm{Sr@HS}}$ is the total energy for Sr-doped HS, E_{HS} is the energy for undoped HS and E_{Sr} is the energy for single Sr atom.

The charge transfer and redistribution across the heterostructure were quantitatively analyzed by calculating the charge density difference Δρ, which is expressed by the following formula.

$$\Delta\rho = \rho_{\mathrm{total}} - \rho_{\mathrm{SrCoNi}} - \rho_{\mathrm{WXC}} \quad (20)$$

where, ρ_{total}, ρ_{SrCoNi}, and ρ_{WXC} denote the charge density of the SrCoNi@W_xC heterostructure, the individual SrCoNi system and the W_xC layer, respectively. The VESTA software was utilized to model and visualize the atomic structural models as well as the charge density difference.(11)

S3. Supplementary figures

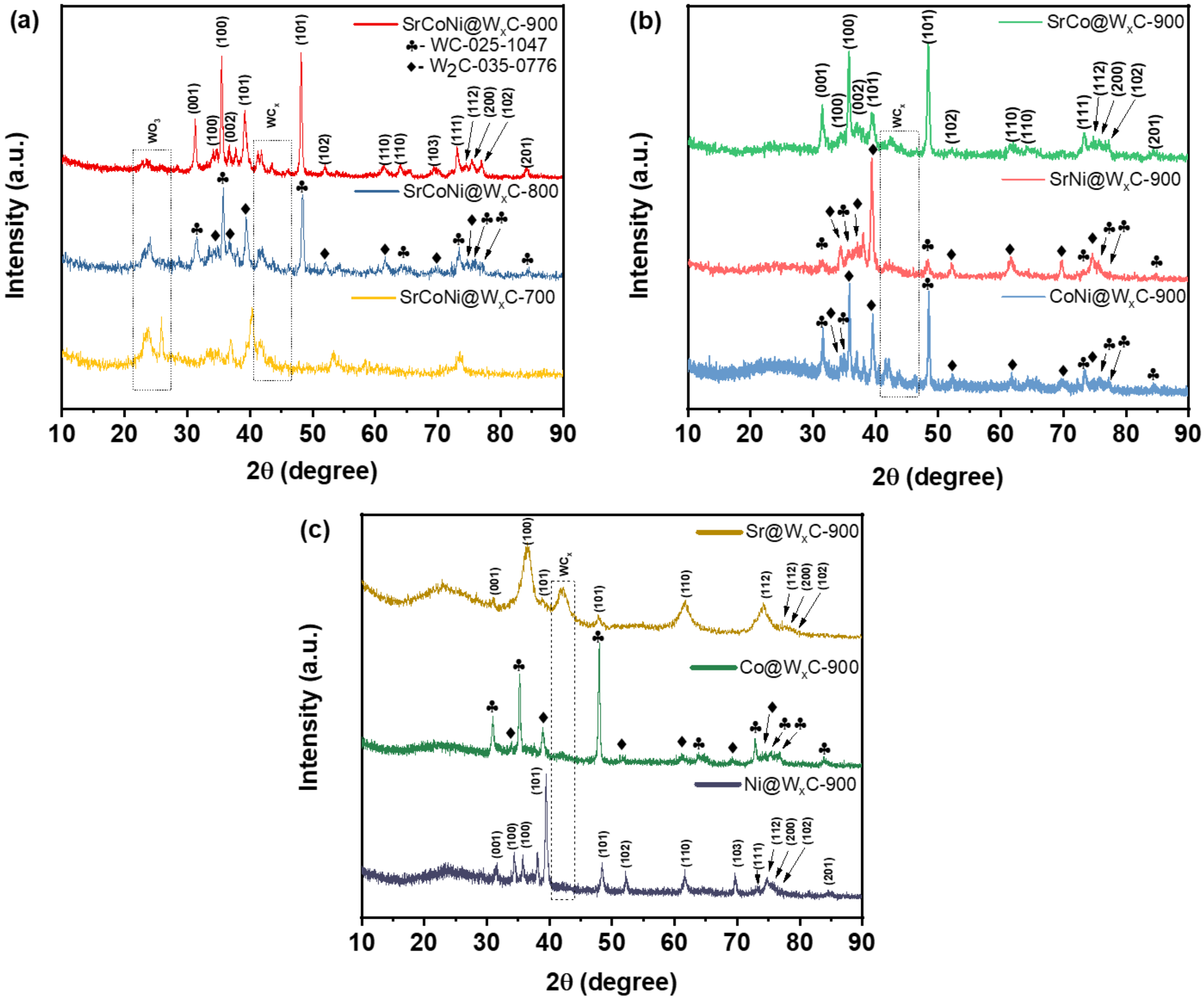

Figure S1. (a) Effect of temperature in the preparation of SrCoNi@W_xC is analyzed with XRD pattern of SrCoNi@W_xC-900, SrCoNi@W_xC-800, and SrCoNi@W_xC-700. (b) In order to evaluate the effect of bimetallic atoms on W_xC, the XRD patterns of SrCo@W_xC-900, SrNi@W_xC-900, and CoNi@W_xC-900 are compared. (c) Effect of single metal atom on W_xC, the XRD patterns of Sr@W_xC-900, Co@W_xC-900, and Ni@W_xC-900.

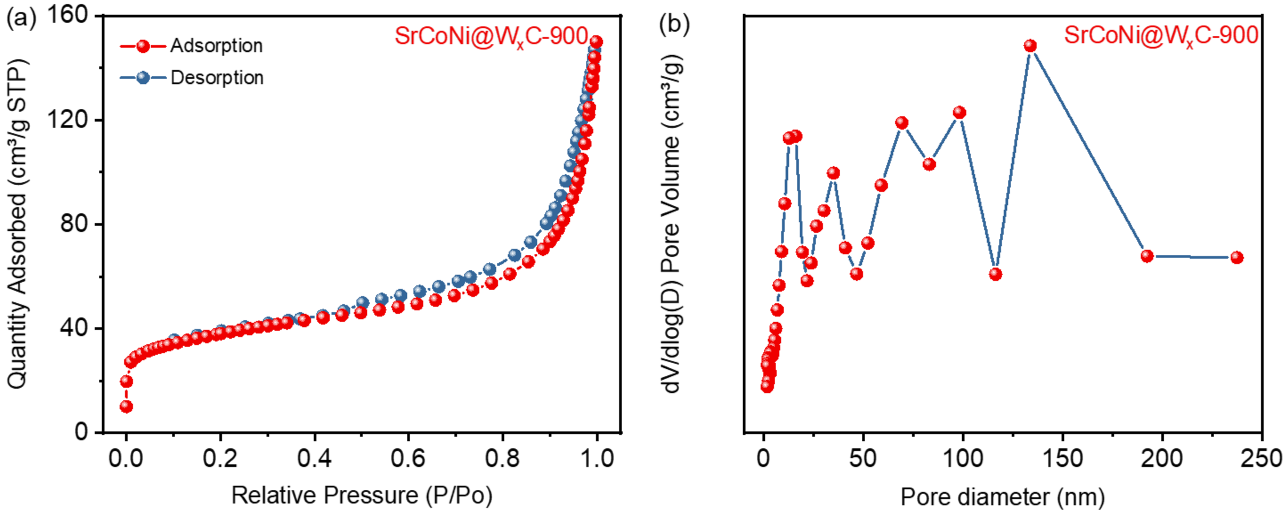

Figure S2. (a,b) BET adsorption-desorption hysteresis loop of SrCoNi@WxC-900 and the calculated pore -diameter distribution curve respectively.

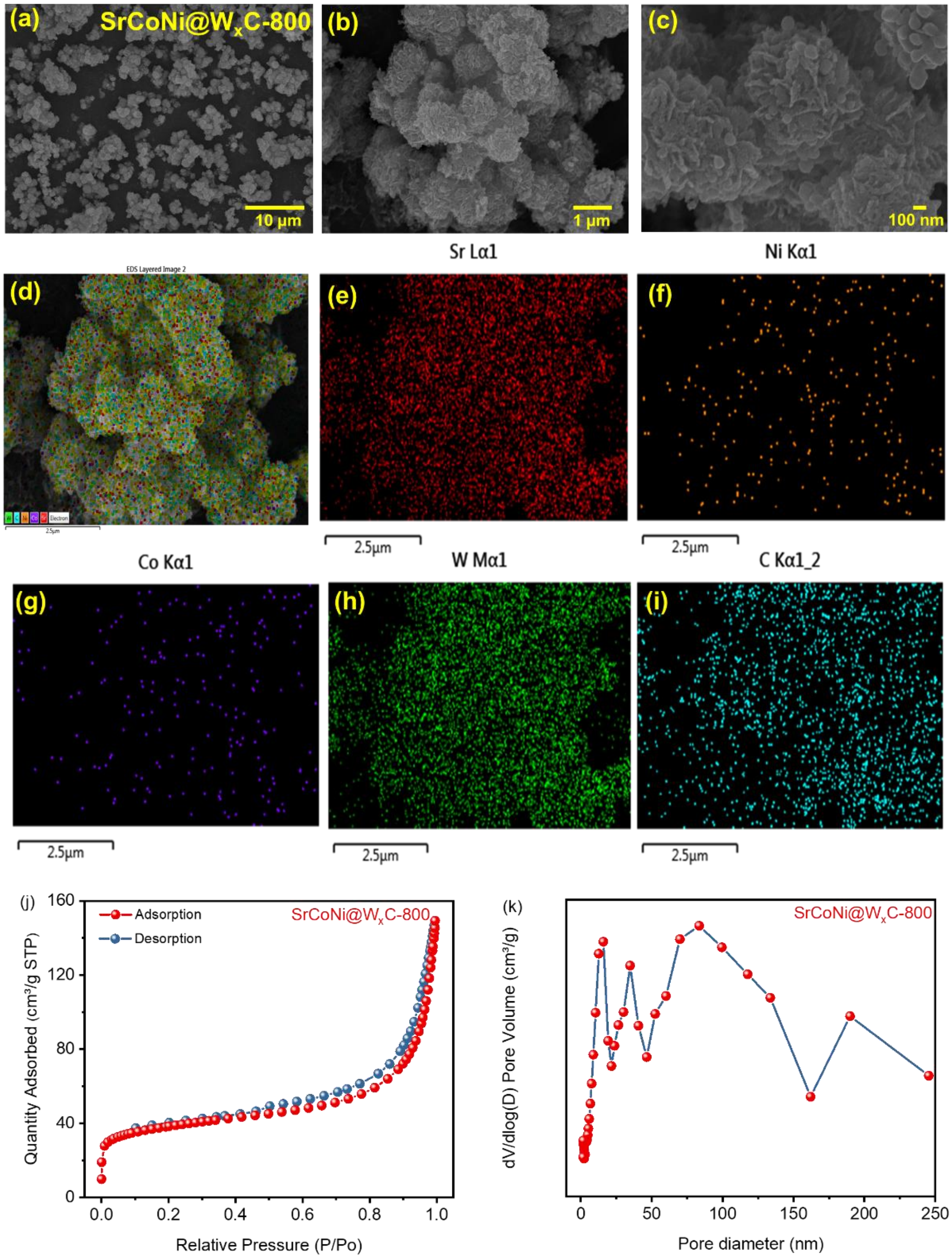

Figure S3. (a-c) FE-SEM images of SrCoNi@WxC-800 with various magnifications. (d) Overall elemental mapping images of SrCoNi@WxC-800 along with the individual elemental mapping of (e) Sr, (f) Co, (g) Ni, (h) W & (i) C-elements. (j,k) BET adsorption-desorption hysteresis loop of SrCoNi@WxC-800 and the calculated pore diameter distribution curve respectively.

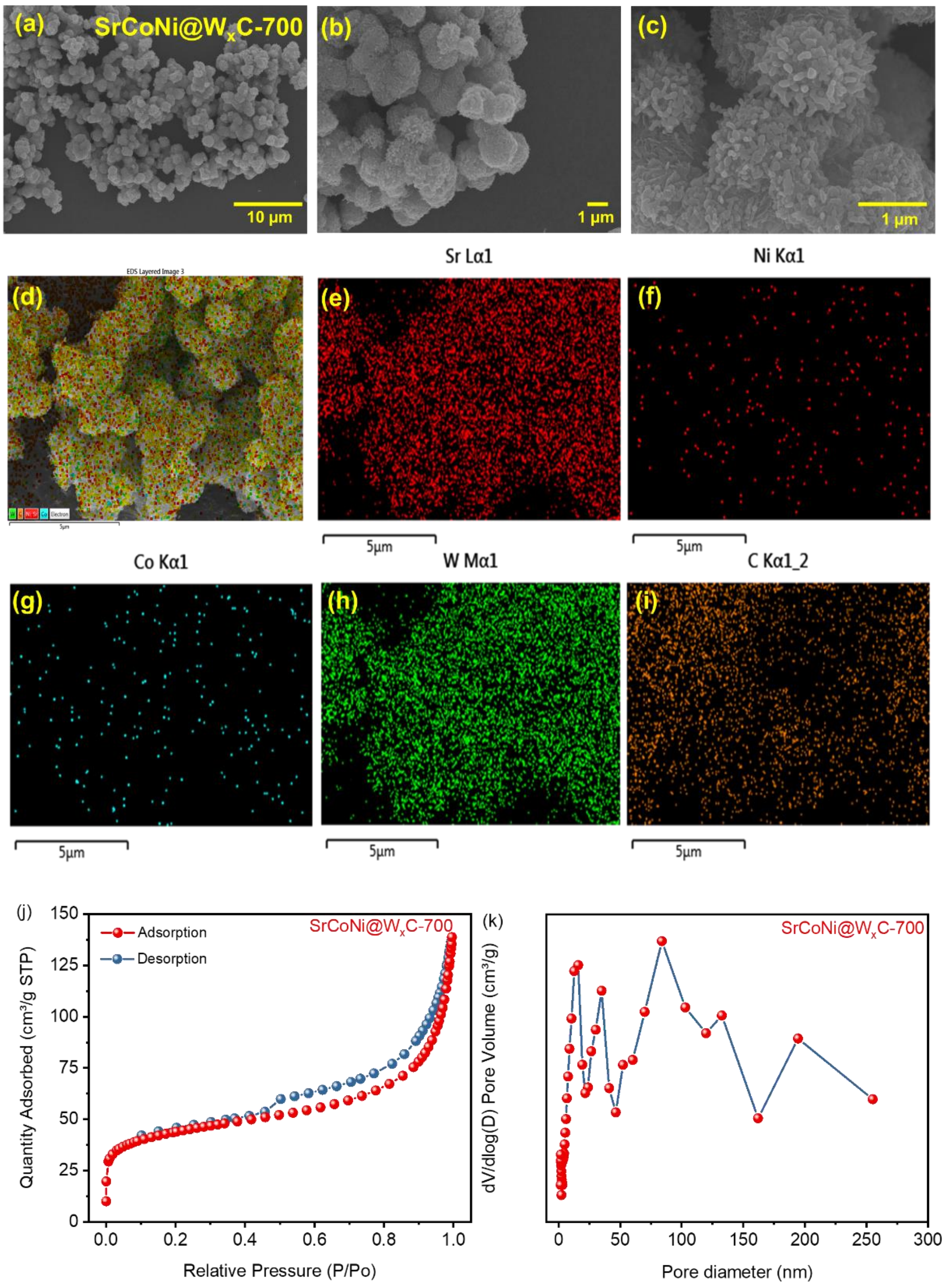

Figure S4. (a-c) FE-SEM images of SrCoNi@WxC-700 with various magnifications. (d) Overall elemental mapping images of SrCoNi@WxC-800 along with the individual elemental mapping of (e) Sr, (f) Co, (g) Ni, (h) W & (i) C-elements. (j,k) BET adsorption-desorption hysteresis loop of SrCoNi@WxC-700 and the calculated pore diameter distribution curve respectively.

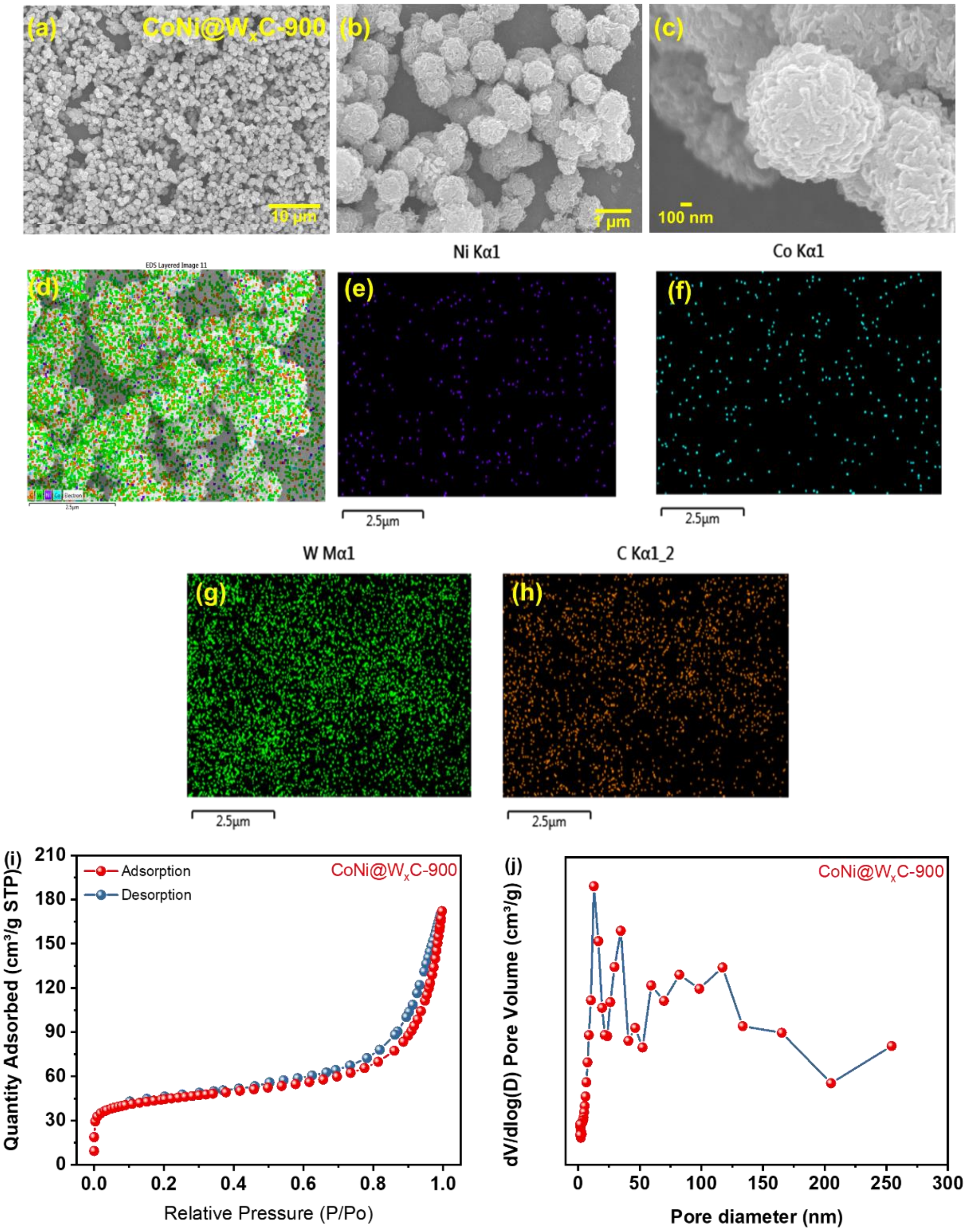

Figure S5. (a-c) FE-SEM images of CoNi@WxC-900 with various magnifications. (d) Overall elemental mapping images of CoNi@WxC-900 along with the individual elemental mapping of (e) Ni, (f) Co, (g) W & (h) C-elements. (i,j) BET adsorption-desorption hysteresis loop of CoNi@WxC-900 and the calculated pore diameter distribution curve respectively.

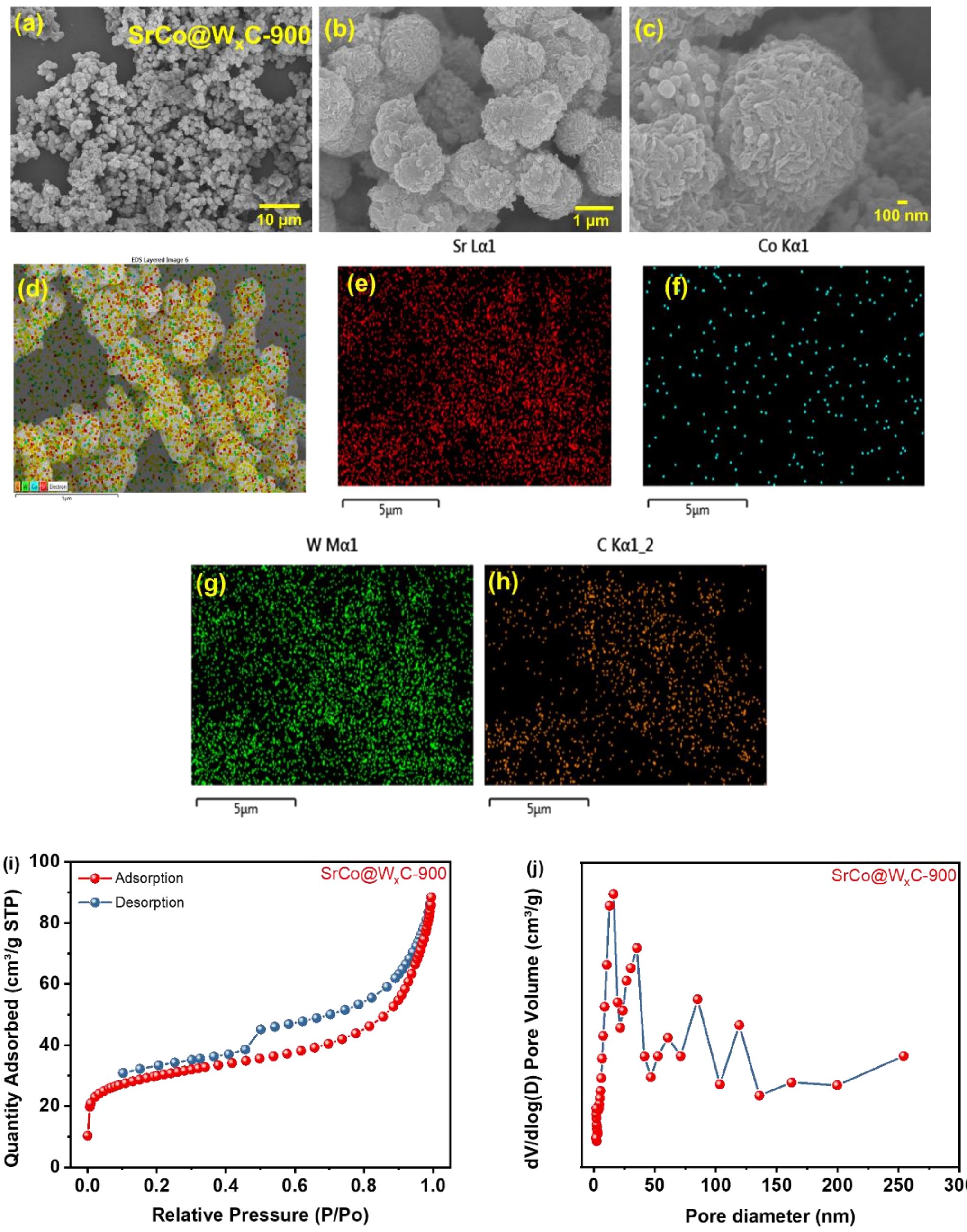

Figure S6. (a-c) FE-SEM images of SrCo@WxC-900 with various magnifications. (d) Overall elemental mapping images of SrCo@WxC-900 along with the individual elemental mapping of (e) Sr, (f) Co, (g) W, & (h) C-elements. (I,j) BET adsorption-desorption hysteresis loop of SrCo@WxC-900 and the calculated pore diameter distribution curve respectively.

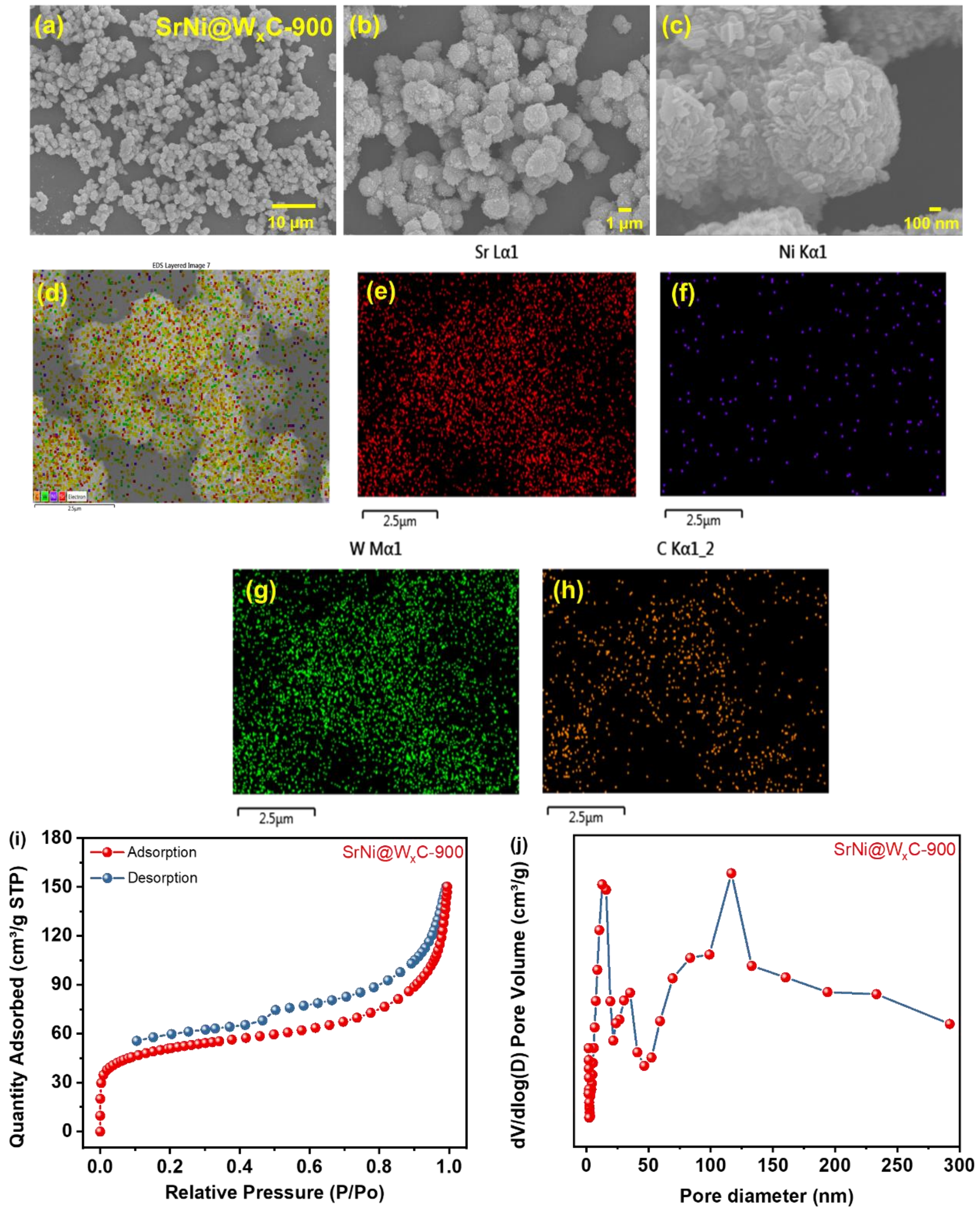

Figure S7. (a-c) FE-SEM images of SrNi@WxC-900 with various magnifications. (d) Overall elemental mapping images of SrNi@WxC-900 along with the individual elemental mapping of (e) Sr, (f) Ni, (g) W & (h) C-elements. (I,j) BET adsorption-desorption hysteresis loop of SrNi@WxC -900 and the calculated pore diameter distribution curve respectively.

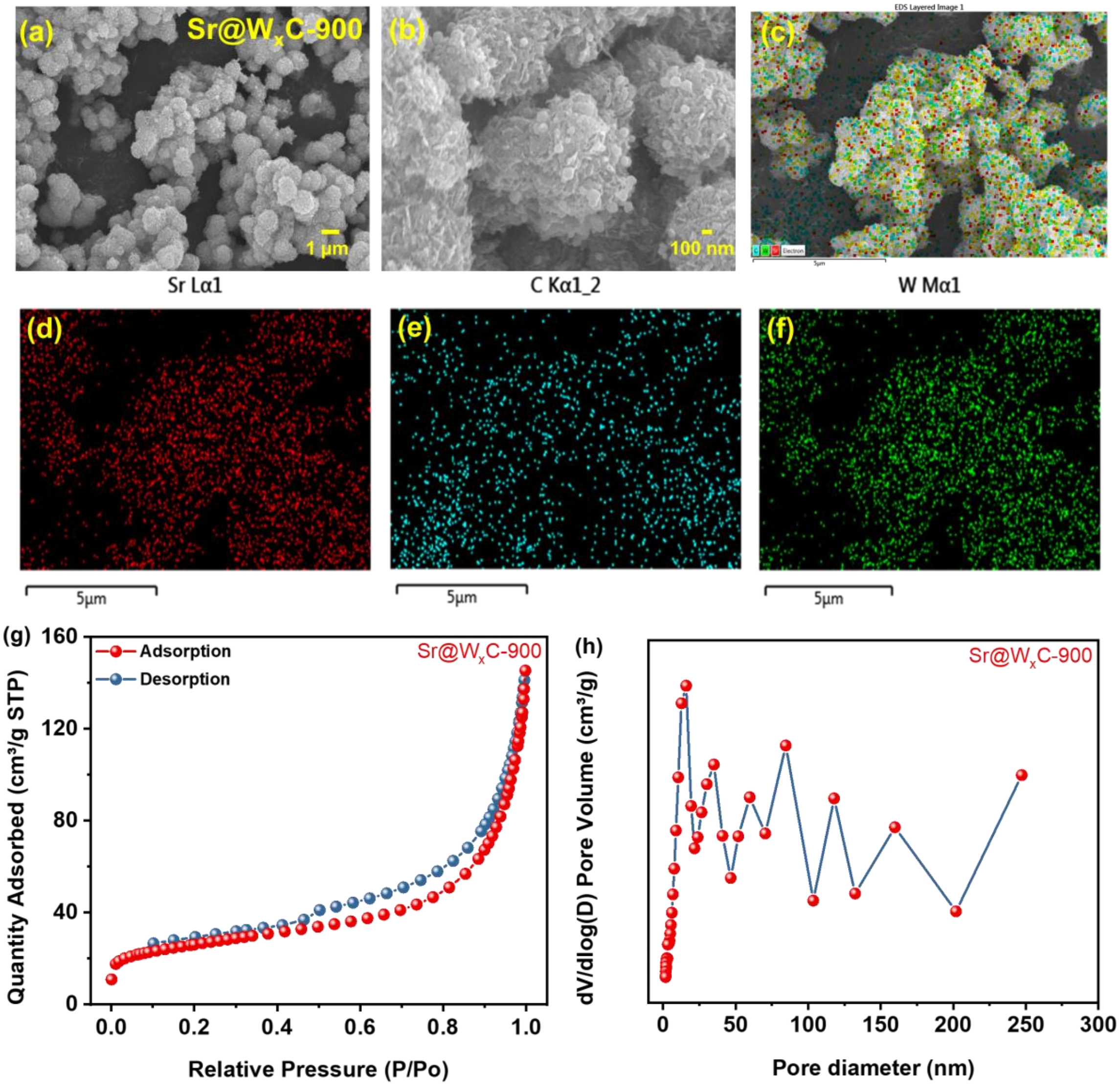

Figure S8. (a,b) FE-SEM images of Sr@WxC-900 with different magnifications. (c) Overall elemental mapping images of Sr@WxC-900 along with the individual elemental mapping of (d) Sr, (e) W & (f) C-elements. (g,h) BET adsorption-desorption hysteresis loop of Sr@WxC-900 and the calculated pore diameter distribution curve respectively.

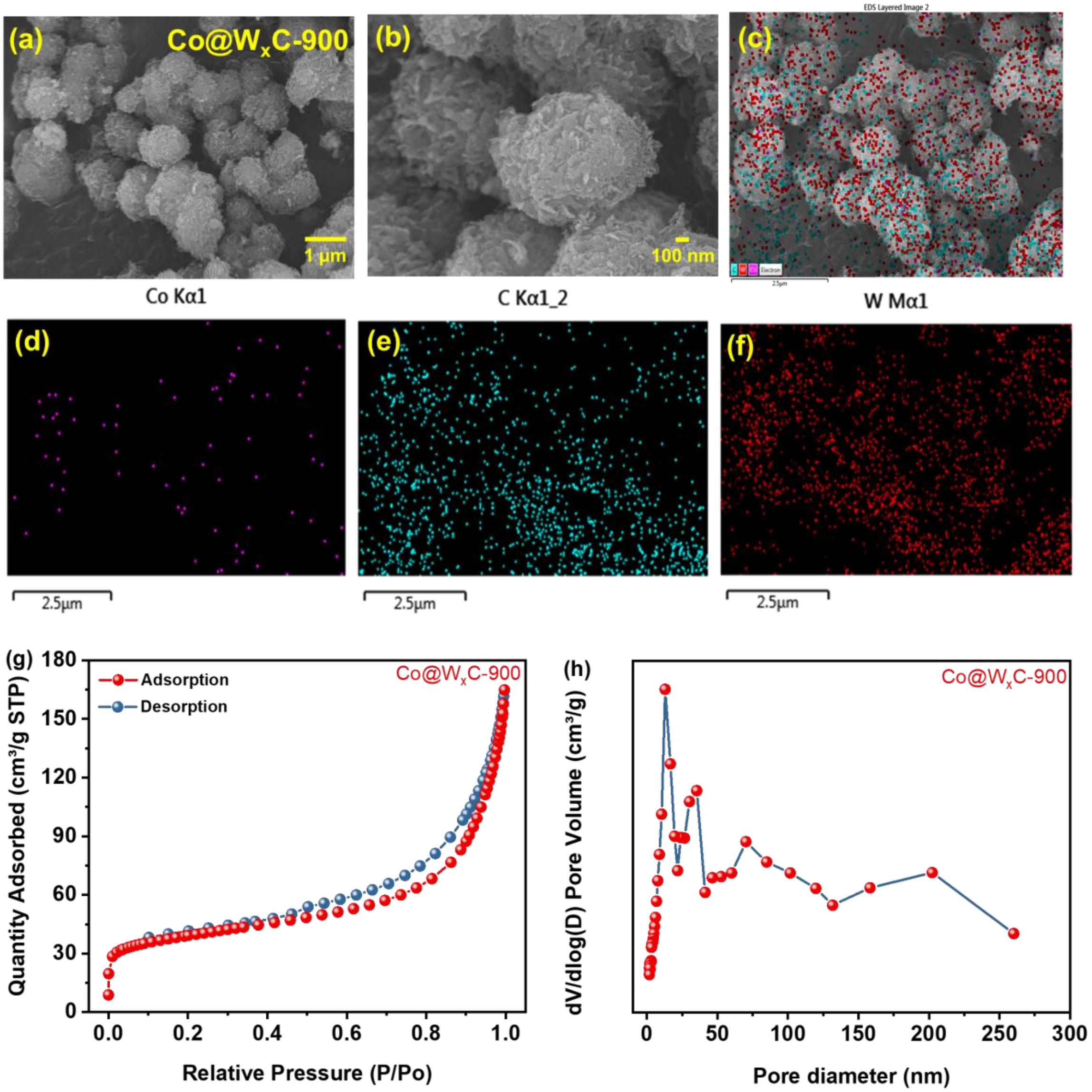

Figure S9. (a,b) FE-SEM images of Co@WxC-900 with different magnifications. (c) Overall elemental mapping images of Co@WxC-900 along with the individual elemental mapping of (d) Sr, (e) W & (f) C-elements. (g,h) BET adsorption-desorption hysteresis loop of Co@WxC-900 and the calculated pore diameter distribution curve respectively.

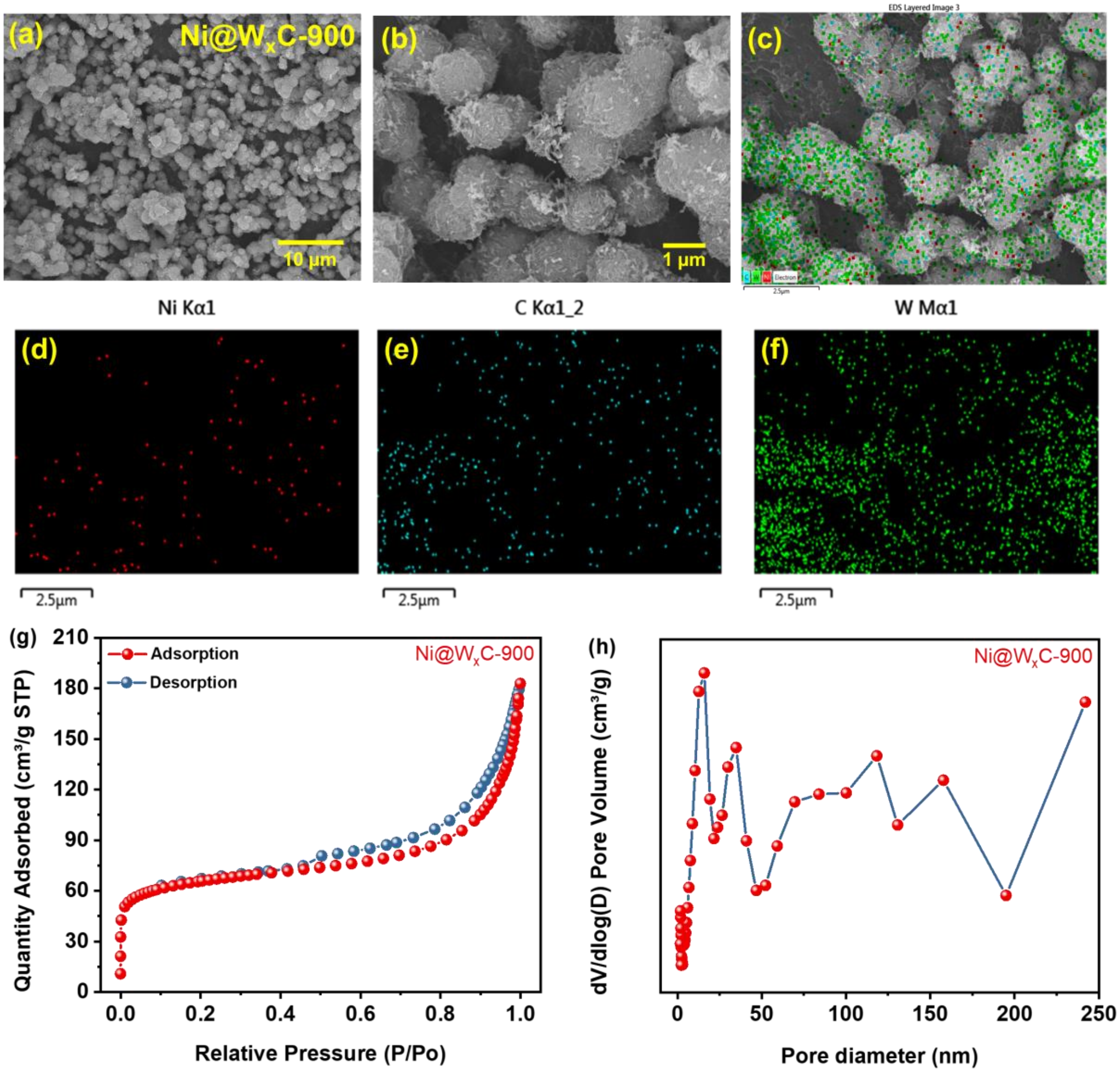

Figure S10. (a,b) FE-SEM images of Ni@WxC-900 with different magnifications. (c) Overall elemental mapping images of Ni@WxC-900 along with the individual elemental mapping of (d) Ni, (e) W & (f) C-elements. (g,h) BET adsorption-desorption hysteresis loop of Ni@WxC-900 and the calculated pore diameter distribution curve respectively.

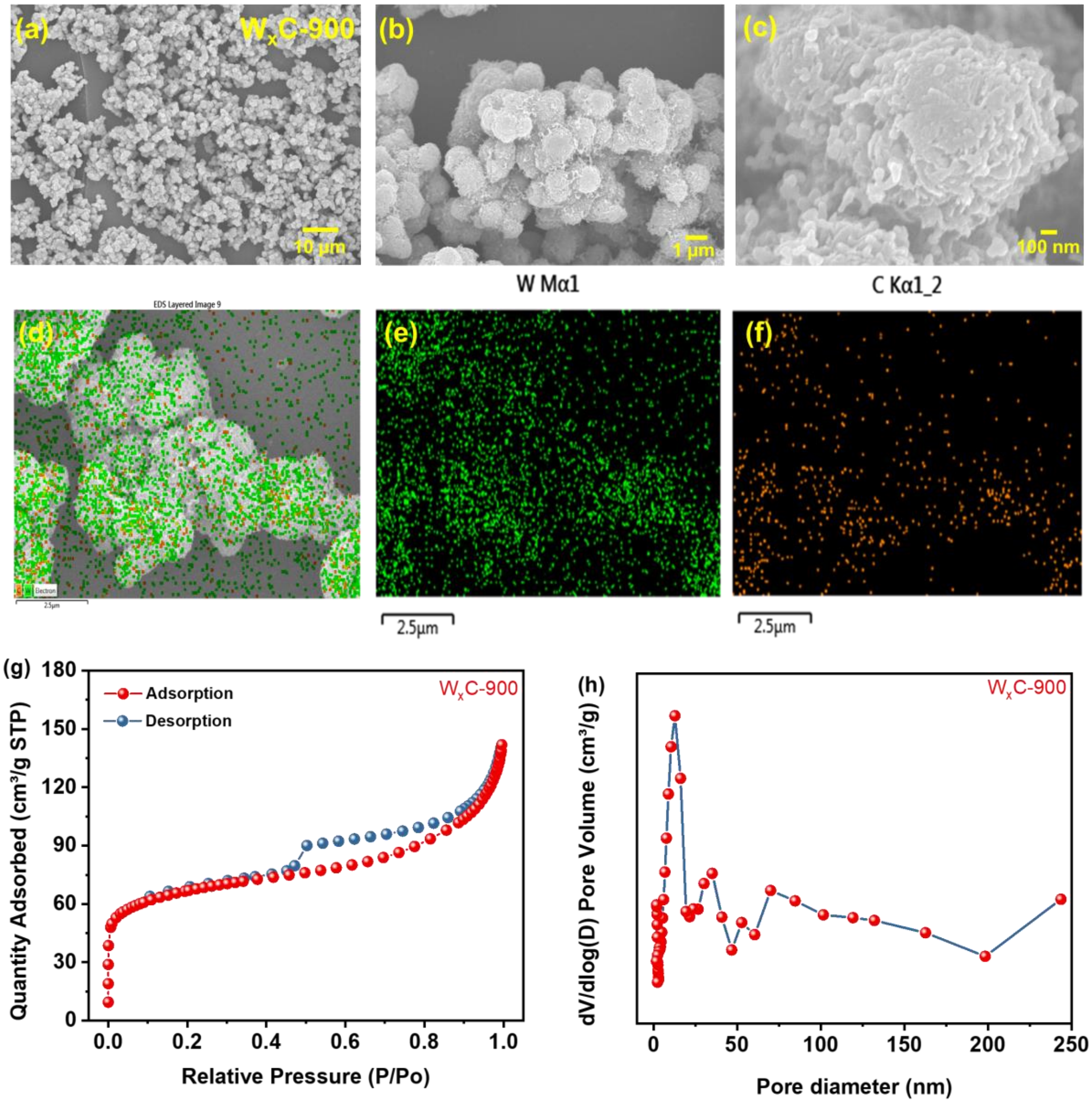

Figure S11. (a-c) FE-SEM images of WxC-900 with different magnifications. (d) Overall elemental mapping images of WxC-900 along with the individual elemental mapping of (e) W & (f) C-elements. (g,h) BET adsorption-desorption hysteresis loop of WxC-900 and the calculated pore diameter distribution curve respectively.

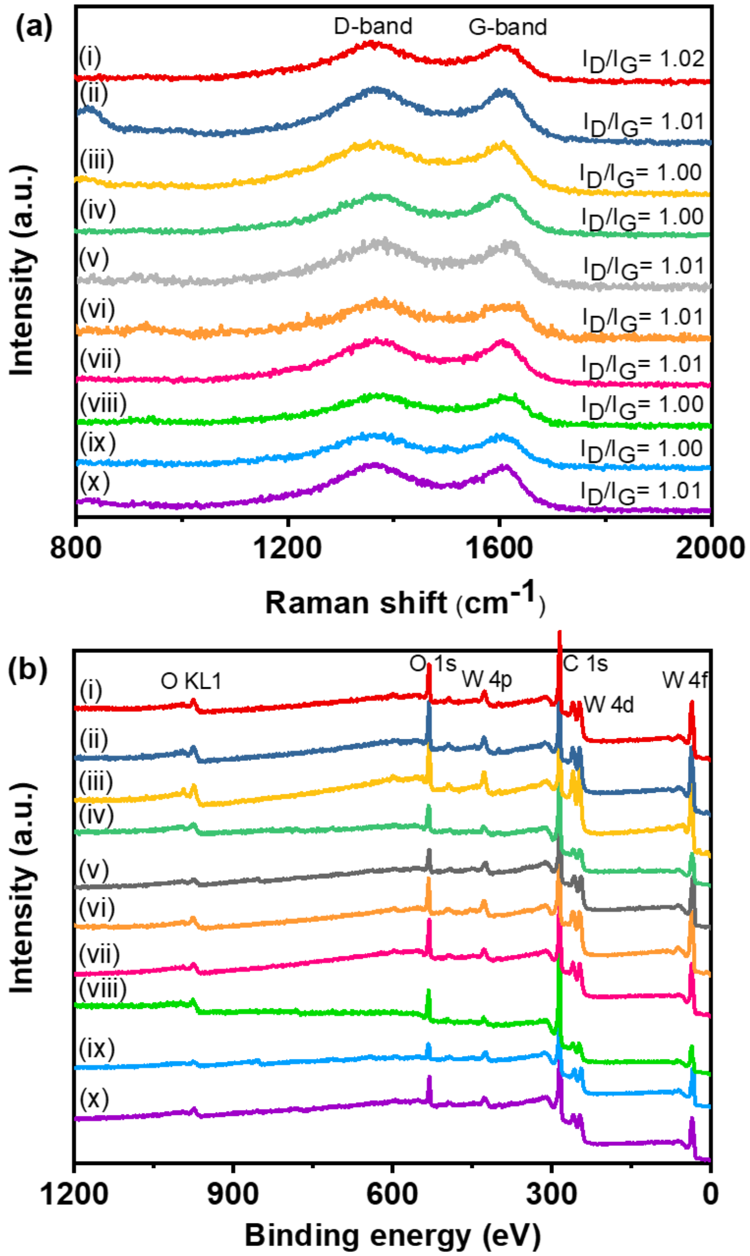

Figure S12. (a) D- & G- bands of raman spectra, (b) XPS survey spectrum for the (i) SrCoNi@W_xC-900, (ii) SrCoNi@W_xC-800, (iii) SrCoNi@W_xC-700, (iv) SrCo@W_xC-900, (v) SrNi@W_xC-900, (vi) CoNi@W_xC-900, (vii) Sr@W_xC-900, (viii) Co@W_xC-900, (ix) Ni@W_xC-900, and (x) W_xC-900.

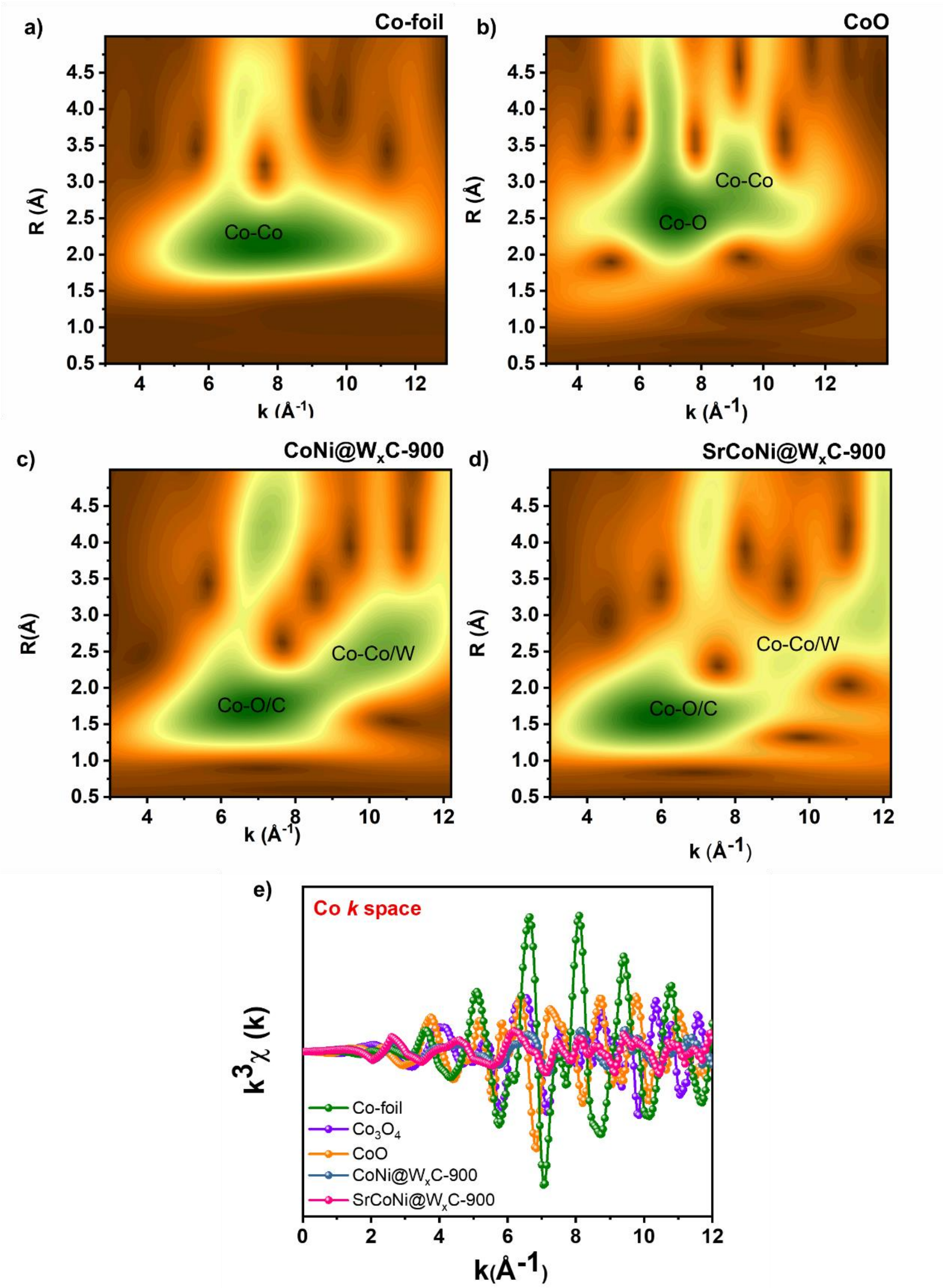

Figure S13. Wavelet Transform images of (a) Co-foil, (b) CoO, (c) CoNi@W_xC-900, & (d) SrCoNi@W_xC-900 and (e) Fourier transformed $k^3\chi$ data of the EXAFS oscillations of Co k-space.

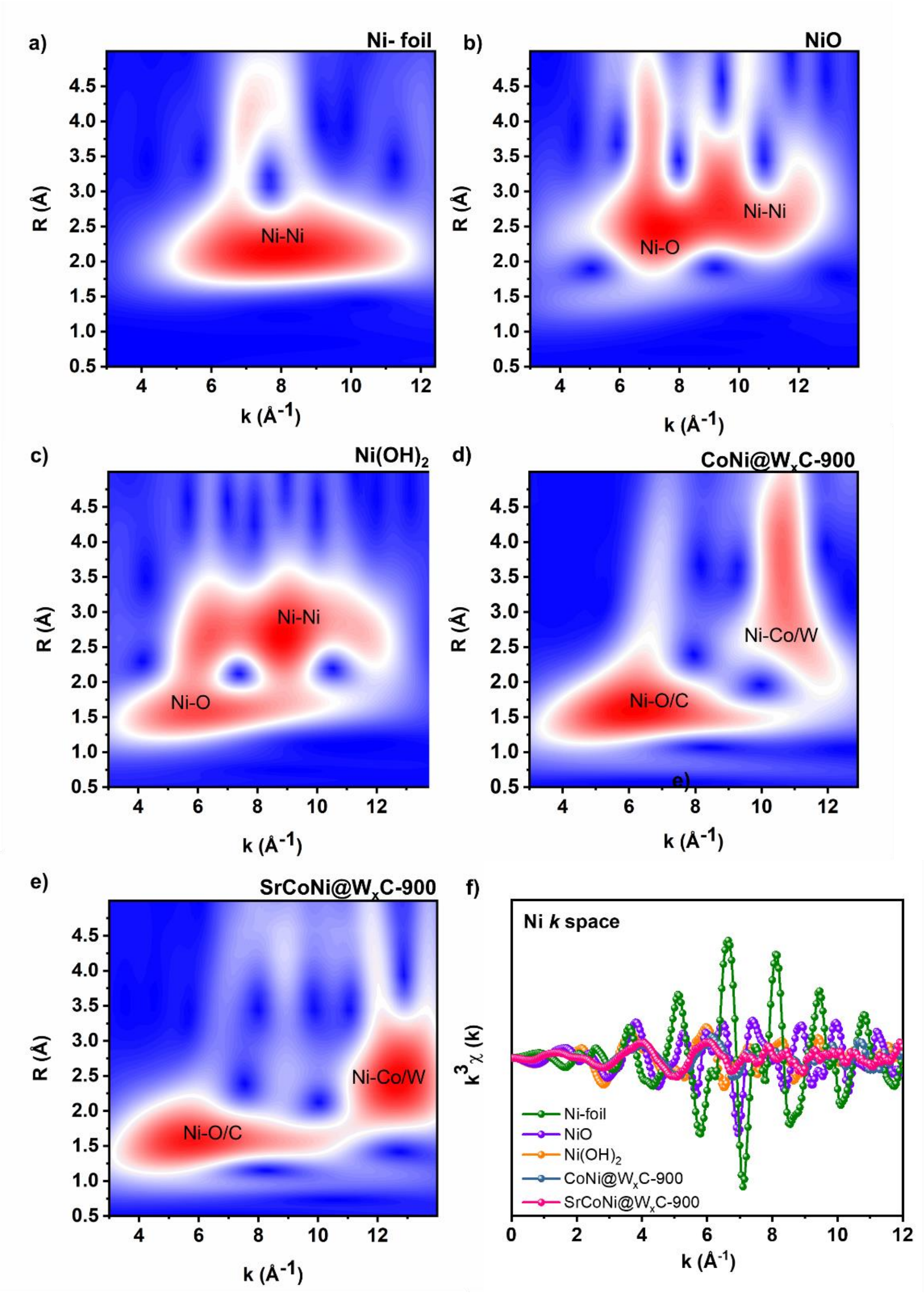

Figure S14. Wavelet Transform images of (a) Ni-foil, (b) NiO, (c) $Ni(OH)_2$, (d) CoNi@W_xC-900, & (e) SrCoNi@W_xC-900 and (f) Fourier transformed $k^3\chi$ data of the EXAFS oscillations of Ni k-space.

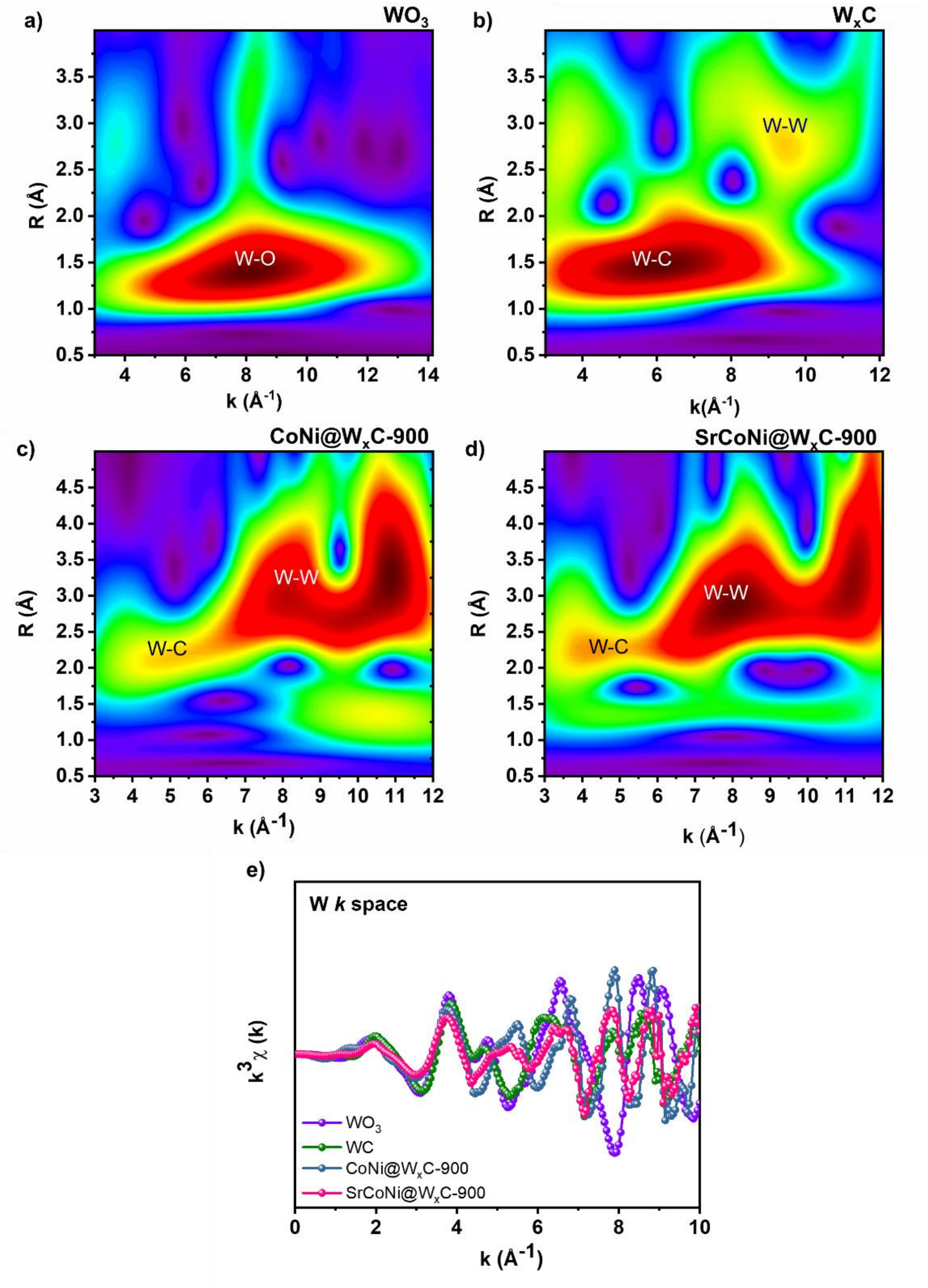

Figure S15. Wavelet Transform images of (a) WO_3, (b) W_xC-900, (c) CoNi@W_xC-900, & (d) SrCoNi@W_xC-900 and (e) Fourier transformed $k^3\chi$ data of the EXAFS oscillations of W k-space.

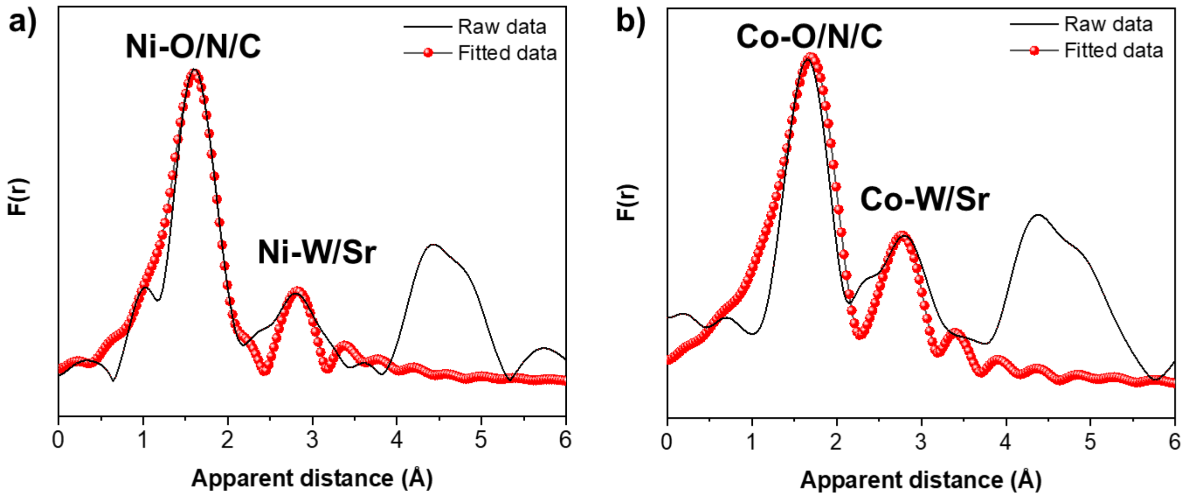

Figure S16. EXAFS of Ni and Co R-space of SrCoNi@WxC.

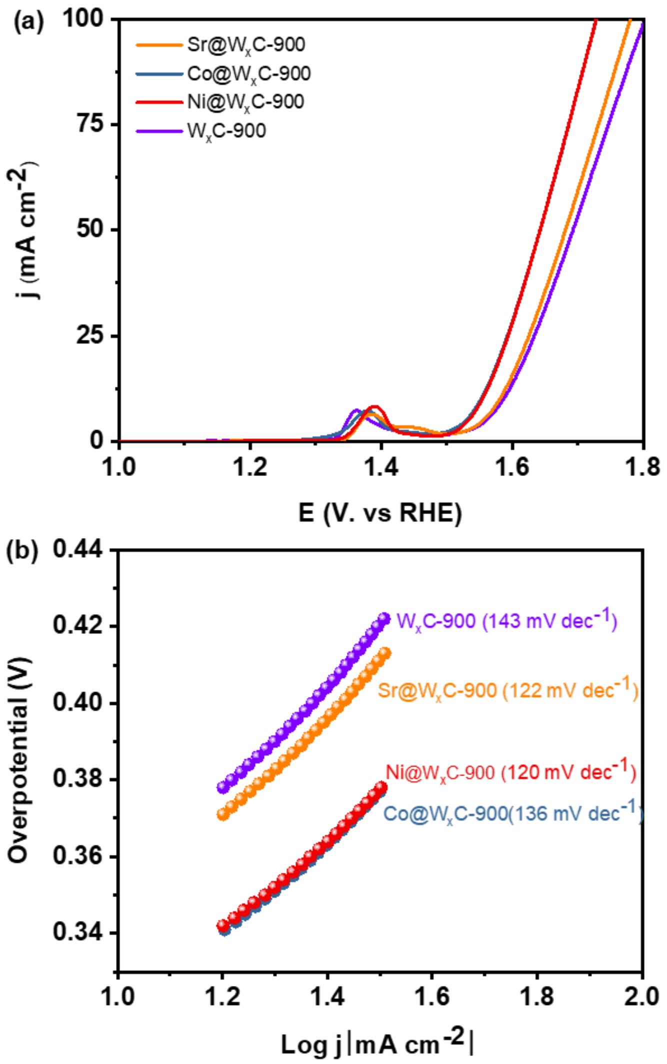

Figure S17. (a) OER polarization curve, and (b) tafel slope for Sr@WxC-900, Co@WxC-900, Ni@WxC-900, and WxC-900 is performed without iR correction using 1 M KOH with the scan rate of 5 mV.

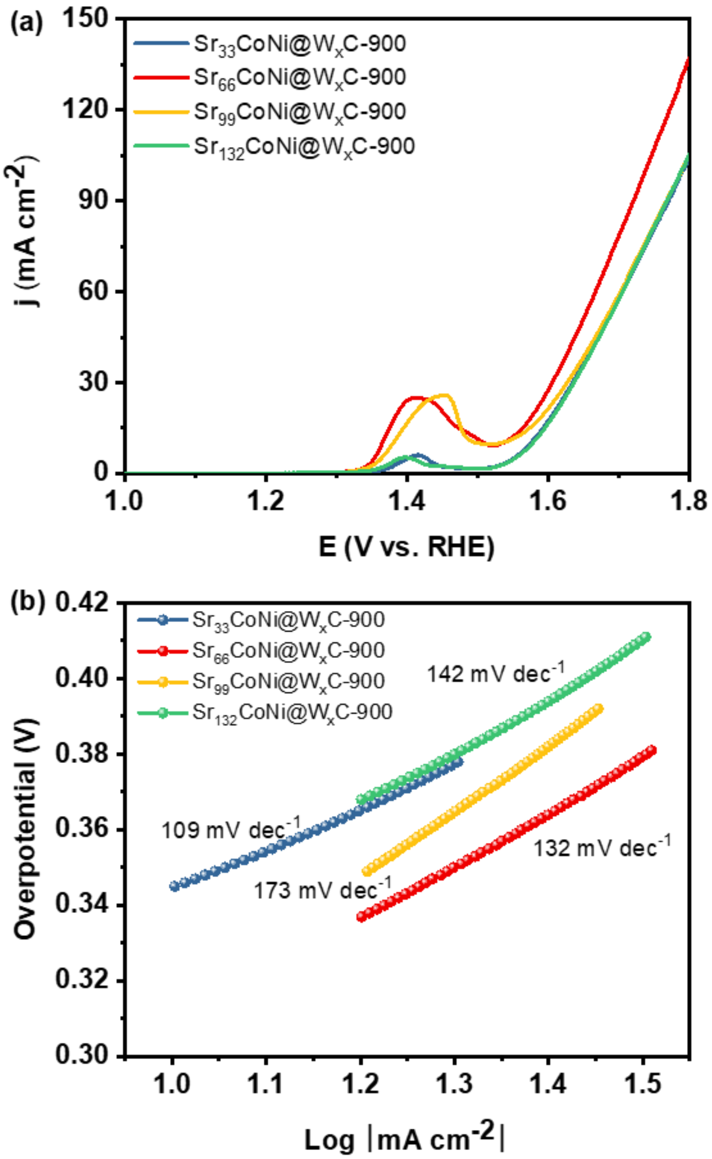

***Figure S18*.** (a) OER polarization curve, and (b) tafel slope for $Sr_{33}CoNi@W_xC$-900, $Sr_{66}CoNi@W_xC$-900, $Sr_{99}CoNi@W_xC$-900, and $Sr_{132}CoNi@W_xC$-900 is performed without the iR-correction using 1 M KOH with the scan rate of 5 mV.

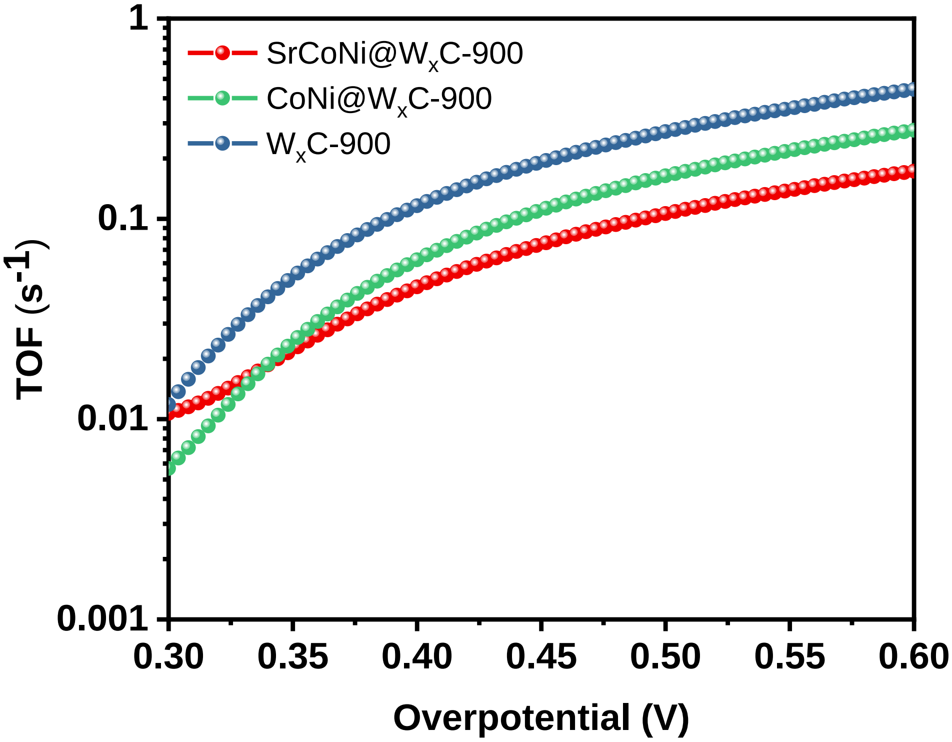

***Figure S19*.** Comparison of TOF for OER performance of SrCoNi@WxC-900, CoNi@WxC-900, and WxC-900.

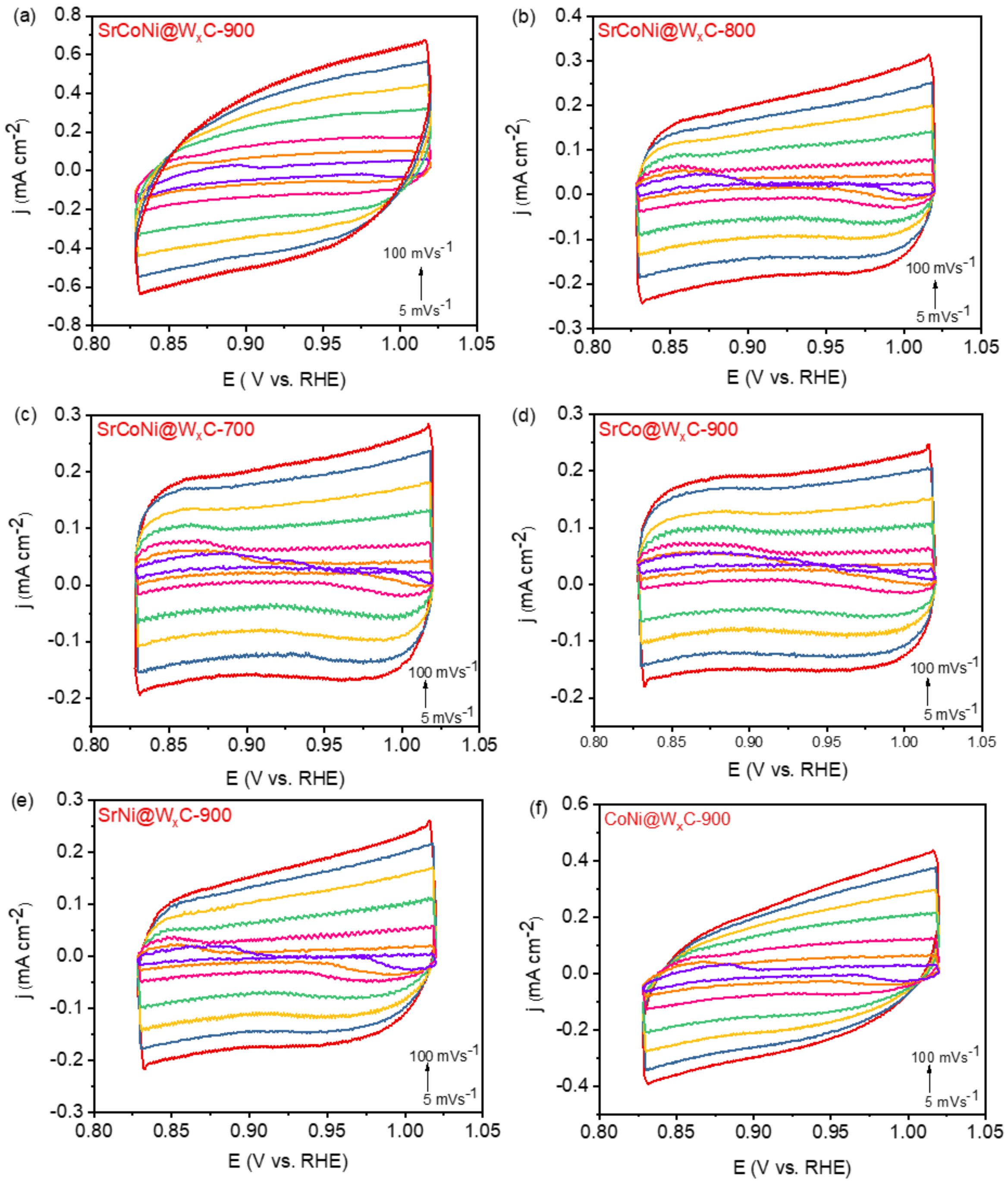

Figure S20. CV response of (a) SrCoNi@WxC-900, (b) SrCoNi@WxC-800, (c) SrCoNi@WxC-700, (d) SrCo@WxC-900, (e) SrNi@WxC-900, and (f) CoNi@WxC-900 is observed after the OER performance in the non-faradiac region from 0.824 to 1.024 V vs. RHE in 1 M KOH with the scan rate from 5 to 100 mVs^{-1}.

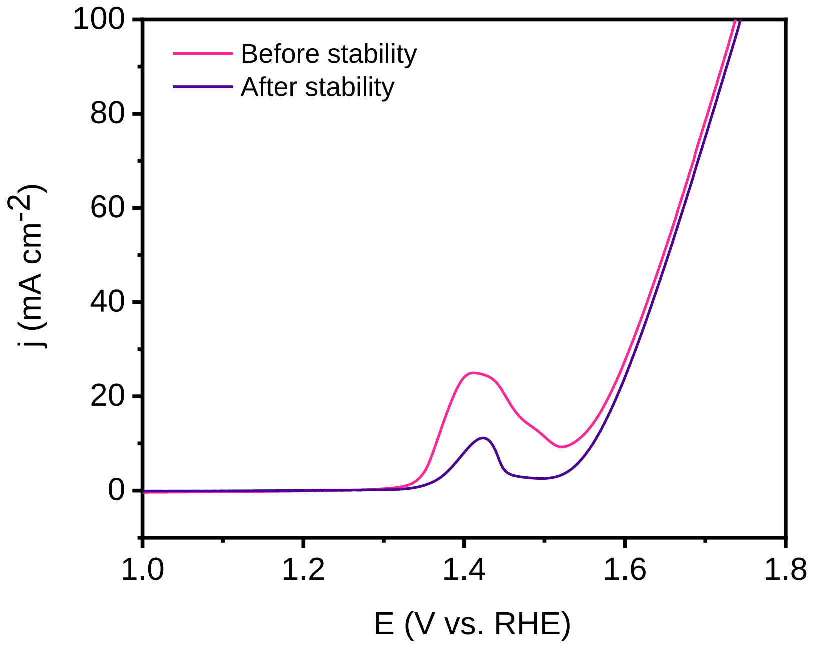

Figure S21. OER polarization curve of SrCoNi@WxC-900 is performed before and after the chronopotentiometry test for 60 hours without the iR-correction using 1 M KOH with the scan rate of 5 mV.

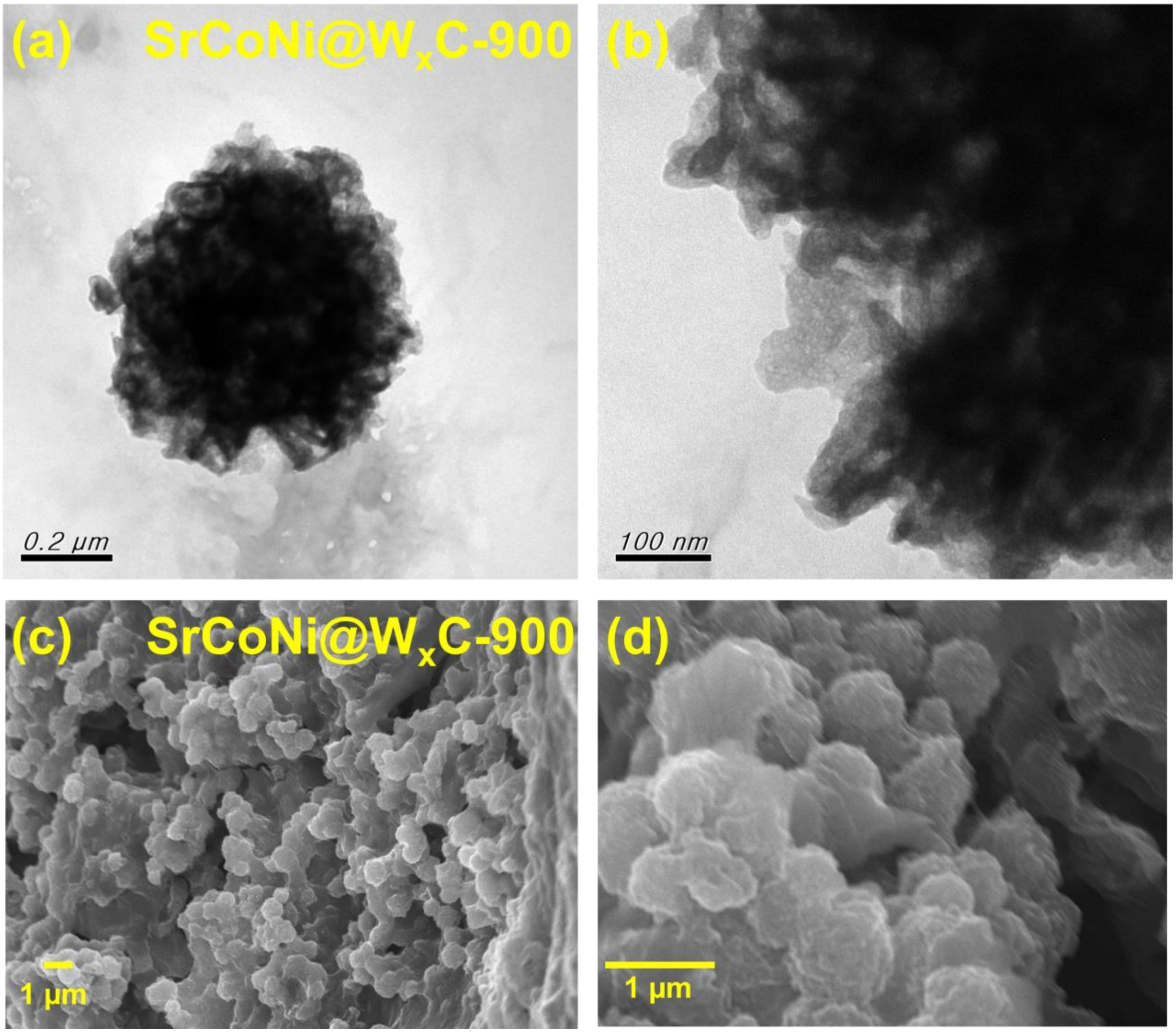

Figure S22. (a,b) TEM and (c,d) FE-SEM images of SrCoNi@W_xC-900 after the OER stability performance.

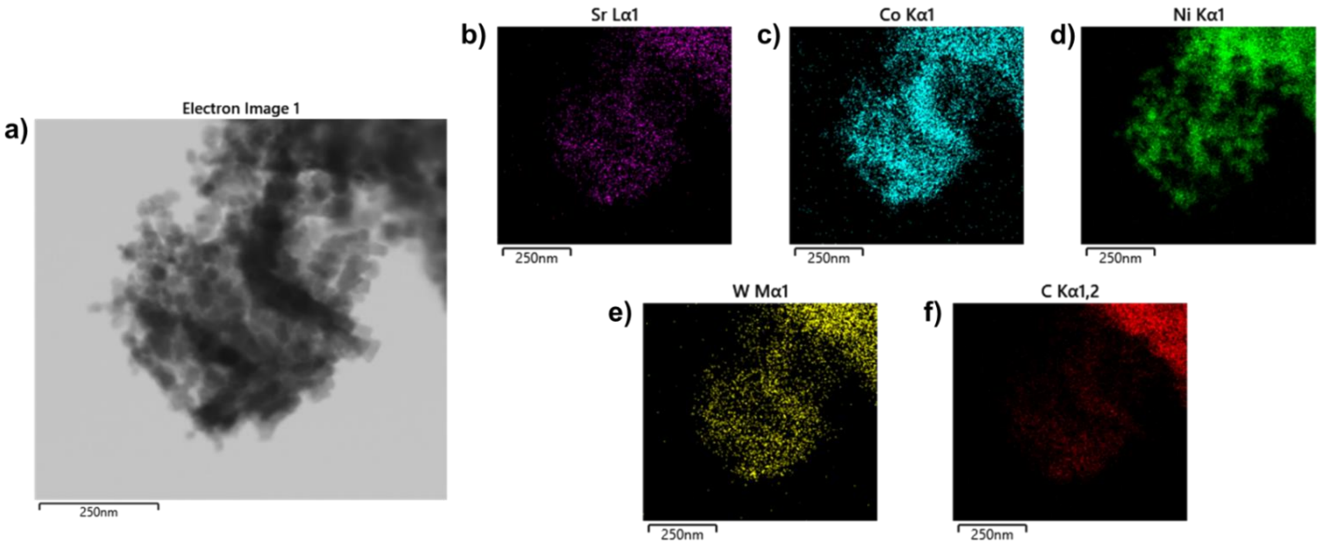

Figure S23. (a) HAADF-STEM image of SrCoNi@WxC-900 after the OER stability performance and (b-f) elemental mapping acquired over SrCoNi@WxC-900 for Sr, Co, Ni, W, and C - elements respectively.

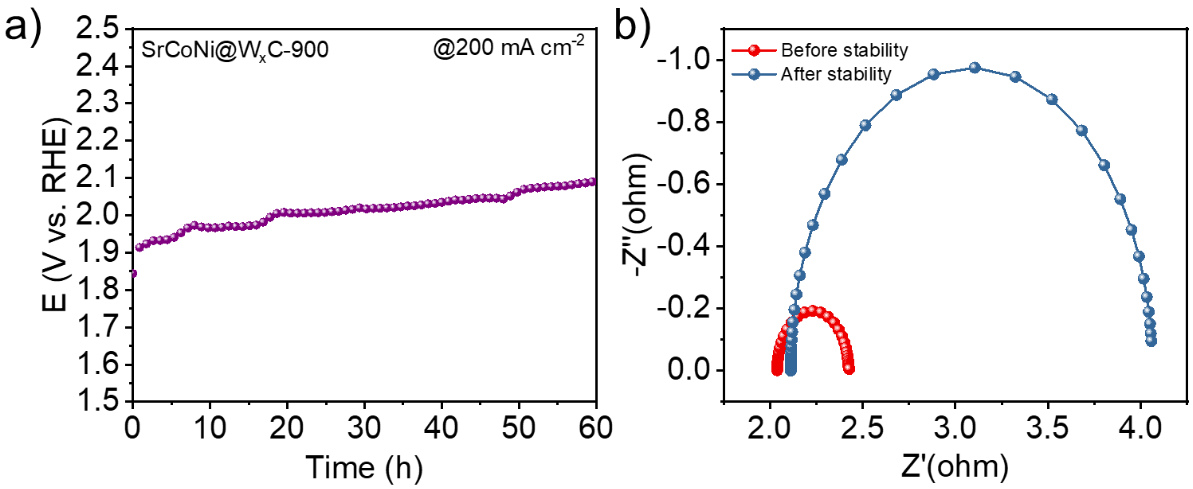

Figure S24. (a) OER stability performance of SrCoNi@WxC-900 at 200 mA cm-2 for 60 hour. (b) EIS spectra of SrCoNi@WxC-900 at catalytic turn over region before and after the stability test.

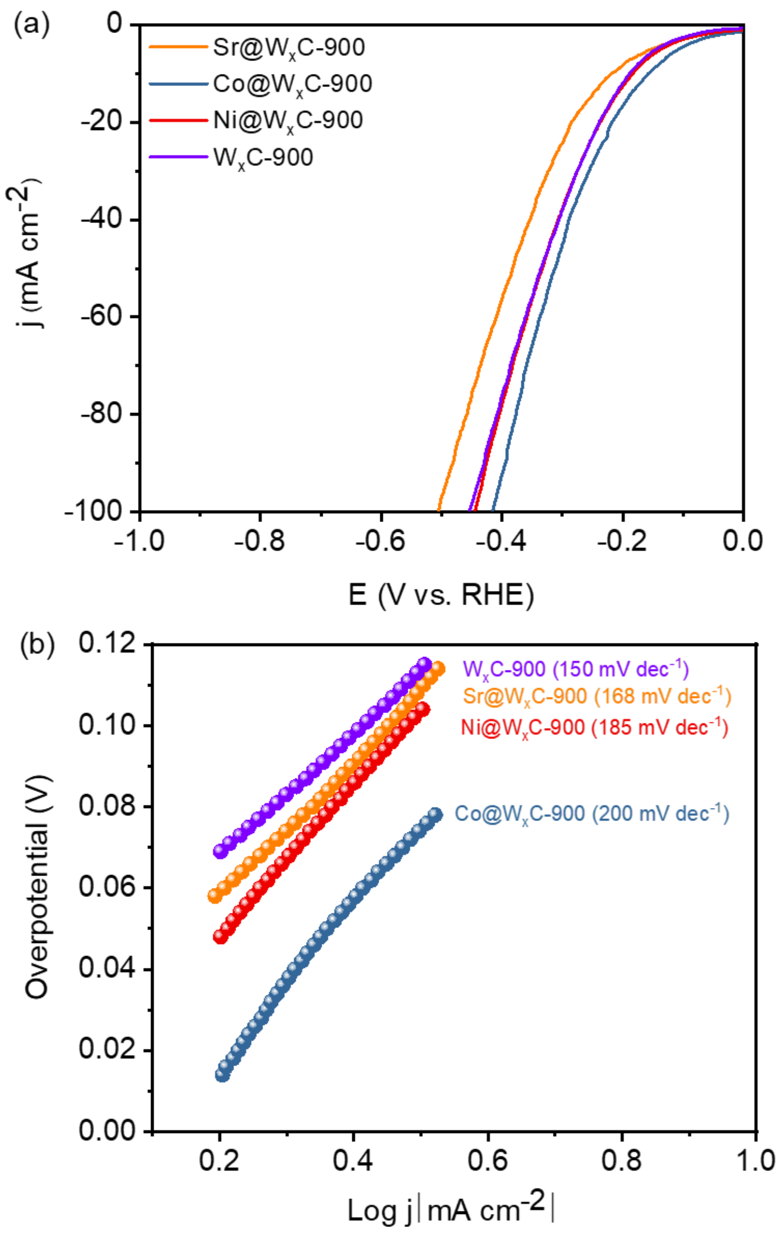

Figure S25. (a) HER polarization curve, (b) tafel slope for Sr@WxC-900, Co@WxC-900, Ni@WxC-900, and WxC-900 is performed without iR correction using 1 M KOH with the scan rate of 5 mV.

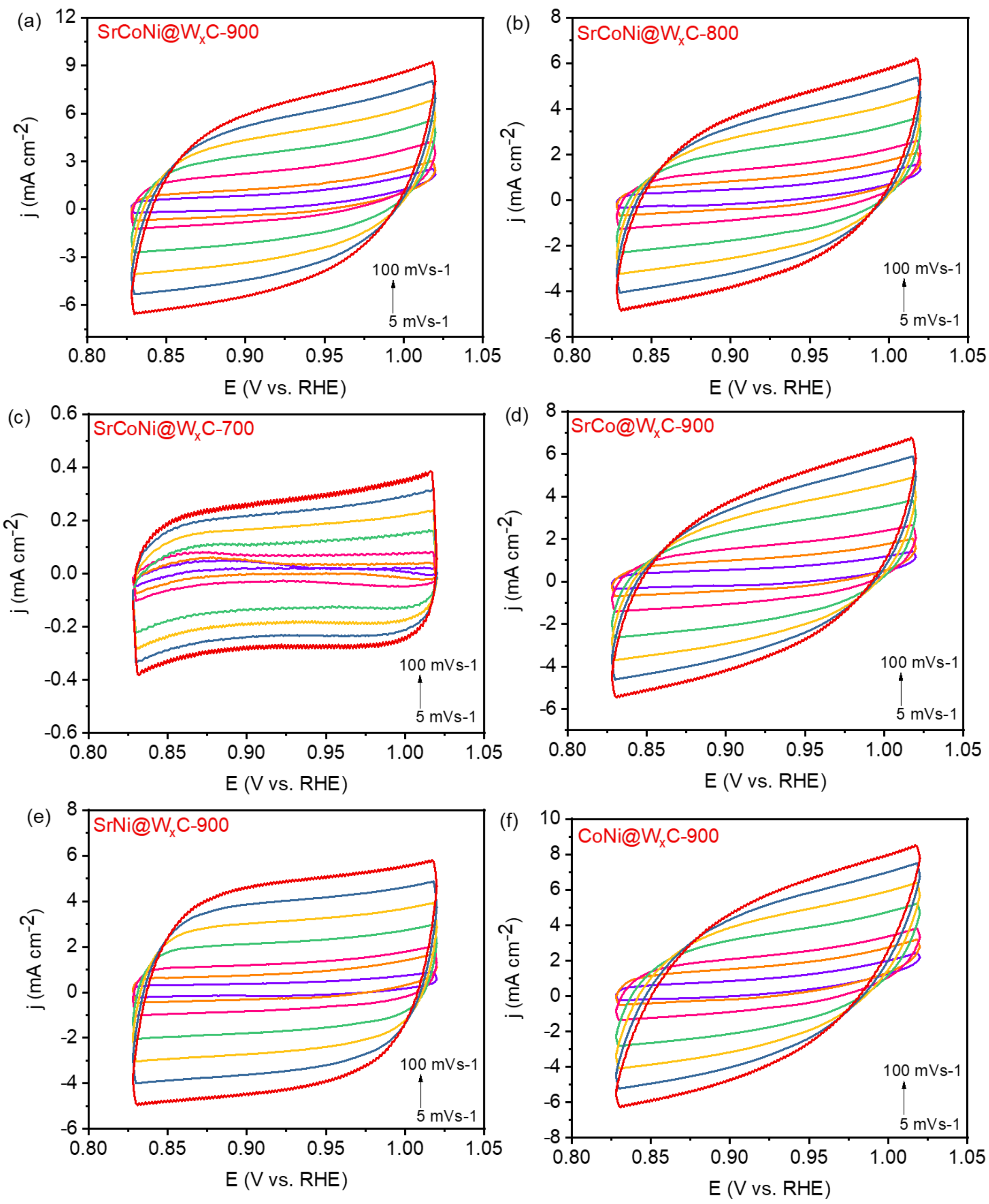

Figure S26**.** CV response of (a) SrCoNi@WxC-900, (b) SrCoNi@WxC-800, (c) SrCoNi@WxC-700, (d) SrCo@WxC-900, (e) SrNi@WxC-900, and (f) CoNi@WxC-900 is observed after the HER performance in the non-faradiac region from 0.824 to 1.024 V vs. RHE in 1 M KOH with the scan rate from 5 to 100 mVs^{-1}.

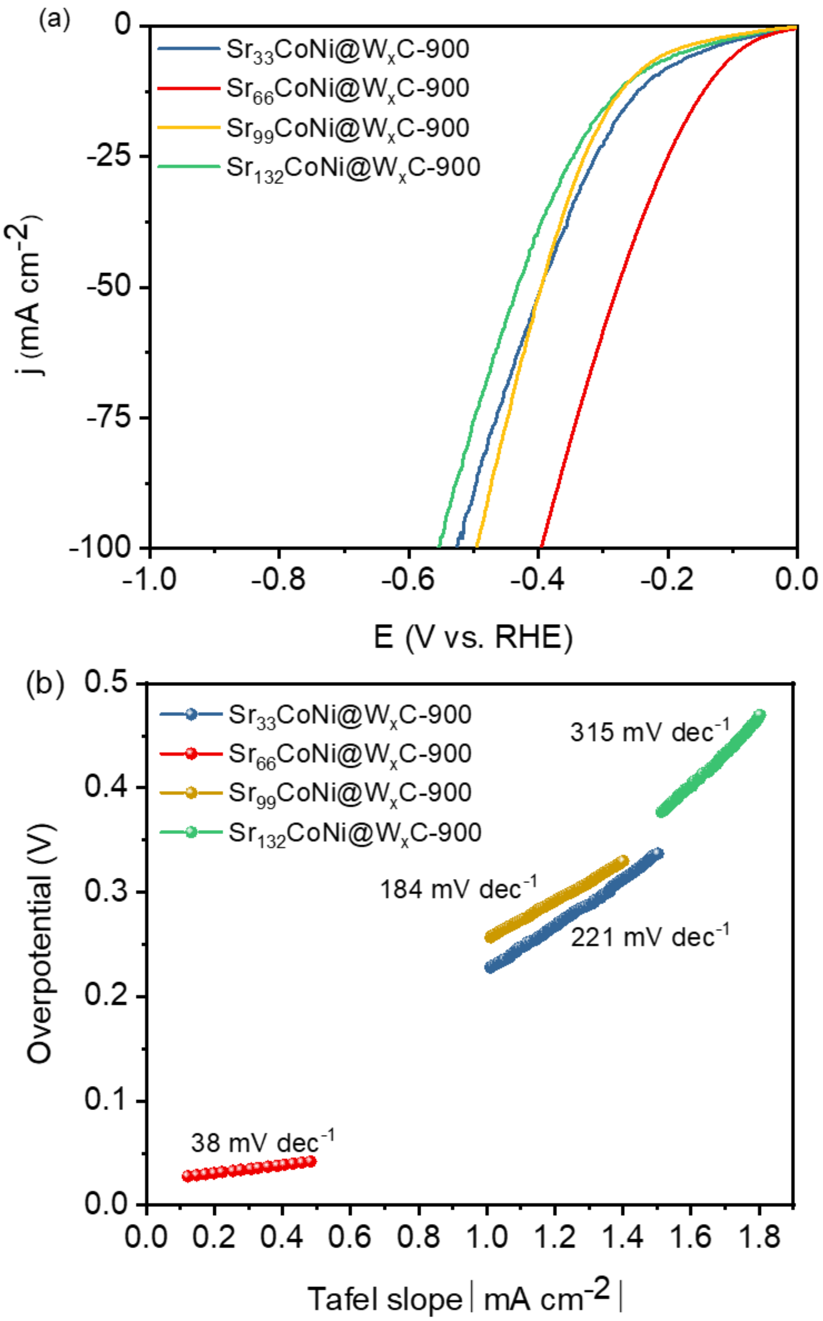

Figure S27. HER polarization curve of $Sr_{33}CoNi@W_xC$-900, $Sr_{66}CoNi@W_xC$-900, $Sr_{99}CoNi@W_xC$-900, and $Sr_{132}CoNi@W_xC$-900 is performed without the iR-correction using 1 M KOH with the scan rate of 5 mV.

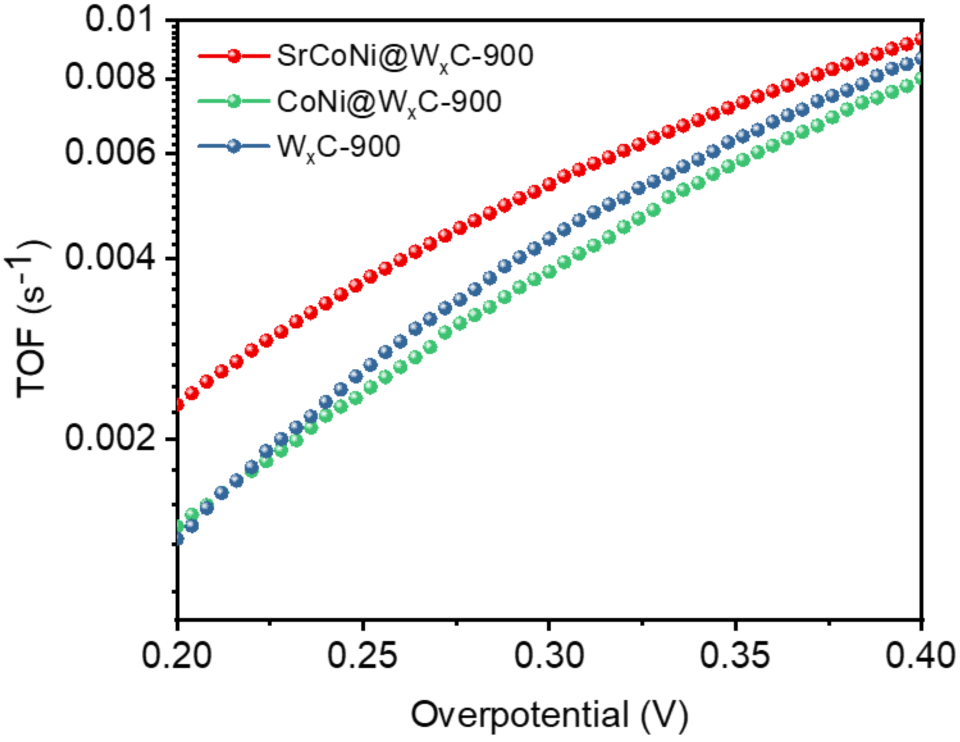

Figure S28. Comparison of TOF for HER performance of SrCoNi@W_xC-900, CoNi@W_xC-900, and W_xC-900.

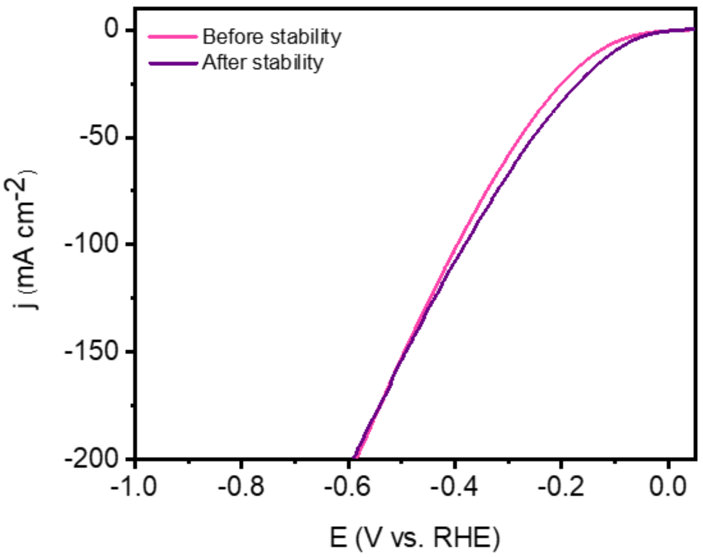

Figure S29**.** The HER polarization curve of SrCoNi@W_xC-900 is performed before and after the chronopotentiometry test for 60 hours without the iR-correction using 1 M KOH with the scan rate of 5 mV.

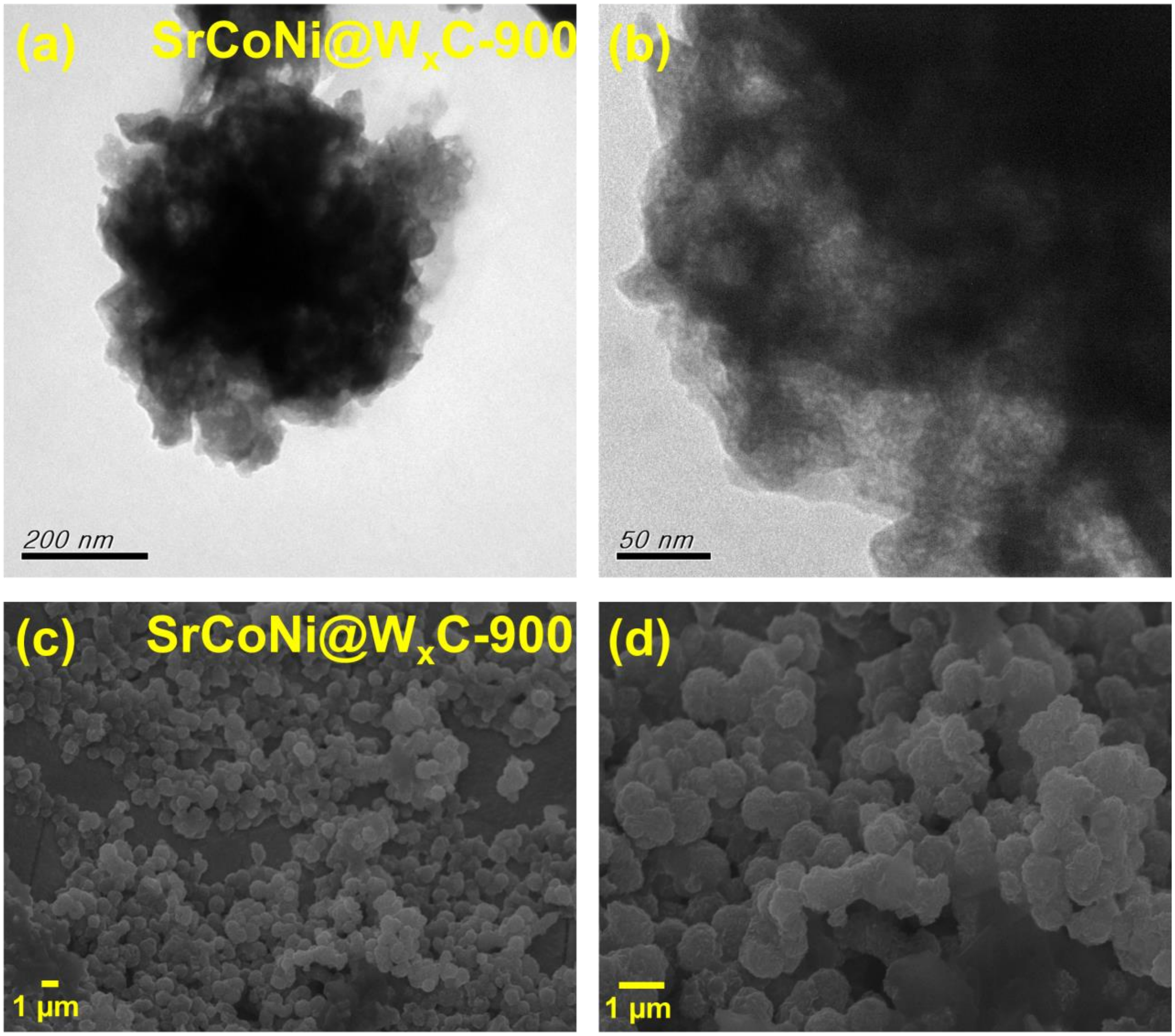

Figure S30. (a,b) TEM and (c,d) FE-SEM images of SrCoNi@W$_x$C-900 after the HER stability performance.

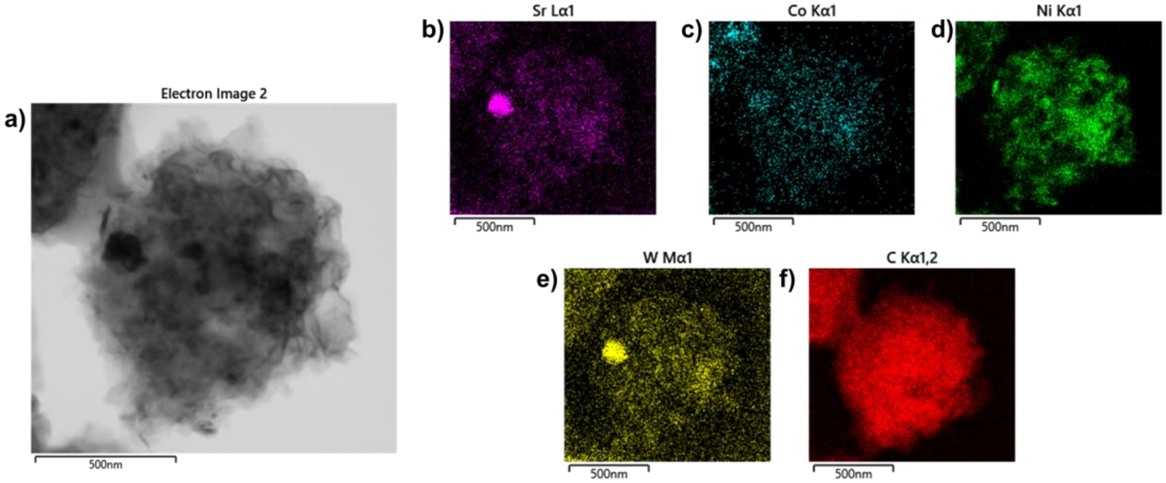

Figure S31. (a) HAADF-STEM image of SrCoNi@WxC-900 after the HER stability performance and (b-f) elemental mapping acquired over SrCoNi@WxC-900 for Sr, Co, Ni, W, and C - elements respectively.

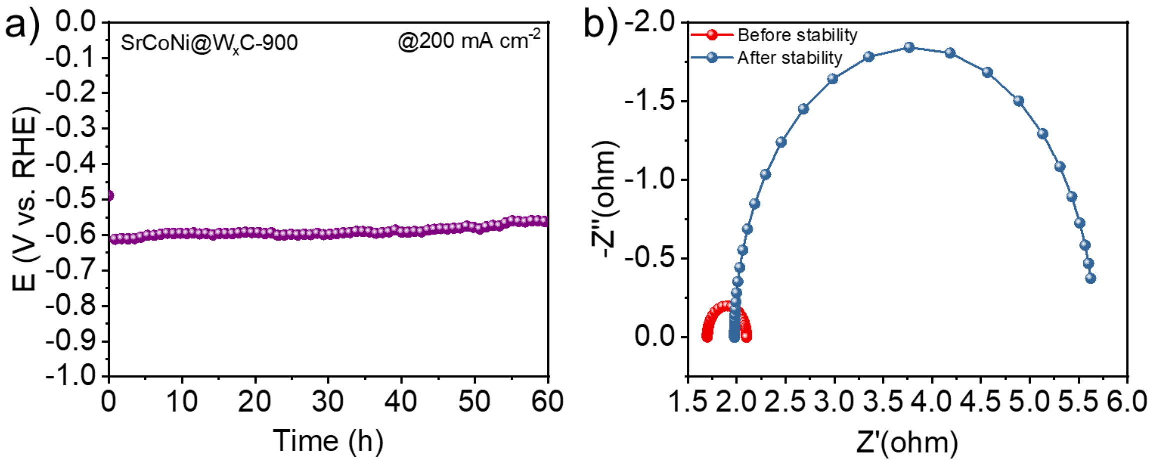

Figure S32. (a) HER stability performance of SrCoNi@WxC-900 at 200 mA cm-2 for 60 hour. (b) EIS spectra of SrCoNi@WxC-900 at catalytic turn over region before and after the stability test.

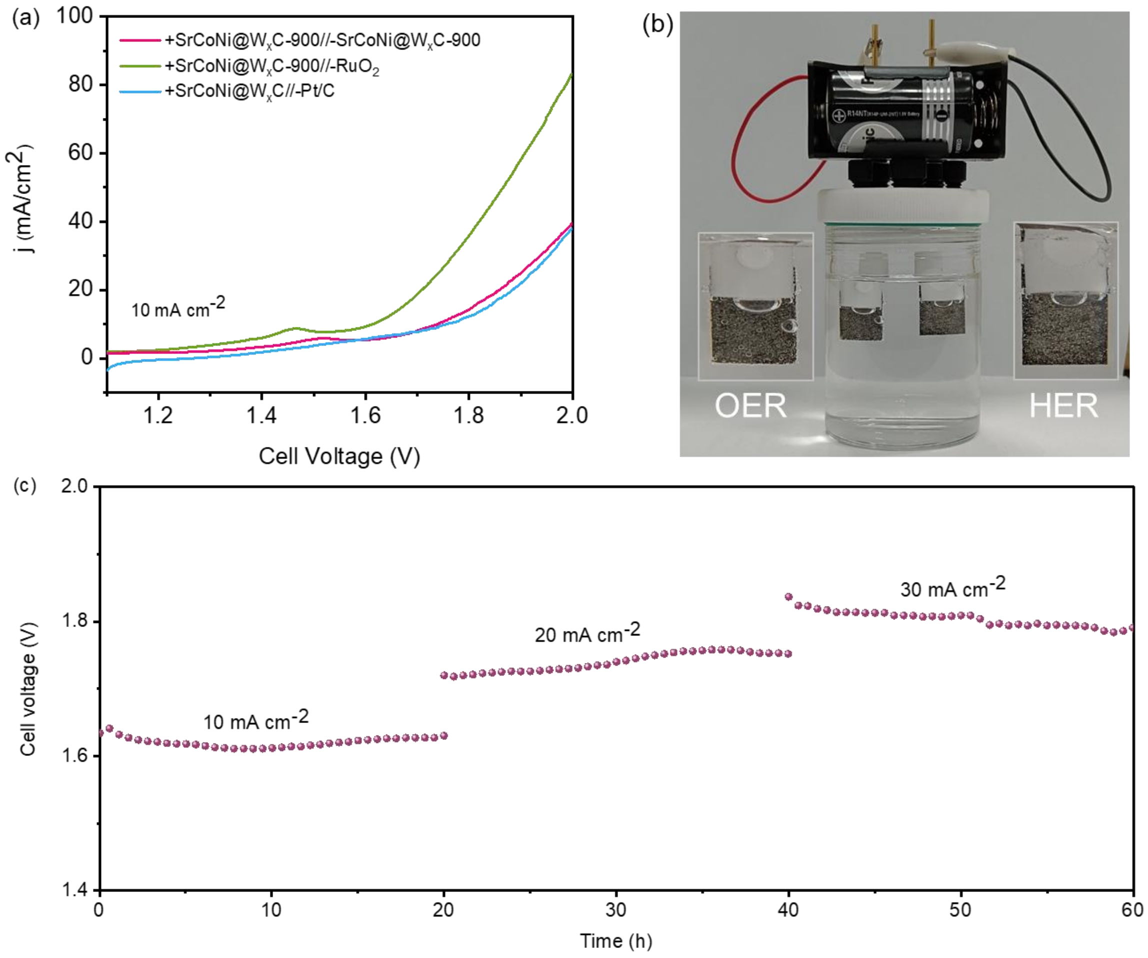

Figure S33. (a) Overall water splitting performance of SrCoNi@WxC-900 in H-cell with 1 M KOH electrolyte separated with the Nafion-117 membrane. (b) Digital photographic image of two electrode performance with 1.5 V battery. (c) Stability performance of SrCoNi@WxC-900 with various current density up to 60 hours.

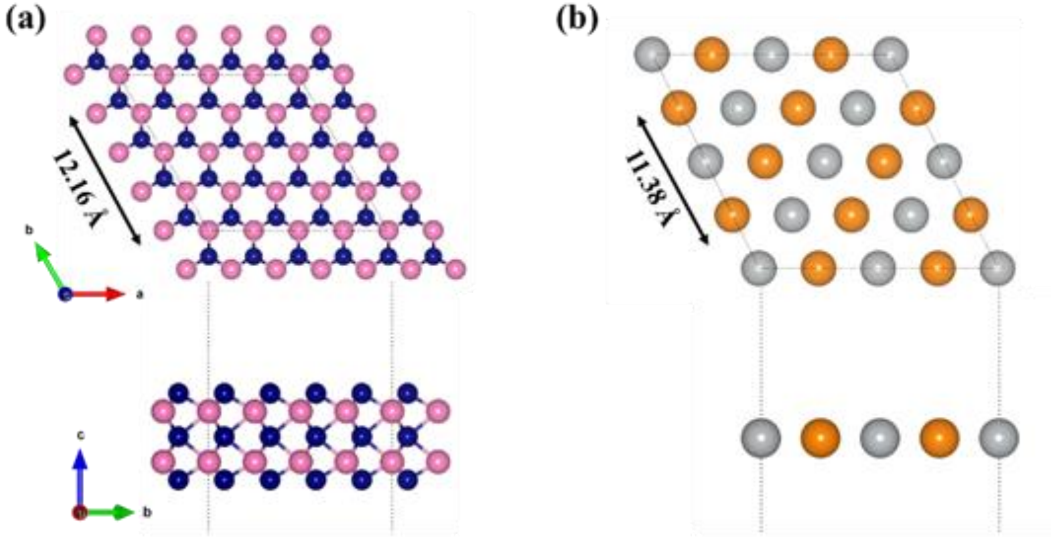

Figure S34. The optimized structures of the 4x4 supercells, showing top and side views of two compounds: (a) W_xC and (b) CoNi monolayer. Color code- W: pink, C: blue, Cu: orange, and Ni: silver.

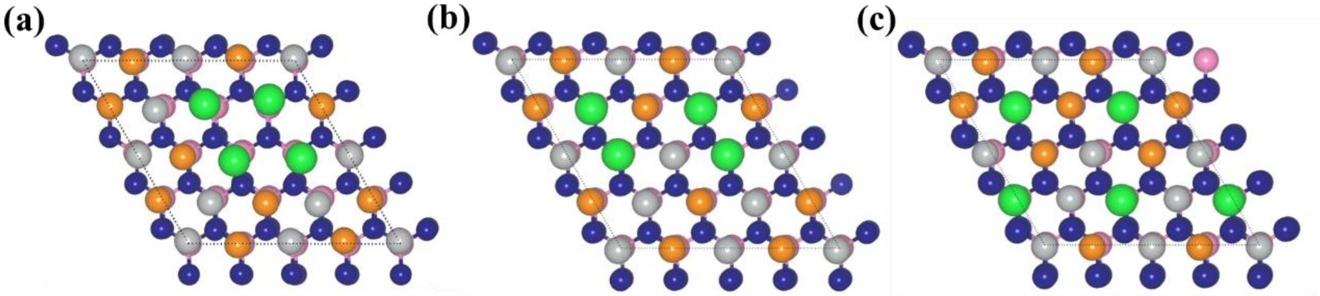

Figure S35. Different configurations of the Sr-doped heterostructure. (a) Sr-cluster (b) Sr-pair and (c) Sr-discrete. Color code - Sr: green.

Table S1. List of comparison of BJH surface area, pore volume and average pore diameter of the prepared samples.

**Sample**	**Surface area (m^2/g)**	**Pore volume (cm^3/g)**	**Average Pore diameter (nm)**
SrCoNi@WXC-900	128.49	0.228	7.018
SrCoNi@WXC-800	127.34	0.230	7.19
SrCoNi@WXC-700	146.26	0.213	5.82
SrCo@WXC-900	100.36	0.136	5.41
SrNi@WXC-900	191.05	0.235	4.92
CoNi@WXC-900	147.67	0.264	7.12
Sr@WXC-900	90.37	0.216	9.45
Co@WXC-900	132.08	0.249	7.48
Ni@WXC-900	214.08	0.275	5.09
WXC-900	220.70	0.218	3.93

Table S2. Atomic weight percentage of each elements calculated from the XPS data.

Sample	Sr atomic %	Co atomic %	Ni atomic %	W atomic %	C atomic %
SrCoNi@W_xC-900	0.21	0.65	0.62	7.87	90.65
SrCoNi@W_xC-800	0.25	0.50	0.55	7.87	90.84
SrCoNi@W_xC-700	0.21	0.64	0.68	9.87	88.60
SrCo@W_xC-900	0.42	0.81	-	3.48	95.30
SrNi@W_xC-900	0.41	-	0.82	5.81	92.95
CoNi@W_xC-900	-	0.68	0.53	4.37	94.42
Sr@W_xC-900	0.42	-	-	5.52	94.06
Co@W_xC-900	-	0.83	-	2.73	96.44
Ni@W_xC-900	-	-	0.84	4.11	95.05
W_xC-900	-	-	-	7.81	92.19

Table S3. Atomic weight percentage of each elements calculated from the ICP-MS data.

Sample	Sr atomic % (*ppm*)	Co atomic % (*ppm*)	Ni atomic % (*ppm*)	W atomic % (*ppm*)
SrCoNi@W_xC-900	<0.1	<0.1	<0.1	0.237
SrCoNi@W_xC-800	<0.1	<0.1	<0.1	0.205
SrCoNi@W_xC-700	<0.1	<0.1	<0.1	0.196
SrCo@W_xC-900	<0.1	<0.1	-	0.185
SrNi@W_xC-900	<0.1	-	<0.1	0.189
CoNi@W_xC-900	-	0.109	<0.1	0.186
Sr@W_xC-900	<0.1	-	-	0.190
Co@W_xC-900	-	<0.1	-	0.184
Ni@W_xC-900	-	-	<0.1	0.186
W_xC-900	-	-	-	0.190

Table S4. EXAFS parameters from the fitting.

Sample	Shell	Coordination number (CN)	Bond distance (R(Å))
SrCoNi@WxC	Ni-O/N/C	3.8	2.060
	Ni-W	2.4	2.980
	Ni-Sr	0.8	3.140
	Co-O/N/C	3.4	2.220
	Co-W	2.6	2.960
	Co-Sr	0.6	3.260

Table S5. Comparison of OER activity of SrCoNi@W_xC-900 with control samples.

**Catalyst**	**Electrolyte**	**Tafel slope *(mV dec^{-1})***	**Overpotential η@10 mA cm^{-2} (OER)**
SrCoNi@W_xC-900	1 M KOH	132	300
SrCoNi@W_xC-800	1 M KOH	138	337
SrCoNi@W_xC-700	1 M KOH	142	329
SrCo@W_xC-900	1 M KOH	134	341
SrNi@W_xC-900	1 M KOH	150	359
CoNi@W_xC-900	1 M KOH	128	339
Sr@W_xC-900	1 M KOH	143	357
Co@W_xC-900	1 M KOH	136	322
Ni@W_xC-900	1 M KOH	122	351
W_xC-900	1 M KOH	120	324

Table S6. Electrochemical active surface area (ECSA) and double layer capacitance analysis of electrocatalyst performed after the OER performance at the non-faradiac region.

Catalyst	Double layer capacitance (C_{dl})(m*F* cm^{-2})	Electrochemical active surface area (ECSA) (cm^2)
SrCoNi@W_xC-900	4.6	115
SrCoNi@W_xC-800	2.1	52.5
SrCoNi@W_xC-700	2.0	50
SrCo@W_xC-900	2.2	55
SrNi@W_xC-900	2.1	52.5
CoNi@W_xC-900	2.7	67.5

Table S7. Electrochemical impedance Spectroscopy analysis of electrocatalyst performed after the OER performance with the η@10 mA cm^{-2}.

**Catalyst**	**Solution resistance (*Rs*) (*Ω*)**	**Charge transfer resistance (*Rct*)(*mΩ*)**
SrCoNi@W_xC-900	1.77	0.42
SrCoNi@W_xC-800	1.96	0.99
SrCoNi@W_xC-700	1.83	0.50
SrCo@W_xC-900	1.92	1.18
SrNi@W_xC-900	1.68	7.05
CoNi@W_xC-900	1.76	0.65

Table S8. Comparison of OER activity of SrCoNi@W_XC-900 with the reported literature.

Catalyst	Electrolyte	Tafel slope *(mV dec^{-1})*	Overpotential η@10 mA cm^{-2} (*mV*)	Reference
**SrCoNi@W_XC-900**	**1 M KOH**	**132**	**300**	**This work**
TMB@NiNC (NiCoFeB@NiNC)	1 M KOH	53.9	208	**(12)**
Fe1.0Co1.1Ni1.4-NC	1 M KOH	60	270	**(13)**
FeCoNi@NG	1 M KOH	60	310	**(14)**
FeCo/FeCoNi@NCNTs-HF		57	378	**(15)**
(Fe,Ni)@N-MWXCNTs	1 M KOH	56.2	319	**(16)**
NiCoFe-LDHs	1 M KOH	92	288	**(17)**
M-NC-CoCu	1M KOH	77	310	**(18)**
NixCo1–x@NixCo1–xO/NCNT	1M KOH	65	380	**(19)**
FeCo/Co_2P@NPCF	1M KOH	61	330	**(20)**
Co/CoO@NC@CC	1M KOH	76	269	**(21)**
Ru-Ni-Co-P/NC	1M KOH	84	318	**(22)**
CoP/NiCoP	1M KOH	104.5	310	**(23)**
Ni_3S_2/MoS_2/WS_2	1M KOH	97	290	**(24)**
Co_3O_4@Mo–Co_3S_4–Ni_3S_2/NF	1M KOH	98	295	**(25)**

Table S9. Comparison of HER activity of SrCoNi@W_xC-900 with control catalyst.

Catalyst	Electrolyte	Tafel slope *(mV dec^{-1})*	Overpotential η@10 mA cm^{-2} (HER)
SrCoNi@W_xC-900	1 M KOH	38	130
SrCoNi@W_xC-800	1 M KOH	103	174
SrCoNi@W_xC-700	1 M KOH	80	175
SrCo@W_xC-900	1 M KOH	102	238
SrNi@W_xC-900	1 M KOH	100	199
CoNi@W_xC-900	1 M KOH	73	176
Sr@W_xC-900	1 M KOH	168	218
Co@W_xC-900	1 M KOH	200	157
Ni@W_xC-900	1 M KOH	185	182
W_xC-900	1 M KOH	150	189

Table S10. Electrochemical active surface area (ECSA) analysis of electrocatalyst performed after the HER performance at the non-faradiac region.

Catalyst	Double layer capacitance (Cdl)(m*F cm*$^{-2}$)	Electrochemical active surface area (ECSA) (cm^{-2})
SrCoNi@W_xC-900	112	2800
SrCoNi@W_xC-800	99	2475
SrCoNi@W_xC-700	5	125
SrCo@W_xC-900	72	1800
SrNi@W_xC-900	76	1900
CoNi@W_xC-900	93	2325

Table S11. Electrochemical impedance Spectroscopy analysis of electrocatalyst performed after the HER performance with the η@10 mA cm^{-2}.

**Catalyst**	**Solution resistance (*Rs*) (*Ω*)**	**Charge transfer resistance (*Rct*)(*mΩ*)**
SrCoNi@W_xC-900	1.78	0.30
SrCoNi@W_xC-800	1.83	0.50
SrCoNi@W_xC-700	1.65	2.17
SrCo@W_xC-900	1.91	0.66
SrNi@W_xC-900	1.97	0.56
CoNi@W_xC-900	1.69	0.40

Table S12. Comparison of HER activity of SrCoNi@W_xC-900 with the reported literature.

**Catalyst**	**Electrolyte**	**Tafel slope *(mV dec^{-1})***	**Overpotential η@10 mA cm^{-2} (mV)**	**Reference**
**SrCoNi@W_XC-900**	**1 M KOH**	**38**	**130**	**This work**
TMB@NiNC (NiCoFeB@NiNC)	1 M KOH	127.9	174	**(12)**
Fe1.0Co1.1Ni1.4-NC	1 M KOH	168	175	**(13)**
FeCoNi@NG	1 M KOH	-	255	**(14)**
M-NC-CoCu	1M KOH	164	240	**(18)**
FeCo/Co_2P@NPCF	1M KOH	120	260	**(20)**
Co/CoO@NC@CC	1M KOH	80	152	**(21)**
CoP/NiCoP	1M KOH	83.7	110	**(22)**
Ni_3S_2/MoS_2/WS_2	1M KOH	77	165	**(23)**
Co_3O_4@Mo–Co_3S_4–Ni_3S_2/NF	1M KOH	97	116	**(24)**
FeCoMoS@NG	1 M KOH	85	137	**(26)**
CoNi-MoS_2/NCNs	1M KOH	-	124.9	**(27)**
NiFe-LDH@CoS_x	1M KOH	-	136	**(28)**
NiFeNb-0.25	1M KOH	-	207	**(29)**
Fe_3C–Fe/NC-800	1M KOH	56.6	124	**(30)**
Cu3N@CoNiCHs@CF	1M KOH	134	182	**(31)**

Table S13. Comparison of overall water splitting activity of SrCoNi@W_xC-900 with the reported literature.

Catalyst	Electrolyte	Cell voltage (V) @10 mA cm^{-2}	Reference
SrCoNi@W_xC-900**//** SrCoNi@W_xC-900	1 M KOH	1.73	**This work**
SrCoNi@W_xC-900**//** RuO_2	1 M KOH	1.61	**This work**
SrCoNi@W_xC-900**//** Pt/C	1 M KOH	1.76	**This work**
Co/CoO@NC@CC	1M KOH	1.66	**(21)**
CoP/NiCoP	1M KOH	1.63	**(23)**
$Ni_3S_2/MoS_2/WS_2$	1M KOH	1.73	**(24)**
Co_3O_4@Mo–Co_3S_4–Ni_3S_2/NF	1M KOH	1.62	**(25)**
$MoS_2/NiPS_3$	1 M KOH	1.64	**(32)**
Fe–Ni_2P@PC/Cu_xS	1 M KOH	1.62	**(33)**
NiCoP	1 M KOH	1.58	**(34)**
RuCu NSs/C	0.1 M KOH	1.55	**(35)**
CoRu@NCHNSs	1 M KOH	1.56	**(36)**
$Ni_{0.7}Fe_{0.3}PS_3$@MXene‖$Ni_{0.9}Fe_{0.1}PS_3$@MXene	1 M KOH	1.65	**(37)**
CoSAs-MoS2/TiN NRs	1 M KOH	1.65	**(38)**
Co-Ni-P films couple	1 M KOH	1.64	**(39)**
P@pCoPc-1/Co3O4	1 M KOH	1.67	**(40)**

Table S14. The calculated formation energy of different Sr-doped heterostructures.

**System**	**E_f (eV)**
Cluster	-13.78
Pair	-14.85
Discrete	-15.72

Table S15. Calculated properties of the Sr-doped and undoped heterostructure, including the total magnetic moment (M), bond lengths, interlayer distance (d) and binding energy (Eb).

**System**	**E_b (eV)**	**M (μ_B)**	**Bond Length (Å)**		**d (Å)**
			W-C	Ni-Co	
W_xC	-	0.0	2.03-2.28	-	-
NiCo	-	26.04	-	2.51	-
NiCo@W_xC	0.23	28.80	2.19-2.21	2.95-3.0	1.15
SrNiCo@W_xC	0.22	20.83	2.14-2.29	2.86-2.97	1.14

S5. Reference

(1) Blöchl, P. E. Projector Augmented-Wave Method. *Phys. Rev. B* **1994**, *50* (24), 17953.

(2) Kresse, G.; Furthmüller, J. Efficient Iterative Schemes for Ab Initio Total-Energy Calculations Using a Plane-Wave Basis Set. *Phys. Rev. B* **1996**, *54* (16), 11169.

(3) Korzhavyi, P. A.; Abrikosov, I. A.; Johansson, B.; Ruban, A. V; Skriver, H. L. First-Principles Calculations of the Vacancy Formation Energy in Transition and Noble Metals. *Phys. Rev. B* **1999**, *59* (18), 11693.

(4) Perdew, J. P.; Burke, K.; Ernzerhof, M. Generalized Gradient Approximation Made Simple. *Phys. Rev. Lett.* **1996**, *77* (18), 3865.

(5) Karmakar, A.; Mahendiran, D.; Madhu, R.; Murugan, P.; Kundu, S. Bypassing the Scaling Relationship with Spin Selectivity: Construction of Lewis Base-Functionalized Heterostructural 2D Nanosheets for Enhanced Oxygen Evolution Reaction. *J. Mater. Chem. A* **2023**, *11* (30), 16349–16362.

(6) Grimme, S.; Antony, J.; Ehrlich, S.; Krieg, H. A Consistent and Accurate Ab Initio Parametrization of Density Functional Dispersion Correction (DFT-D) for the 94 Elements H-Pu. *J. Chem. Phys.* **2010**, *132* (15).

(7) Grimme, S.; Ehrlich, S.; Goerigk, L. Effect of the Damping Function in Dispersion Corrected Density Functional Theory. *J. Comput. Chem.* **2011**, *32* (7), 1456–1465.

(8) Henkelman, G.; Arnaldsson, A.; Jónsson, H. A Fast and Robust Algorithm for Bader Decomposition of Charge Density. *Comput. Mater. Sci.* **2006**, *36* (3), 354–360.

(9) Li, M.; Zhang, L.; Xu, Q.; Niu, J.; Xia, Z. N-Doped Graphene as Catalysts for Oxygen Reduction and Oxygen Evolution Reactions: Theoretical Considerations. *J. Catal.* **2014**, *314*, 66–72.

(10) Man, I. C.; Su, H.; Calle- Vallejo, F.; Hansen, H. A.; Martínez, J. I.; Inoglu, N. G.; Kitchin, J.; Jaramillo, T. F.; Nørskov, J. K.; Rossmeisl, J. Universality in Oxygen Evolution Electrocatalysis on Oxide Surfaces. *ChemCatChem* **2011**, *3* (7), 1159–1165.

(11) Momma, K.; Izumi, F. VESTA 3 for Three-Dimensional Visualization of Crystal, Volumetric and Morphology Data. *J. Appl. Crystallogr.* **2011**, *44* (6), 1272–1276.

(12) Moloudi, M.; Noori, A.; Rahmanifar, M. S.; Shabangoli, Y.; El- Kady, M. F.; Mohamed, N. B.; Kaner, R. B.; Mousavi, M. F. Layered Double Hydroxide Templated Synthesis of Amorphous NiCoFeB as a Multifunctional Electrocatalyst for Overall Water Splitting and Rechargeable Zinc–Air Batteries (Adv. Energy Mater. 4/2023). *Adv. Energy Mater.* **2023**, *13* (4), 2370014.

(13) Khalid, M.; Honorato, A. M. B.; Tremiliosi Filho, G.; Varela, H. Trifunctional Catalytic Activities of Trimetallic FeCoNi Alloy Nanoparticles Embedded in a Carbon Shell for Efficient Overall Water Splitting. *J. Mater. Chem. A* **2020**, *8* (18), 9021–9031.

(14) Yang, Y.; Lin, Z.; Gao, S.; Su, J.; Lun, Z.; Xia, G.; Chen, J.; Zhang, R.; Chen, Q. Tuning Electronic Structures of Nonprecious Ternary Alloys Encapsulated in Graphene Layers for Optimizing Overall Water Splitting Activity. *Acs Catal.* **2017**, *7* (1), 469–479.

(15) Wang, Z.; Ang, J.; Zhang, B.; Zhang, Y.; Ma, X. Y. D.; Yan, T.; Liu, J.; Che, B.; Huang, Y.; Lu, X. FeCo/FeCoNi/N-Doped Carbon Nanotubes Grafted Polyhedron-Derived Hybrid Fibers as Bifunctional Oxygen Electrocatalysts for Durable Rechargeable Zinc–Air Battery.

Appl. Catal. B Environ. **2019**, *254*, 26–36.

(16) Jing, Q.; Mei, Z.; Sheng, X.; Zou, X.; Yang, Y.; Zhang, C.; Wang, L.; Sun, Y.; Duan, L.; Guo, H. 3d Orbital Electron Engineering in Oxygen Electrocatalyst for Zinc-Air Batteries. *Chem. Eng. J.* **2023**, *462*, 142321.

(17) Zhang, M.; Liu, Y.; Liu, B.; Chen, Z.; Xu, H.; Yan, K. Trimetallic NiCoFe-Layered Double Hydroxides Nanosheets Efficient for Oxygen Evolution and Highly Selective Oxidation of Biomass-Derived 5-Hydroxymethylfurfural. *Acs Catal.* **2020**, *10* (9), 5179–5189.

(18) Andrade, A. M.; Liu, Z.; Grewal, S.; Nelson, A. J.; Nasef, Z.; Diaz, G.; Lee, M. H. MOF-Derived Co/Cu-Embedded N-Doped Carbon for Trifunctional ORR/OER/HER Catalysis in Alkaline Media. *Dalt. Trans.* **2021**, *50* (16), 5473–5482.

(19) Jena, R.; Bhattacharyya, S.; Bothra, N.; Kashyap, V.; Pati, S. K.; Maji, T. K. Ni x Co1–X@ Ni x Co1–x O/NCNT as Trifunctional ORR, OER, and HER Electrocatalysts and Its Application in a Zn–Air Battery. *ACS Appl. Mater. Interfaces* **2023**, *15* (23), 27893–27904.

(20) Shi, Q.; Liu, Q.; Ma, Y.; Fang, Z.; Liang, Z.; Shao, G.; Tang, B.; Yang, W.; Qin, L.; Fang, X. High- performance Trifunctional Electrocatalysts Based on FeCo/Co2P Hybrid Nanoparticles for Zinc–Air Battery and Self- powered Overall Water Splitting. *Adv. Energy Mater.* **2020**, *10* (10), 1903854.

(21) Dai, K.; Zhang, N.; Zhang, L.; Yin, L.; Zhao, Y.; Zhang, B. Self-Supported Co/CoO Anchored on N-Doped Carbon Composite as Bifunctional Electrocatalyst for Efficient Overall Water Splitting. *Chem. Eng. J.* **2021**, *414*, 128804.

(22) Wang, D.; Gu, L.; Luo, X.; Su, R.; Shang, Y.; Wang, Y.; Hao, S.; Yang, Y. Multisite Engineering towards Atomically Dispersed Ru on Ni-Co-P Composite with N-Doped Carbon Matrix for Robust Water Oxidation. *J. Electroanal. Chem.* **2022**, *924*, 116875.

(23) Fu, X.; Zhang, Z.; Zheng, Y.; Lu, J.; Cheng, S.; Su, J.; Wei, H.; Gao, Y. Cobalt Phosphide/Nickel–Cobalt Phosphide Heterostructured Hollow Nanoflowers for High-Performance Supercapacitor and Overall Water Splitting. *J. Colloid Interface Sci.* **2024**, *653*, 1272–1282.

(24) Duraisamy, P.; Mummoorthi, G.; Jayaram, A.; Easwaran, S. K.; Mani, N. A Novel Method for Engineering Active Sites through In-Situ and Chemical Deposition: Robust Ni3S2/MoS2/WS2 Heterostructures for Efficient Water Splitting in an Alkaline Medium. *Int. J. Hydrogen Energy* **2024**, *92*, 186–198.

(25) Wu, Q.; Dong, A.; Yang, C.; Ye, L.; Zhao, L.; Jiang, Q. Metal-Organic Framework Derived Co3O4@ Mo-Co3S4-Ni3S2 Heterostructure Supported on Ni Foam for Overall Water Splitting. *Chem. Eng. J.* **2021**, *413*, 127482.

(26) Ramakrishnan, S.; Balamurugan, J.; Vinothkannan, M.; Kim, A. R.; Sengodan, S.; Yoo, D. J. Nitrogen-Doped Graphene Encapsulated FeCoMoS Nanoparticles as Advanced Trifunctional Catalyst for Water Splitting Devices and Zinc–Air Batteries. *Appl. Catal. B Environ.* **2020**, *279*, 119381.

(27) Khan, I.; Chen, Y.; Li, Z.; Liu, W.; Khan, S.; Ullah, S.; Liu, L.; Zada, A.; Ali, S.; Shaheen, S. Non-Covalent Interaction of Atomically Dispersed Dual-Site Catalysts Featuring Co and Ni Nascent Pair Sites for Efficient Electrocatalytic Overall Water Splitting. *J. Mater. Sci. Technol.* **2024**, *178*, 210–225.

(28) Yang, Y.; Xie, Y.; Yu, Z.; Guo, S.; Yuan, M.; Yao, H.; Liang, Z.; Lu, Y. R.; Chan, T.-S.; Li,

C. Self-Supported NiFe-LDH@ CoSx Nanosheet Arrays Grown on Nickel Foam as Efficient Bifunctional Electrocatalysts for Overall Water Splitting. *Chem. Eng. J.* **2021**, *419*, 129512.

(29) Jiang, K.; Li, Q.; Lei, S.; Zhai, M.; Cheng, M.; Xu, L.; Deng, Y.; Xu, H.; Bao, J. Nb-Doped NiFe LDH Nanosheet with Superhydrophilicity and Superaerophobicity Surface for Solar Cell-Driven Electrocatalytic Water Splitting. *Electrochim. Acta* **2022**, *429*, 140947.

(30) Liang, H.; Li, J.; Zhang, J.; Peng, W.; Li, J.; Liu, J. Tri-Functional Fe-Based Electrocatalyst with Sturdy Three-Dimensional Frame Construction for the ORR, OER and HER. *J. Mater. Chem. A* **2024**, *12* (30), 19344–19351.

(31) Liu, S.-Q.; Gao, M.-R.; Liu, S.; Luo, J.-L. Hierarchically Assembling Cobalt/Nickel Carbonate Hydroxide on Copper Nitride Nanowires for Highly Efficient Water Splitting. *Appl. Catal. B Environ.* **2021**, *292*, 120148.

(32) Liu, Y.; Chen, Y.; Tian, Y.; Sakthivel, T.; Liu, H.; Guo, S.; Zeng, H.; Dai, Z. Synergizing Hydrogen Spillover and Deprotonation by the Internal Polarization Field in a MoS2/NiPS3 Vertical Heterostructure for Boosted Water Electrolysis. *Adv. Mater.* **2022**, *34* (37), 2203615.

(33) Tran, D. T.; Le, H. T.; Kim, N. H.; Lee, J. H. Dual-Coupling Ultrasmall Iron-Ni2P into P-Doped Porous Carbon Sheets Assembled CuxS Nanobrush Arrays for Overall Water Splitting. *Nano Energy* **2021**, *84*, 105861.

(34) Liang, H.; Gandi, A. N.; Anjum, D. H.; Wang, X.; Schwingenschlögl, U.; Alshareef, H. N. Plasma-Assisted Synthesis of NiCoP for Efficient Overall Water Splitting. *Nano Lett.* **2016**, *16* (12), 7718–7725.

(35) Yao, Q.; Huang, B.; Zhang, N.; Sun, M.; Shao, Q.; Huang, X. Channel-rich RuCu Nanosheets for PH-universal Overall Water Splitting Electrocatalysis. *Angew. Chemie* **2019**, *131* (39), 14121–14126.

(36) Hu, Y.; Wang, C.; Liu, Y.; Lin, H.; Zhang, K. Hollow Structural Materials Derived from a MOFs/Polymer Loaded CoRu Alloy for Significantly Boosting Electrochemical Overall Water Splitting. *J. Mater. Chem. A* **2024**.

(37) Du, C.; Dinh, K. N.; Liang, Q.; Zheng, Y.; Luo, Y.; Zhang, J.; Yan, Q. Self-assemble and in Situ Formation of Ni1− XFexPS3 Nanomosaic-decorated MXene Hybrids for Overall Water Splitting. *Adv. Energy Mater.* **2018**, *8* (26), 1801127.

(38) Doan, T. L. L.; Nguyen, D. C.; Prabhakaran, S.; Kim, D. H.; Tran, D. T.; Kim, N. H.; Lee, J. H. Single-atom Co-decorated MoS2 Nanosheets Assembled on Metal Nitride Nanorod Arrays as an Efficient Bifunctional Electrocatalyst for PH-universal Water Splitting. *Adv. Funct. Mater.* **2021**, *31* (26), 2100233.

(39) Chai, L.; Liu, S.; Pei, S.; Wang, C. Electrodeposited Amorphous Cobalt-Nickel-Phosphide-Derived Films as Catalysts for Electrochemical Overall Water Splitting. *Chem. Eng. J.* **2021**, *420*, 129686.

(40) Kim, Y.; Kim, D.; Lee, J.; Lee, L. Y. S.; Ng, D. K. P. Tuning the Electrochemical Properties of Polymeric Cobalt Phthalocyanines for Efficient Water Splitting. *Adv. Funct. Mater.* **2021**, *31* (41), 2103290.
